# Interpreting SNP heritability in admixed populations

**DOI:** 10.1101/2023.08.04.551959

**Published:** 2023-08-04

**Authors:** Jinguo Huang, Saonli Basu, Mark D. Shriver, Arslan A. Zaidi

**Affiliations:** 1 Bioinformatics and Genomics, Huck Institutes of the Life Sciences, Pennsylvania State University; 2 Department of Anthropology, Pennsylvania State University; 3 Department of Biostatistics, University of Minnesota; 4 Department of Genetics, Cell Biology, and Development, University of Minnesota; 5 Institute of Health Informatics, University of Minnesota

## Abstract

SNP heritability $\left(h_{snp}^{2}\right)$ is defined as the proportion of phenotypic variance explained by genotyped SNPs and is believed to be a lower bound of heritability $\left(h^{2}\right)$, being equal to it if all causal variants are known. Despite the simple intuition behind $h_{snp}^{2}$, its interpretation and equivalence to $h^{2}$ is unclear, particularly in the presence of population structure and assortative mating. It is well known that population structure can lead to inflation in ${\overset{ˆ}{h}}_{snp}^{2}$ estimates. Here we use analytical theory and simulations to demonstrate that estimates of $h_{snp}^{2}$ are not guaranteed to be equal to $h^{2}$ in admixed populations, even in the absence of confounding and even if the causal variants are known. We interpret this discrepancy arising not because the estimate is biased, but because the estimand itself as defined under the random effects model may not be equal to $h^{2}$. The model assumes that SNP effects are uncorrelated which may not be true, even for unlinked loci in admixed and structured populations, leading to over- or under-estimates of $h_{snp}^{2}$ relative to $h^{2}$. For the same reason, local ancestry heritability $\left(h_{\gamma }^{2}\right)$ may also not be equal to the variance explained by local ancestry in admixed populations. We describe the quantitative behavior of $h_{snp}^{2}$ and $h_{\gamma }^{2}$ as a function of admixture history and the genetic architecture of the trait and discuss its implications for genome-wide association and polygenic prediction.

## Introduction

The ability to estimate heritability $\left(h^{2}\right)$ from unrelated individuals was a major advance in genetics. Traditionally, $h^{2}$ was estimated from family-based studies in which the phenotypic resemblance between relatives could be modeled as a function of their expected genetic relatedness [1]. But this approach was limited to analysis of closely related individuals where pedigree information is available and the realized genetic relatedness is not too different from expectation [2]. With the advent of genome-wide association studies (GWAS), we hoped that many of the variants underlying this heritability would be uncovered. But when genome-wide significant SNPs explained a much smaller fraction of the phenotypic variance, it became important to explain the missing heritability – were family-based estimates inflated or were GWAS just underpowered, limited by variant discovery?

Yang *et al.* (2010) [3] made the key insight that one could estimate the portion of $h^{2}$ tagged by genotyped SNPs, regardless of whether or not they were genome-wide significant, by exploiting the subtle variation in the realized genetic relatedness among apparently unrelated individuals [3–5]. This quantity came to be known colloquially as ‘SNP heritability’ ${(h}_{snp}^{2})$ and it is believed to be equal to $h^{2}$ if all causal variants are included among genotyped SNPs [3]. Indeed, estimates of $h_{snp}^{2}$ explain a much larger fraction of trait heritability [3], approaching family-based estimates of $h^{2}$ when whole genome sequence data are used [6]. This has made it clear that GWAS have yet to uncover more variants with increasing sample size. Now, $h_{snp}^{2}$ has become an important aspect of the design of genetic studies and is often used to define the power of variant discovery in GWAS and the upper limit of polygenic prediction accuracy.

Despite the utility and simple intuition of $h_{snp}^{2}$, there is much confusion about its interpretation and equivalence to $h^{2}$, particularly in the presence of population structure and assortative mating [7–12]. But much of the discussion of heritability in structured populations has focused on biases in ${\overset{ˆ}{h}}_{snp}^{2}$ – the estimator – due to confounding effects of shared environment and linkage disequilibrium (LD) with other variants [7, 9–11, 13]. There is comparatively little discussion, at least in human genetics, on the fact that LD due to population structure also contributes to genetic variance, and therefore, is a component of heritability [1] (but see also [14, 15]). We think this is at least partly due to the fact that most studies are carried out in cohorts with primarily European ancestry, where the degree of population structure is minimal and large effects of LD can be ignored. But that is not the case for diverse, multi-ethnic cohorts, which have historically been underrepresented in genetic studies, but thanks to a concerted effort in the field, are now becoming increasingly common [16–22]. The complex structure in these cohorts also brings unique methodological challenges and it is imperative that we understand whether existing methods, which have largely been evaluated in more homogeneous groups, generalize to more diverse cohorts.

Our goal in this paper is to study the behavior of $h_{snp}^{2}$ in admixed populations in relation to $h^{2}$. Should we expect the two to be equal in the ideal situation where causal variants are known? If not, how should we interpret $h_{snp}^{2}$? To answer these questions, we derived a general expression for the genetic variance in admixed populations, decomposing it in terms of the contribution of population structure, which influences both the genotypic variance at individual loci and the LD across loci. We used extensive simulations, where the ground truth is known, to test if $h_{snp}^{2}$ estimated with genome-wide restricted maximum likelihood (GREML) [3, 5] – arguably still the most widely used method – is equal to $h^{2}$ and explore this equivalence as a function of admixture history and genetic architecture. We show that GREML-based ${\overset{ˆ}{h}}_{snp}^{2}$ is not guaranteed to be equal to $h^{2}$ in admixed and other structured populations, even in the absence of confounding and when all causal variants are known. We explain this discrepancy in terms of the generative model underlying GREML, which assumes that (i) the effect of causal variants are random and uncorrelated, and (ii) the population is mating randomly. As a result, the GREML estimand $\left(h_{snp}^{2}\right)$ by definition equals $h^{2}$ only if these conditions are met. We discuss the implications of this for GWAS and polygenic prediction accuracy.

## Model

### Genetic architecture

We begin by describing a generative model for the phenotype. Let y=g+e, where y is the phenotypic value of an individual, g is the genotypic value, and e is random error. We assume additive effects such that $g=\sum_{i=1}^{m}\;\beta_{i}x_{i}$ where $\beta_{i}$ is the effect size of the $i^{th}$ biallelic locus and $x_{i}\in \{0,\;1,\;2\}$ is the number of copies of the trait-increasing allele. Importantly, the effect sizes are fixed quantities and differences in genetic values among individuals are due to variation in genotypes. Note, that this is different from the model assumed by GREML where genotypes are fixed and effect sizes are random [14].

We denote the mean, variance, and covariance with E(.),V(.), and C(.), respectively, where the expectation is measured over random draws from the population rather than random realizations of the evolutionary process. We can express the additive genetic variance of a quantitative trait as follows:

$$V_{g}=V\left(\sum_{i=1}^{m}\;\;\beta_{i}x_{i}\right)=\sum_{i=1}^{m}\;\beta_{i}^{2}V\left(x_{i}\right)+\sum_{j\neq i}\;\beta_{i}\beta_{j}C\left(x_{i},x_{j}\right)$$

Here the first term represents the contribution of individual loci (genic variance) and the second term is the contribution of linkage disequilibrium (LD contribution). We make the assumption that loci are unlinked and therefore, the LD contribution is entirely due to population structure. We describe the behavior of $V_{g}$ in a population that is a mixture of two previously isolated populations A and B that diverged from a common ancestor. To do this, we denote θ as the fraction of the genome of an individual with ancestry from population A. Thus, θ=1 if the individual is from population A, 0 if they are from population B, and θ∈(0,1) if they are admixed. Then, $V_{g}$ can be expressed in terms of ancestry as (Appendix):

(1.1) $$V_{g}=2\;E(\theta )\sum_{i=1}^{m}\;\;\beta_{i}^{2}f_{i}^{A}(1-f_{i}^{A})+2\{1-E(\theta )\}\sum_{i=1}^{m}\;\;\beta_{i}^{2}f_{i}^{B}(1-f_{i}^{B})$$

(1.2) $$\;+2\;E(\theta )\{1-E(\theta )\}\sum_{i=1}^{m}\;\;\beta_{i}^{2}{\left(f_{i}^{A}-f_{i}^{B}\right)}^{2}$$

(1.3) $$\;+2\;V(\theta )\sum_{i=1}^{m}\;\;\beta_{i}^{2}{\left(f_{i}^{A}-f_{i}^{B}\right)}^{2}$$

(1.4) $$\;+4\;V\left(\theta \right)\sum_{i\neq j}\beta_{i}\beta_{j}\left(f_{\text{i}}^{\text{A}}-f_{\text{i}}^{\text{B}}\right)\left(f_{\text{j}}^{\text{A}}-f_{\text{j}}^{\text{B}}\right)$$

where $f_{i}^{A}$ and $f_{i}^{B}$ are the allele frequencies in populations A and B, and E(θ) and V(θ) are the mean and variance of individual ancestry. The first three terms represent the sum of the genic variance and the last term represents the LD contribution.

### Demographic history

From Eq. 1, it is clear that, conditional on the genetic architecture in the source populations $\left(\beta ,f^{A},f^{B}\right)$, $V_{g}$ in the admixed populations is a function of the mean, E(θ), and variance, V(θ), of individual ancestry. We consider two demographic models that affect E(θ) and V(θ) in qualitatively different ways. In the first model, the source populations meet once t generations ago (we refer to this as t=0) in proportions p and 1-p, after which there is no subsequent admixture (Fig. 2A). In the second model, there is continued gene flow in every generation from one of the source populations such that the mean overall amount of ancestry from population A is the same as in the first model (Fig. 2A). For brevity, we refer to these as the hybrid-isolation (HI) and continuous gene flow (CGF) models, respectively, following Pfaff et al. (2001) [23]. V(θ) is also affected by assortative mating based on ancestry (hereafter referred to as assortative mating for bervity) and we model this following Zaitlen et al. (2017) using a parameter P∈(0,1), which represents the correlation in the ancestry of individuals in a mating pair [24].

**Figure 1:**
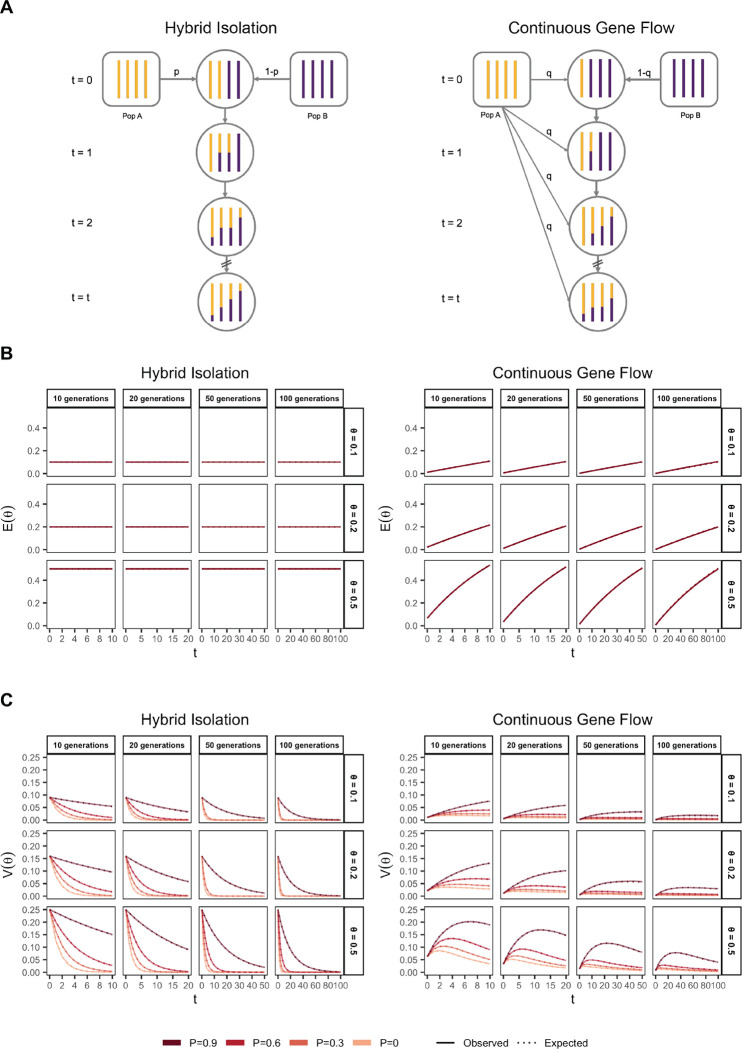
The behavior of mean and variance of individual ancestry as a function of admixture history. (A) Shows the demographic models under which simulations were carried out. Admixture might occur once (Hybrid Isolation, HI, left column) or continuously (Continuous Gene Flow, CGF, right column). (B) The mean individual ancestry, E(θ) remains constant over time in the HI model and increases in the CGF model with continued gene flow. (C) The variance in individual ancestry, V(θ) is maximum at t=0, decaying subsequently. V(θ) increases with gene flow in the CGF model and will subsequently decrease with time. P measures the strength of assortative mating, which slows the decay of V(θ).

Under these conditions, the behavior of E(θ) and V(θ) has been described previously [24, 25] (Fig. 1A and B). Briefly, in the HI model, E(θ) remains constant at p in the generations after admixture as there is no subsequent gene flow. V(θ) is at its maximum at t=0 when all individuals either carry chromosomes from population A or B, but not both. This genome-wide correlation in ancestry breaks down in subsequent generations as a function of mating, independent assortment, and recombination, leading to a decay in V(θ), the rate depending on the strength of assortative mating (Fig. 1). In the CGF model, both E(θ) and V(θ) increase with time as new chromosomes are introduced from the source populations. But while E(θ) continues to increase monotonically, V(θ) will plateau and decrease due to the countervailing effects of independent assortment and recombination which redistribute ancestry in the population, reaching equilibrium at zero if there is no more gene flow and the population is mating randomly. V(θ) provides an intuitive and quantitative measure of the degree of population structure (along the axis of ancestry) in admixed populations.

## Results

### Genetic variance in admixed populations

**Figure 2:**
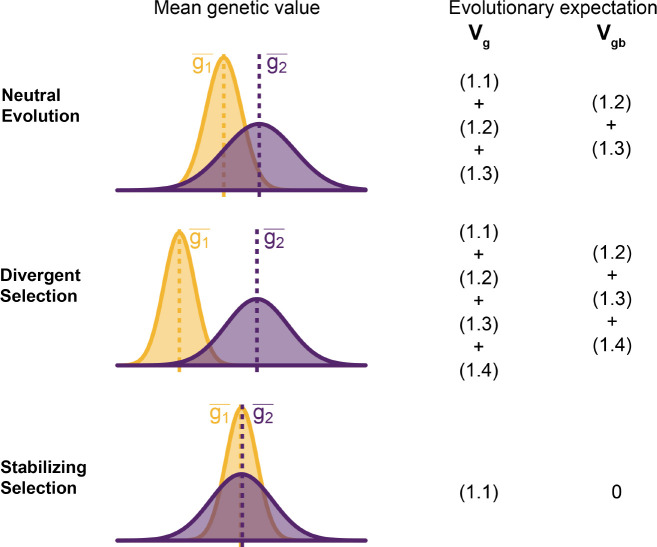
Decomposing genetic variance in a two-population system. The plot illustrates the expected distribution of genetic values in two populations under different selective pressures and the terms on the right list the total $\left(V_{g}\right)$ and between-population genetic variance $\left(V_{gb}\right)$ expected over the evolutionary process. For neutrally evolving traits (top row), we expect there to be an absolute difference in the mean genetic values $\left(\left|\overset{-}{g_{1}}-\overset{-}{g_{2}}\right|\right)$ that is proportional to $F_{ST}$. For traits under divergent selection (middle), $\left|\overset{-}{g_{1}}-\overset{-}{g_{2}}\right|$ is expected to be greater than that expected under genetic drift. For traits under stabilizing selection, $\left|\overset{-}{g_{1}}-\overset{-}{g_{2}}\right|$ will be less than that expected under genetic drift, and zero in the extreme case.

To understand the expectation of genetic variance in admixed populations, it is first worth discussing its behavior in the source populations. In Eq. 1, the first term represents the within-population component $\left(V_{gw}\right)$ and the last three terms altogether represent the component of genetic variance between populations A and B $\left(V_{gb}\right)$. Note that $V_{gb}=\frac{{\left(\overset{-}{g_{1}}-\overset{-}{g_{2}}\right)}^{2}}{2}$ is positive only if there is a difference in the mean genotypic values (Fig. 2B). This variance increases with the degree of genetic drift since the expected values of both ${\left(f_{i}^{A}-f_{i}^{B}\right)}^{2}$ and $\left(f_{i}^{A}-f_{i}^{B})(f_{j}^{A}-f_{j}^{B}\right)$ are functions of $F_{ST}$. But while ${\left(f_{i}^{A}-f_{i}^{B}\right)}^{2}$ is expected to increase monotonically with increasing genetic drift, $\left(f_{i}^{A}-f_{i}^{B})(f_{j}^{A}-f_{j}^{B}\right)$ is expected to be zero under neutrality because the direction of frequency change will be uncorrelated across loci. In this case, the LD contribution, i.e., (1.4), is expected to be zero and $V_{gb}=(1.1)+(1.2)+(1.3)$. However, this is true only in expectation over the evolutionary process and the realized LD contribution may be non-zero even for neutral traits.

For traits under selection, the LD contribution is expected to be greater or less than zero, depending on the type of selection. Under divergent selection, trait-increasing alleles will be systematically more frequent in one population over the other, inducing positive LD across loci, increasing the LD contribution, i.e., term (1.4). Stabilizing selection, on the other hand, induces negative LD, reducing (1.4) [26, 27]. In the extreme case, the mean genetic values of the two populations are exactly equal and $V_{gb}=(1.2)+(1.3)+(1.4)=0$. For this to be true, (1.4) has to be negative and equal to (1.2)+(1.3), which are both positive, and the total genetic variance is reduced to the within-population variance, i.e., term (1.1) (Fig. 2). This is relevant because, as we show in the following sections, the behavior of the genetic variance in admixed populations depends on the magnitude of $V_{gb}$ between the source populations.

We illustrate this by tracking the genetic variance in admixed populations for two traits, both with the same mean $F_{ST}$ at causal loci but with different LD contributions (term 1.4): one where the LD contribution is positive (Trait 1) and the other where it is negative (Trait 2). Thus, traits 1 and 2 can be thought of as examples of phenotypes under divergent and stabilizing selection, respectively, and we refer to them as such from hereon. To simulate the genetic variance of such traits, we drew the allele frequencies ($f^{A}$ and $f^{B}$) in populations A and B for 1,000 causal loci with $F_{ST}∼0.2$ using the Balding-Nichols model [28]. We drew their effects (β) from $𝒩(0,\frac{1}{2\overset{‾}{f}(1-\overset{‾}{f})})$ where $\overset{‾}{f}$ is the mean allele frequency between the two populations. To simulate positive and negative LD, we permuted the effect signs across variants 100 times and selected the combinations that gave the most positive and negative LD contribution to represent the genetic architecture of traits that might be under directional (Trait 1) and stabilizing (Trait 2) selection, respectively (Methods). We simulated the genotypes of 10,000 individuals under the HI and CGF models for t∈{10,20,50,100} generations post-admixture and calculated genetic values for both traits using $g=\sum_{i=1}^{m}\;\beta_{i}x_{i}$, where m=1,000 (Method). The observed genetic variance at any time can then be calculated simply as the variance in genetic values, i.e. $V_{g}=V(g)$.

**Figure 3:**
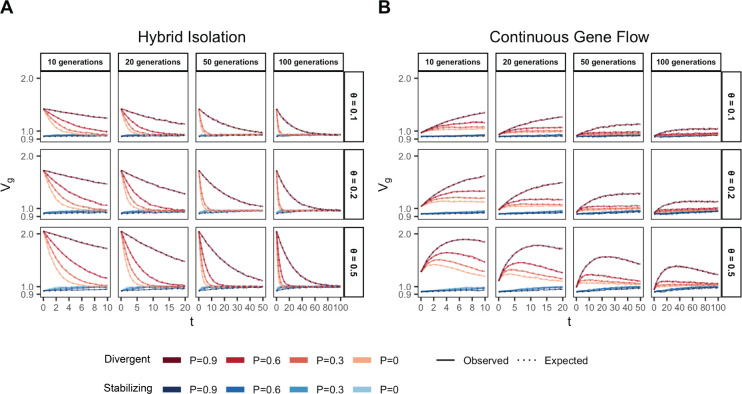
Genetic variance in admixed populations under the (A) HI and (B) CGF models. Solid lines represent the expected genetic variance based on Eq. (1) and dashed lines represent results of simulations averaged over ten replicates. Red and blue lines represent traits under divergent and stabilizing selection, respectively.

In the HI model, E(θ) does not change (Fig. 1) so terms (1.1) and (1.2) are constant through time. Terms (1.3) and (1.4) decay towards zero as the variance in ancestry goes to zero and $V_{g}$ ultimately converges to (1.1)+(1.2) (Fig. 3). This equilibrium value is equal to the $E\left(V_{g}|\theta \right)$ (Appendix) and the rate of convergence depends on the strength of assortative mating, which slows the rate at which V(θ) decays. $V_{g}$ approaches equilibrium from a higher value for traits under divergent selection and lower value for traits under stabilizing selection because of positive and negative LD contributions, respectively, at t=0 (Fig. 3). In the CGF model, $V_{g}$ increases initially for both traits with increasing gene flow (Fig. 3). This might seem counter-intuitive at first because gene flow increases admixture LD, which leads to more negative values of the LD contribution for traits under stabilizing selection (Fig. S1). But this is outweighed by positive contributions from the genic variance – terms (1.1)+(1.2)+(1.3) – all of which initially increase with gene flow (Fig. S1). After a certain point, the increase in $V_{g}$ slows down as any increase in V(θ) due to gene flow is counterbalanced by recombination and independent assortment. Ultimately, $V_{g}$ will decrease if there is no more gene flow, reaching the same equilibrium value as in the HI model, i.e., $E\left(V_{g}|\theta \right)=(1.1)+(1.2)$. Because the loci are unlinked, we refer to the sum (1.3)+(1.4) as the contribution of population structure.

### GREML estimation of $h_{snp}^{2}$

In their original paper, Yang et al. (2010) defined $h_{snp}^{2}$ as the variance explained by genotyped SNPs and not as heritability [3]. This is because $h^{2}$ is the genetic variance explained by causal variants, which are unknown. Genotyped SNPs may not overlap with or tag all causal variants and thus, $h_{snp}^{2}$ is understood to be a lower bound of $h^{2}$, both being equal if causal variants are known [3]. Our goal is to demonstrate that this may not be true in structured populations and quantify the discrepancy between $h_{snp}^{2}$ and $h^{2}$, even in the ideal situation when causal variants are known.

We used GREML, implemented in GCTA [3, 5], to estimate the genetic variance for our simulated traits. GCTA assumes the following model: y=Zu+ϵ where Z is an n×m standardized genotype matrix such that the genotype of the $k^{th}$ individual at the $i^{th}$ locus is $z_{ik}=\frac{x_{ik}-2f_{i}}{\sqrt{2f_{i}\left(1-f_{i}\right)}}$, $f_{i}$ being the allele frequency. The SNP effects are assumed to be random and independent such that $u∼𝒩(0,I\frac{\sigma_{u}^{2}}{m})$ and $\epsilon ∼𝒩\left(0,I\sigma_{\epsilon }^{2}\right)$ is random environmental error. Then, the phenotypic variance can be decomposed as:

$$V(y)\;=V(Zu)+V(e)\;\;=\frac{ZZ^{\prime }}{m}\sigma_{u}^{2}+\sigma_{\epsilon }^{2}$$

where $\frac{ZZ^{\prime }}{m}$ is the genetic relationship matrix (GRM), the variance components ${\overset{ˆ}{\sigma }}_{u}^{2}$ and ${\overset{ˆ}{\sigma }}_{\epsilon }^{2}$ are estimated using restricted maximum likelihood, and ${\overset{ˆ}{h}}_{snp}^{2}$ is calculated as $\frac{{\overset{ˆ}{\sigma }}_{u}^{2}}{{\overset{ˆ}{\sigma }}_{u}^{2}+{\overset{ˆ}{\sigma }}_{\epsilon }^{2}}$. Really, the key estimate is ${\overset{ˆ}{\sigma }}_{u}^{2}$ since ${\overset{ˆ}{\sigma }}_{\epsilon }^{2}=1-{\overset{ˆ}{\sigma }}_{u}^{2}$, and we are interested in asking whether ${\overset{ˆ}{\sigma }}_{u}^{2}$ is equal to $V_{g}$. To answer this, we constructed the GRM with causal variants and estimated ${\overset{ˆ}{\sigma }}_{u}^{2}$ using GCTA, including individual ancestry in the model as a fixed effect to correct for any confounding due to genetic stratification [3, 4].

**Figure 4:**
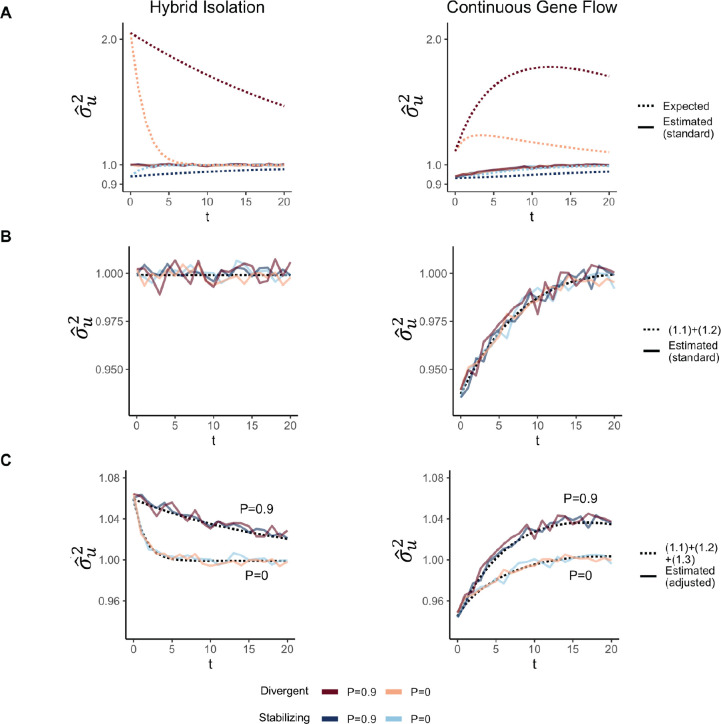
The behavior of GREML estimates of the genetic variance $\left({\overset{ˆ}{\sigma }}_{u}^{2}\right)$ using global ancestry as a covariate in admixed populations under the HI (left column) and CGF (right column) models. The solid lines represent values observed in simulations averaged across ten replicates and the dotted lines represent the expected values based on Eq. 1. Red and blue lines represent values for traits under divergent and stabilizing selection, respectively. P indicates the strength of assortative mating. (A) and (B) shows the behavior of ${\overset{ˆ}{\sigma }}_{u}^{2}$ using the standard $\sqrt{2f(1-f)}$ genotype scaling. In (C), we show ${\overset{ˆ}{\sigma }}_{u}^{2}$ when genotypes are scaled by the square root of their sample variance, i.e., $\sqrt{V(x)}$.

We show that GCTA under- and over-estimates the genetic variance in admixed populations for traits under divergent (Trait 1) and stabilizing selection (Trait 2), respectively, when there is population structure, i.e., when V(θ)>0 (Fig. 4A). One reason for this bias is that the GREML model assumes that the effects are independent, which, as discussed in the previous section, is not true for traits under divergent or stabilizing selection between the source populations, and only true for neutral traits in expectation. Because of this, ${\overset{ˆ}{\sigma }}_{u}^{2}$ does not capture the LD contribution, i.e. term (1.4) (Fig. S3A). In our simulations, the LD across loci is entirely due to population structure since they are unlinked. Thus, the LD contribution, and therefore, the bias in ${\overset{ˆ}{\sigma }}_{u}^{2}$ is larger in the presence of population structure.

But ${\overset{ˆ}{\sigma }}_{u}^{2}$ can be biased, even if the effects are uncorrelated and the LD contribution is zero. It is standard in GREML to scale genotypes with $\sqrt{2f_{i}\left(1-f_{i}\right)}$ where $f_{i}$ is the frequency of the allele in the population. This scaling assumes that $V\left(x_{i}\right)=2f_{i}\left(1-f_{i}\right)$, which is true only if the population were mating randomly. In an admixed population $V\left(x_{i}\right)=2f_{i}\left(1-f_{i}\right)+2V(\theta ){\left(f_{i}^{A}-f_{i}^{B}\right)}^{2}$, where $f_{i},f_{i}^{A}$, and $f_{i}^{B}$ correspond to frequency in the admixed population, and source populations, A and B, respectively. We show that this assumption biases ${\overset{ˆ}{\sigma }}_{u}^{2}$ downwards by a factor of $2V(\theta ){\left(f_{i}^{A}-f_{i}^{B}\right)}^{2}-$ term (1.3) (Fig. S3B, Appendix). We confirm this by showing that we can fix this bias if we scale genotypes by their sample variance, i.e., $\sqrt{V\left(x_{i}\right)}$ (Fig. S3C) (Appendix). Thus, with the standard scaling, ${\overset{ˆ}{\sigma }}_{u}^{2}$ is not even equal to the genic variance in the presence of population structure.

The overall bias in ${\overset{ˆ}{\sigma }}_{u}^{2}$ is determined by the relative magnitude and direction of terms (1.3) and (1.4), both of which are functions of V(θ), and therefore, of the degree of structure in the population. If there is no more gene flow, V(θ) will ultimately go to zero and $V_{g}$ will converge towards ${\overset{ˆ}{\sigma }}_{u}^{2}$. But note that even though ${\overset{ˆ}{\sigma }}_{u}^{2}$ may be biased relative to $V_{g}$, it is not biased relative to the estimand under the model assumed by GREML $\left(\sigma_{u}^{2}\right)$, which is more accurately interpreted as the genetic variance expected if there were no correlation in effect sizes and if the population were mating randomly. In other words, $E\left({\overset{ˆ}{\sigma }}_{u}^{2}\right)=\sigma_{u}^{2}=(1.1)+(1.2)\neq V_{g}$ (Fig. S3B)

### Local ancestry heritability

A related quantity of interest in admixed populations is local ancestry heritability $\left(h_{\gamma }^{2}\right)$, which is defined as the proportion of phenotypic variance that can be explained by local ancestry. Zaitlen et al. (2014) [29] showed that this quantity is related to, and can be used to estimate, $h_{snp}^{2}$ in admixed populations. The advantage of this ‘indirect’ approach is that local ancestry segments shared between individuals are identical by descent and are therefore, more likely to tag causal variants compared to array markers, allowing one to potentially capture the contributions of rare and structural variants [29]. Here, we show that under the random effects model, $h_{\gamma }^{2}$ may not be equal to the local ancestry ancestry heritability in the population because of the same reasons that $h_{snp}^{2}$ is not equal to the heritability.

We define local ancestry $\gamma_{i}\in \{0,\;1,\;2\}$ as the number of alleles at locus i that trace their ancestry to population A. Thus, ancestry at the $i^{\text{th}}$ locus in individual k is a binomial random variable with $E\left(\gamma_{ik}\right)=2\theta_{k}$. Similar to the genetic value of an individual, we define ‘ancestry value’ as $\sum_{i=1}^{m}\;\phi_{i}\gamma_{i}$, where $\phi_{i}=\beta_{i}(f_{i}^{A}-f_{i}^{B})$ is the effect size of local ancestry (Appendix). Then, the genetic variance due to local ancestry can be expressed as:

$$V_{\gamma }\;=V(\sum_{i=1}^{m}\;\;\phi_{i}\gamma_{i})=\sum_{i=1}^{m}\;\;\phi_{i}^{2}V\left(\gamma_{i}\right)+\sum_{i=1}^{m}\;\;\sum_{j\neq i}\;\;\phi_{i}\phi_{j}C\left(\gamma_{i},\gamma_{j}\right)\;\;=2E(\theta )\{1-E(\theta )\}\sum_{i=1}^{m}\;\;\phi_{i}^{2}+2V(\theta )\sum_{i=1}^{m}\;\;\phi_{i}^{2}+4V(\theta )\sum_{i=1}^{m}\;\;\sum_{j\neq i}\;\;\phi_{i}\phi_{j}\;\;=2E(\theta )\{1-E(\theta )\}\sum_{i=1}^{m}\;\;\beta_{i}^{2}{\left(f_{i}^{A}-f_{i}^{B}\right)}^{2}\;\;+2V(\theta )\sum_{i=1}^{m}\;\;\beta_{i}^{2}{\left(f_{i}^{A}-f_{i}^{B}\right)}^{2}\;\;+4V(\theta )\sum_{i=1}^{m}\;\;\sum_{j\neq i}\;\;\beta_{i}\beta_{j}(f_{i}^{A}-f_{i}^{B})(f_{j}^{A}-f_{j}^{B})$$

and heritability explained by local ancestry is simply the ratio of $V_{\gamma }$ and the phenotypic variance. Note that $V_{\gamma }=(1.2)+(1.3)+(1.4)$ – the genetic variance between the source populations – and therefore its behavior is similar to $V_{g}$ in that the terms (1.3) and (1.4) decay towards zero as V(θ)→0, and $V_{\gamma }$ converges to (1.2) (Fig. S2).

GREML estimation of $h_{\gamma }^{2}$ is similar to the estimation of $h_{snp}^{2}$, the key difference being that the former involves constructing the GRM using local ancestry instead of genotypes [29]. The following model is assumed: y=Wv+ξ where W is an n×m standardized local ancestry matrix, $v∼𝒩(0,I\frac{\sigma_{v}^{2}}{m})$ are local ancestry effects, and $\xi ∼𝒩(0,I\sigma_{\xi }^{2})$. The phenotypic variance is decomposed as $V(y)=V(Wv)+V(\xi )=\frac{WW^{\prime }}{m}\sigma_{v}^{2}+\sigma_{\xi }^{2}$ where $\frac{WW^{\prime }}{m}$ is the local ancestry GRM and $\sigma_{v}^{2}$ is the parameter of interest, which is believed to be equal to $V_{\gamma }$ – the genetic variance due to local ancestry. We show here that this equivalence is not guaranteed in the presence of population structure and/or correlated effects.

**Figure 5:**
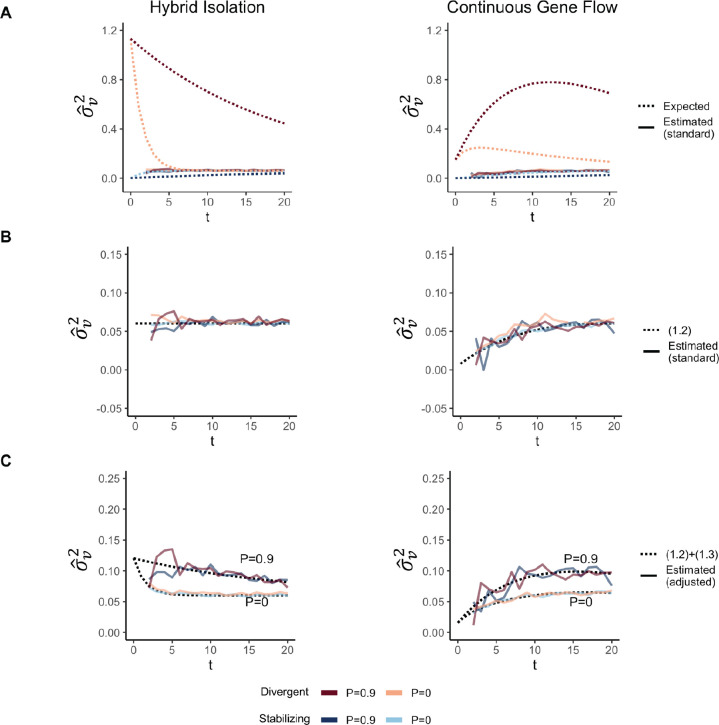
The behavior of GREML estimates of the variance due to local ancestry $\left({\overset{ˆ}{\sigma }}_{v}^{2}\right)$ using global ancestry as a covariate in admixed populations under the HI (left column) and CGF (right column) models. The solid lines represent values observed in simulations averaged across ten replicates and the dotted lines represent the expected values based on Eq. 1. Red and blue lines represent values for Traits 1 and 2, respectively. P indicates the strength of assortative mating. (A) and (B) shows the behavior of ${\overset{ˆ}{\sigma }}_{v}^{2}$ when the default scaling of local ancestry is used. In (C), we show ${\overset{ˆ}{\sigma }}_{v}^{2}$ when local ancestry is scaled with square root of the sampling variance, i.e., $\sqrt{V\left(\gamma_{i}\right)}$.

We calculated the GRM from local ancestry at causal variants with our simulated data, and estimated $\sigma_{v}^{2}$ with individual ancestry as a fixed effect to correct for genetic stratification [29]. We show that, in the presence of population structure, i.e., when $V(\theta )>0,{\overset{ˆ}{\sigma }}_{v}^{2}$ is biased downwards relative to $V_{\gamma }$ for traits under divergent selection and upwards for traits under stabilizing selection (Fig. 5A). In addition, if local ancestry is scaled by its expectation under random mating rather than the square root of the sample variance, ${\overset{ˆ}{\sigma }}_{v}^{2}$ will be underestimated even if the effects are uncorrelated (Fig. 5B). The overall bias is equal to the terms weighted by V(θ)-(1.3)+(1.4). As a result, ${\overset{ˆ}{h}}_{\gamma }^{2}$ is not guaranteed to be equal to the heritability explained by local ancestry, even in the absence of confounding and even if local ancestry at causal variants is known without error. This is not because of an inherent bias in the estimation procedure since $E\left({\overset{ˆ}{\sigma }}_{v}^{2}\right)=\sigma_{v}^{2}=(1.2)$, but because the estimand itself defined under the random-effects model does not capture the variance due to LD and population structure.

We note that if individual ancestry is not included as a covariate, ${\overset{ˆ}{\sigma }}_{v}^{2}$ tends to be biased even relative to $\sigma_{v}^{2}$ due to confounding effects of genetic stratification (Fig. S4). We did not see the same level of confounding when individual ancestry was not included in the model estimating $\sigma_{u}^{2}$ (Fig. S3). We do not fully understand the reason for this but we think it might be because genetic stratification leads to more inflation in the effect size of local ancestry compared to the effect size of genotype.

### How much does population structure contribute in practice?

In the previous sections, we showed theoretically that $h_{snp}^{2}$ is not equal to $h^{2}$ in admixed populations even if the causal variants are known. Ultimately, whether or not this is true in practice is an empirical question, which is difficult to answer because the causal variants, their $F_{ST}$, and the correlation between their effect sizes are unknown. Here, we sought to answer a related question: to what extent does population structure contribute to the variance explained by GWAS SNPs in African Americans? To answer this, we used independent genome-wide significant SNPs for 26 quantitative traits from the GWAS catalog [30]. We calculated the total genetic variance explained and decomposed it into the four components in Equation 1 using allele frequencies ($f^{A}$ and $f^{B}$) from the 1000 Genomes YRI and CEU [31], and the mean (E(θ)≈0.77) and variance (V(θ)≈0.02) of individual ancestry from the 1000 Genomes ASW (Methods).

**Figure 6:**
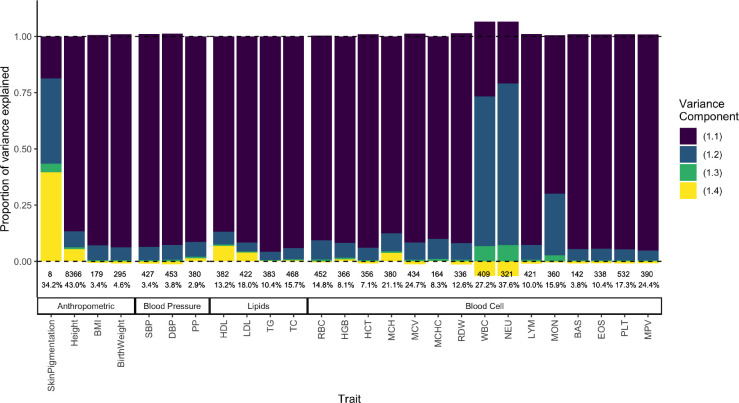
Decomposing the genetic variance explained by GWAS SNPs in African Americans. We calculated the four variance components listed in Equation 1, their values shown on the y-axis as a fraction of the total variance explained (shown as percentage at the bottom). The number of variants used to calculate variance components for each trait is also shown at the bottom.

We show that for skin pigmentation – a trait under strong divergent selection – the LD contribution, i.e. term (1.4), is positive and accounts for ≈ 40 – 50% of the total variance explained. This is because of large allele frequency differences between Africans and Europeans that are correlated across skin pigmentation loci due to strong selection favoring alleles for darker pigmentation in regions with high UV exposure and vice versa [32–35]. But for most other traits, LD contributes relatively little, explaining a modest, but non-negligible proportion of the genetic variance in height, LDL and HDL cholestrol, mean corpuscular hemoglobin (MCH), neutrophil count (NEU), and white blood cell count (WBC) (Fig. 6). Because we selected independent associations for this exercise (Methods), the LD contribution is driven entirely due to population structure among African Americans. The contribution of population structure to the genic variance, i.e., term (1.3) is also small even for traits like skin pigmentation and neutrophil count with large effect alleles that are highly diverged in frequency between Africans and Europeans [33, 34, 36–38]. Overall, this suggests that population structure contributes relatively little, as least to the variance explained by GWAS SNPs.

## Discussion

Despite the growing size of GWAS and discovery of thousands of variants for hundreds of traits [30], the heritability explained by GWAS SNPs remains a fraction of twin-based heritability estimates. Yang et al. (2010) introduced the concept of SNP heritability $\left(h_{snp}^{2}\right)$ that does not depend on the discovery of causal variants but assumes that they are numerous and are more or less uniformly distributed across the genome (the infinitesimal model), their contributions to the genetic variance ‘tagged’ by genotyped SNPs [3]. $h_{snp}^{2}$ is now routinely estimated in most genomic studies and at least for some traits (e.g. height and BMI), these estimates now approach twin-based heritability [6]. But despite the widespread use of $h_{snp}^{2}$, its interpretation remains unclear, particularly its equivalence to heritability in the presence of population structure. It is generally accepted that ${\overset{ˆ}{h}}_{snp}^{2}-$ the estimator – can be biased in structured populations [4, 7, 9–11, 39]. Here, we show how ${\overset{ˆ}{h}}_{snp}^{2}$ may not be equal to $h^{2}$ in admixed populations even in the absence of confounding and even if causal variants are known.

GREML assumes that SNP effects are random and independent – an assumption that may not be true, especially in the presence of admixture and population structure, which create LD across unlinked loci. This LD contributes to the genetic variance and can persist, despite recombination, for a number of generations due to continued gene flow and/or assortative mating. Because the LD contribution can be positive or negative, ${\overset{ˆ}{h}}_{snp}^{2}$ can under- or over-estimate $h^{2}$. But ${\overset{ˆ}{h}}_{snp}^{2}$ can be biased even when effects are uncorrelated if the genotypes are scaled by $\sqrt{2f(1-f)}$ – the standard approach, which implicitly assumes a randomly mating population. In the presence of population structure, the variance in genotypes can be higher and ${\overset{ˆ}{h}}_{snp}^{2}$ does not capture this additional variance. For these reasons, there is no guarantee that ${\overset{ˆ}{h}}_{snp}^{2}$ that will be equal to $h^{2}$, even if the causal variants are known. But technically this is not because the estimate is biased, but because the estimand itself as defined under the random effects model is not equal to the heritability [14, 15]. $h_{snp}^{2}$, assuming the genotypes are scaled properly, is better interpreted as the proportion of phenotypic variance explained by the *genic* variance. We show that $h_{\gamma }^{2}$ as defined under random effects models [29, 40] should be interpreted similarly.

Does the LD contribution to the genetic variance have practical implications? The answer to this depends on how one intends to use SNP heritability. $h_{snp}^{2}$ can be useful in qauntifying the power to detect variants in GWAS where the quantity of interest is the genic variance. But if one is interested in using $h_{snp}^{2}$ to measure the extent to which genetic variation contributes to phenotypic variation, in predicting the response to selection, or in defining the upper limit of polygenic prediction accuracy [2] – applications where the LD contribution is important – then $h_{snp}^{2}$ is technically not the relevant quantity.

Ultimately, the discrepancy between $h_{snp}^{2}$ and $h^{2}$ in practice is an empirical question, the answer to which depends on the degree of population structure (which we can measure) and the genetic architecture of the trait (which we do not know *a priori*). We show that for most traits, the contribution of population structure to the variance explained by GWAS SNPs is modest among African Americans. Thus, if we assume that the genetic architecture of GWAS SNPs represents that of all causal variants, then despite incorrect assumptions, the discrepancy between $h_{snp}^{2}$ and $h^{2}$ should be fairly modest. But this assumption is obviously unrealistic given that GWAS SNPs are common variants that in most cases cumulatively explain a small fraction of trait heritability. We know that rare variants contribute disproportionately to the genic variance because of negative selection [41]. What is their LD contribution? This will become clearer in the near future with the discovery of rare variants through large sequence-based studies [42]. While these are underway, theoretical studies are needed to understand how different selection regimes influence the LD patterns between causal variants - an important aspect of the genetic architecture of complex traits.

One limitation of this research is that we studied only the GREML estimator of $h_{snp}^{2}$ because of its widespread use. There are many estimators, which can be broadly grouped into random- and fixed effect estimators based on how they treat SNP effects [43]. Fixed effect estimators make fewer distributional assumptions but they are not as widely used because they require conditional estimates of all variants – a high-dimensional problem where the number of markers is often far larger than the sample size [44]. This is one reason why random effect estimators, such as GREML, are popular – because they reduce the dimensionality by assuming that the effects are drawn from some distribution where the variance is the only parameter of interest. Fixed effects estimators should be able to capture the LD contribution, in principle, but this is not obvious in practice since the simulations used to evaluate the accuracy of such estimators still assume uncorrelated effects [43–45]. Further research is needed to clarify the interpretation of the different estimators of $h_{snp}^{2}$ in structured populations under a range of genetic architectures.

## Methods

### Simulating genetic architecture

We first drew the allele frequency $\left(f^{0}\right)$ of 1,000 biallelic causal loci in the ancestor of populations A and B from a uniform distribution, U (0.001, 0.999). Then, we simulated their frequency in populations A and B ($f^{A}$ and $f^{B}$) under the Balding-Nichols model [28], such that $f^{A}$, $f^{B}∼Beta(\frac{f^{0}(1-F)}{F},\frac{\left(1-f^{0}\right)(1-F)}{F})$ where F=0.2 is the inbreeding coefficient. We implemented this using code adapted from [46]. To avoid drawing extremely rare alleles, we continued to draw $f^{A}$ and $f^{B}$ until we had 1,000 loci with $f^{A}$, $f^{B}\in (0.01,\;0.99)$

We generated the effect size (β) of each locus by sampling from $𝒩(0,\frac{1}{2m\overset{‾}{f}(1-\overset{‾}{f})})$, where m is the number of loci and $\overset{‾}{f}$ is the mean allele frequency across populations A and B. Thus, rare variants have larger effects than common variants and the total genetic variance sums to 1. Given these effects, we simulated two different traits, one with a large difference in means between populations A and B (Trait 1) and the other with roughly no difference (Trait 2). This was achieved by permuting the signs of the effects 100 times to get a distribution of $V_{gb}$ – the genetic variance between populations. This has the effect of varying the LD contribution without changing the $F_{ST}$ at causal loci. We selected the maximum and minimum of $V_{gb}$ to represent Traits 1 and 2.

### Simulating admixture

We simulated the genotypes, local ancestry, and phenotype for 10,000 admixed individuals per generation under the hybrid isolation (HI) and continuous gene flow (CGF) models by adapting the code from Zaitlen et al. (2017) [24]. We denote the ancestry of a randomly selected individual k with θ, the fraction of their genome from population A. At t=0 under the HI model, we set θ to 1 for individuals from population A and 0 if they were from population B such that E(θ)=p∈{0.1,0.2,0.5} with no further gene flow from either source population. In the CGF model, population B receives a constant amount q from population A in every generation starting at t=0. The mean overall proportion of ancestry in the population is kept the same as the HI model by setting $q=1-(1-p{)}^{\frac{1}{t}}$ where t is the number of generations of gene flow from A. In every generation, we simulated ancestry-based assortative mating by selecting mates such that the correlation between their ancestries is P∈{0,0.3,0.6,0.9} in every generation. We do this by repeatedly permuting individuals with respect to each other until P falls within ±0.01 of the desired value. It becomes difficult to meet this criterion when V(θ) is small (Fig.2C). To overcome this, we relaxed the threshold up to 0.04 for some conditions, i.e., when θ∈{0.1,0.2} and t≥50. We generated expected variance in individual ancestry using the expression in [24]. At time t since admixture, $V\left(\theta_{t}\right)=V\left(\theta_{t-1}\right)\frac{\left(1+P\right)}{2}$ under the HI model where P measures the strength of assortative mating, i.e, the correlation between the ancestry between individuals in a mating pair. Under the CGF model, $V\left(\theta_{t}\right)=q(1-q)E{\left(\theta_{t-1}\right)}^{2}+q(1-q)\left\{1-2E\left(\theta_{t-1}\right)\right\}+(1-q)V\left(\theta_{t-1}\right)\frac{\left(1+P\right)}{2}$ (Appendix).

We sampled the local ancestry at each $i^{\text{th}}$ locus as $\gamma_{i}=\gamma_{if}+\gamma_{im}$ where $\gamma_{im}∼Bin\left(1,\theta_{m}\right),\gamma_{if}∼Bin\left(1,\theta_{f}\right)$ and $\theta_{m}$ and $\theta_{f}$ represent the ancestry of the maternal and paternal chromosome, respectively. The global ancestry of the individual is then calculated as $\theta_{k}=\sum_{i=1}^{m}\;\frac{\gamma_{im}+\gamma_{if}}{2m}$, where m is the number of loci. We sample the genotype $x_{i}$ from a binomial distribution conditioning on local ancestry. Thus, $x_{im}∼Bin(1,f_{i}^{A})$ if $\gamma_{im}=1$ and $x_{im}∼Bin\left(1,f_{i}^{B}\right)$ if $\gamma_{im}=0$ and similarly for $x_{if}$. Then, the genotype can be obtained as the sum of the maternal and paternal genotypes: $x_{i}=x_{im}+x_{ip}$. We calculate the genetic value of each individual as $g=\sum_{i=1}^{m}\;\beta_{i}x_{i}$ and the genetic variance as V(g).

### Heritability estimation with GCTA

We used GCTA to estimate $\sigma_{u}^{2}$ and $\sigma_{v}^{2}$ using the --*reml* and --*reml*-*no-constrain* flags. We could not do this without any error in the genetic values so we simulated individual phenotypes with a heritability of 0.8 by adding random noise $e∼𝒩\left(0,V_{g}/4\right)$ to the genetic value. We used GCTA [5] to construct the standard GRM with the --*make-grm* flag. We also constructed an ‘adjusted’ GRM, where instead of standardizing the genotype of the $i^{th}$ SNP with $\sqrt{2f_{i}\left(1-f_{i}\right)}$, we used $\sqrt{V\left(x_{i}\right)}$. Similarly, the ‘adjusted’ local ancestry GRM was constructed by scaling local ancestry with $\sqrt{V\left(\gamma_{i}\right)}$. For both $\sigma_{u}^{2}$ and $\sigma_{v}^{2}$, we included individual ancestry as a covariate to correct for any confounding due to genetic stratification.

### Estimating variance explained by GWAS SNPs

To decompose the variance explained by GWAS SNPs in African Americans, we needed four quantities: (i) effect sizes of GWAS SNPs, (ii) their allele frequencies in Africans and Europeans, and (iii) the mean and variance of global ancestry in African Americans (Equation 1).

We retrieved the summary statistics of 26 traits from GWAS catalog [30]. Full list of traits and the source papers [47–55] are listed in Table S1. To maximize the number of variants discovered, we chose summary statistics from studies that were conducted in both European and multi-ancestry samples and that reported the following information: effect allele, effect size, p-value, and genomic position. For birth weight, we downloaded the data from the Early Growth Genetics (EGG) consortium website [52] since the version reported on the GWAS catalog is incomplete. For skin pigmentation, we chose summary statistics from the UKB [56] released by the Neale Lab (http://www.nealelab.is/uk-biobank) and processed by Ju and Mathieson [47] to represent effect sizes estimated among individuals of European ancestry. We also selected summary statistics from Lona-Durazo et al. (2019) where effect sizes were meta-analyzed across four admixed cohorts [48]. Lona-Durazo et al. provide summary statistics separately with and without conditioning on rs1426654 and rs35397 – two large effect variants in *SCL24A5* and *SLC45A2*. We used the ‘conditioned’ effect sizes and added in the effects of rs1426654 and rs35397 to estimate genetic variance.

We selected independent hits for each trait by pruning and thresholding with PLINK v1.90b6.21 [57] in two steps as in Ju et al. (2020) [47]. We used the genotype data of GBR from the 1000 genome project [31] as the LD reference panel. We kept only SNPs (indels were removed) that passed the genome-wide significant threshold (--*clump-p1 5e-8*) with a pairwise LD cutoff of 0.05 (--*clump-r2 0.05*) and a physical distance threshold of 250Kb (--*clump-kb 250*) for clumping. Second, we applied a second round of clumping (--*clump-kb 100*) to remove SNPs within 100kb.

When GWAS was carried out separately in different ancestry cohorts in the same study, we used inverse-variance weighting to meta-analyze effect sizes for variants that were genome-wide significant (p-value $\left.<5\times 10^{-8}\right)$ in at least one cohort. This allowed us to maximize the discovery of variants such as the Duffy null allele that are absent among individuals of European ancestry but polymorphic in other populations [38].

We used allele frequencies from the 1000 Genomes CEU and YRI to represent the allele frequencies of GWAS SNPs in Europeans and Africans, respectively, making sure that the alleles reported in the summary statistics matched the alleles reported in the 1000 Genomes. We estimated the global ancestry of ASW individuals (N=74) with CEU and YRI individuals from 1000 genome (phase 3) using ADMIXTURE 1.3.0 [58] with k=2 and used it to calculate the mean (proportion of African ancestry = 0.767) and variance (0.018) of global ancestry in ASW. With the effect sizes, allele frequencies, and the mean and variance in ancestry, we calculated the four components of genetic variance using Equation 1 and expressed them as a fraction of the total genetic variance.

Initially, the multi-ancestry summary statistics for a few traits (NEU, WBC, MON, MCH, BAS) yielded values >1 for the proportion of variance explained. This is likely because, despite LD pruning, some of the variants in the model are not independent and tag large effect variants under divergent selection such as the Duffy null allele, leading to an inflated contribution of LD. We checked this by calculating the pairwise contribution, i.e., $\beta_{i}\beta_{j}(f_{i}^{A}-f_{i}^{B})(f_{j}^{A}-f_{j}^{B})$, of all SNPs in the model and show long-range positive LD between variants on chromosome 1 for NEU, WBC, and MON, especially with the Duffy null allele (Fig. S5). A similar pattern was observed on chromosome 16 for MCH, confirming our suspicion. This also suggests that for certain traits, pruning and thresholding approaches are not guaranteed to yield independent hits. To get around this problem, we retained only one association with the lowest p-value, each from chromosome 1 (rs2814778 for NEU, WBC, and MON) and chromosome 16 (rs13331259 for MCH) (Fig. S5). For BAS, we observed that the variance explained was driven by a rare variant (rs188411703, MAF = 0.0024) of large effect (β=-2.27) We believe this effect estimate to be inflated and therefore, we removed it from our calculation.

As a sanity check, we independently estimated the genetic variance as the variance in polygenic scores, calculated using --*score-sum* flag in PLINK, [57] in ASW individuals. We compared the first estimate of the genetic variance to the second (Fig. S6) to confirm two things: (i) the allele frequencies, and mean and variance in ancestry are estimated correctly, and (ii) the variants are more or less independent in that they do not absorb the effects of other variants in the model. We show that the two estimates of the genetic variance are strongly correlated (r∼0.85, Fig. S6).

## Supplementary Material

Supplement 1

## Appendix

### Variance in ancestry

We denote variance and covariance with V(.) and C(.) and used the expressions in [24] to generate the expected value for the variance in ancestry, i.e., V(θ). This is straightforward for the HI model, where at time $t\;V\left(\theta_{t}\right)=V\left(\theta_{t-1}\right)\frac{\left(1+P_{t-1}\right)}{2}$. $P=Cor\left(\theta_{m},\theta_{f}\right)$ measures the strength of assortative mating, i.e, the correlation between the ancestry in a mating pair $\left(\theta_{m},\theta_{f}\right)$. Since our notation slightly differs from [24], we re-derived the expression for $V\left(\theta_{t}\right)$ for the CGF model where population B receives a constant amount q of gene flow from population A in every generation. Note, that $E\left(\theta_{t}\right)=q+(1-q)E\left(\theta_{t-1}\right)$. Then,

$$V\left(\theta_{t}\right)\;=E\left(\theta_{t}^{2}\right)-E{\left(\theta_{t}\right)}^{2}\;\;=q\;+\;(1\;-\;q)E\left[\left(\frac{\theta_{t-1}^{m}\;+\;\theta_{t-1}^{f}}{2}\right)\left(\frac{\theta_{t-1}^{m}\;+\;\theta_{t-1}^{f}}{2}\right)\right]-{\left\{q+(1\;-\;q)E\left(\theta_{t-1}\right)\right\}}^{2}\;\;=q+\frac{\left(1\;-\;q\right)}{4}\{2E(\theta_{t-1}^{2})+2E(\theta_{t-1}^{m}\theta_{t-1}^{f}))-\{q^{2}+2q(1\;-\;q)E(\theta_{t-1})+(1\;-\;q{)}^{2}E{\left(\theta_{t-1}\right)}^{2}\}\;\;=q+\frac{1\;-\;q}{2}E(\theta_{t-1}^{2})+\frac{1\;-\;q}{2}E(\theta_{t-1}^{m}\theta_{t-1}^{f})-q^{2}-2q(1\;-\;q)E(\theta_{t-1})-(1\;-\;q{)}^{2}E{\left(\theta_{t-1}\right)}^{2}\;\;=q(1\;-\;q)+\frac{1\;-\;q}{2}\{V(\theta_{t-1})+E{\left(\theta_{t-1}\right)}^{2}\}+\frac{1\;-\;q}{2}\{C(\theta_{t-1}^{m},\theta_{t-1}^{f})+E{\left(\theta_{t-1}\right)}^{2}\}-2q(1\;-\;q)E(\theta_{t-1})-E{\left(\theta_{t-1}\right)}^{2}\;\;=q(1\;-\;q)+\frac{1\;-\;q}{2}V(\theta_{t-1})+\frac{1\;-\;q}{2}E{\left(\theta_{t-1}\right)}^{2}+\frac{1\;-\;q}{2}P_{t-1}V\left(\theta_{t-1}\right)+\frac{1\;-\;q}{2}E{\left(\theta_{t-1}\right)}^{2}-2q(1\;-\;q)E\left(\theta_{t-1}\right)-E{\left(\theta_{t-1}\right)}^{2}\;\;=q(1\;-\;q)+\frac{1\;-\;q}{2}V\left(\theta_{t-1}\right)\left\{1+P_{t-1}\right\}+(1\;-\;q)E{\left(\theta_{t-1}\right)}^{2}-2q(1\;-\;q)E\left(\theta_{t-1}\right)-(1\;-\;q{)}^{2}E{\left(\theta_{t-1}\right)}^{2}\;\;=q(1\;-\;q)E{\left(\theta_{t-1}\right)}^{2}+q(1\;-\;q)\left\{1-2E\left(\theta_{t-1}\right)\right\}+\frac{1\;-\;q}{2}V\left(\theta_{t-1}\right)\left\{1+P_{t-1}\right\}$$

### Genetic variance

Let y=g+e, where y is the phenotypic value of an individual, g is the genotypic value, and e is random error. We assume additive effects such that $g=\sum_{i=1}^{m}\;\beta_{i}x_{i}$ where $\beta_{i}$ is the effect size of the $i^{\text{th}}$ biallelic locus and $x_{i}\in \{0,\;1,\;2\}$ is the number of copies of the trait-increasing allele. Then, the genetic variance $V_{g}$ is:

$$V_{g}=V\left(\sum_{i=1}^{m}\;\;\beta_{i}x_{i}\right)=\sum_{i=1}^{m}\;\beta_{i}^{2}V\left(x_{i}\right)+\sum_{j\neq i}\;\beta_{i}\beta_{j}C\left(x_{i},x_{j}\right)$$

We first derive $V\left(x_{i}\right)$ as a function of ancestry (θ) using the law of total variance:

$$V\left(x_{i}\right)=E\left\{V\left(x_{i}|\theta \right)\right\}+V\left\{E\left(x_{i}|\theta \right)\right\}$$

where θ represents global ancestry. Let $f_{i}^{A}$ and $f_{i}^{B}$ represent the allele frequency at the $i^{th}$ locus. Then,

$$E\{V(x_{i}|\theta )\}=E\{2\theta f_{i}^{A}(1-f_{i}^{A})\;+\;2(1-\theta )f_{i}^{B}(1-f_{i}^{B})+2\theta (1-\theta ){\left(f_{i}^{A}-f_{i}^{B}\right)}^{2}\}\;\;=2E(\theta )f_{i}^{A}(1-f_{i}^{A})+2\{1-E(\theta )\}f_{i}^{B}(1-f_{i}^{B})+2\{E(\theta )-E(\theta^{2})\}{\left(f_{i}^{A}-f_{i}^{B}\right)}^{2}\;\;=2E(\theta )f_{i}^{A}(1-f_{i}^{A})+2\{1-E(\theta )\}f_{i}^{B}(1-f_{i}^{B})+2\{E(\theta )-V(\theta )-E(\theta {)}^{2}\}{\left(f_{i}^{A}-f_{i}^{B}\right)}^{2}\;\;=2E(\theta )f_{i}^{A}(1-f_{i}^{A})+2\{1-E(\theta )\}f_{i}^{B}(1-f_{i}^{B})+2E(\theta )\{1-E(\theta )\}{\left(f_{i}^{A}-f_{i}^{B}\right)}^{2}\;\;-2V(\theta ){\left(f_{i}^{A}-f_{i}^{B}\right)}^{2}$$

$$V\left\{E\left(x_{i}|\theta \right)\right\}=V\left\{2\theta f_{\text{i}}^{\text{A}}+2(1-\theta )f_{\text{i}}^{\text{B}}\right\}\;\;\;\;\;\;\;\;\;\;\;\;\;\;\;\;\;\;\;\;\;\;\;\;=4\overset{2}{f_{\text{i}}^{\text{A}}}V(\theta )=4\overset{2}{f_{\text{i}}^{\text{B}}}V(1-\theta )+2\mathbb{C}\left\{2\theta f_{\text{i}}^{\text{A}},2(1-\theta )f_{\text{i}}^{\text{B}}\right\}\;\;\;\;\;\;\;\;\;\;\;\;\;\;\;\;\;\;\;\;\;\;\;\;=4\overset{2}{f_{\text{i}}^{\text{A}}}V(\theta )+4\overset{2}{f_{\text{i}}^{\text{B}}}V(1-\theta )-8f_{\text{i}}^{\text{A}}f_{\text{i}}^{\text{B}}V(\theta )$$

$$V\left(x_{i}\right)=2E(\theta )f_{i}^{A}(1-f_{i}^{A})+2\{1-E(\theta )\}f_{i}^{B}(1-f_{i}^{B})\;\;+2E(\theta )\{1-E(\theta )\}{\left(f_{i}^{A}-f_{i}^{B}\right)}^{2}+2V(\theta ){\left(f_{i}^{A}-f_{i}^{B}\right)}^{2}$$

Similarly, we derive $C\left(x_{i},x_{j}\right)$ using the law of total covariance:

$$C\left(x_{i},x_{j}\right)=E\left\{C\left(x_{i},x_{j}|\theta \right)\right\}+C\left\{E\left(x_{i}|\theta \right),E\left(x_{j}|\theta \right)\right\}\;\;=0+C\{2f_{i}^{A}\theta +2f_{i}^{B}(1-\theta ),2f_{j}^{A}\theta +2f_{j}^{B}(1-\theta )\}\;\;=C(2f_{i}^{A}\theta ,2f_{j}^{A}\theta )+C(2f_{i}^{A}\theta ,2f_{j}^{B}(1-\theta )+\;\;C(2f_{i}^{B}(1-\theta ),2f_{j}^{A}\theta )+C\left(2f_{i}^{B}(1-\theta ),2f_{j}^{B}(1-\theta )\right)\;\;=4V(\theta )(f_{i}^{A}-f_{i}^{B})(f_{j}^{A}-f_{j}^{B})$$

$E\left\{C\left(x_{i},x_{j}|\theta \right)\right\}=0$ because we assume that the loci are unlinked and therefore, $x_{i}$ and $x_{j}$ are conditionally independent. Putting this all together, we get the genetic variance in admixed populations as presented in the main text:

$$V_{g}\;=\sum_{i=1}^{m}\;\;\beta_{i}^{2}V\left(x_{i}\right)+\sum_{j\neq i}\;\;\beta_{i}\beta_{j}C\left(x_{i},x_{j}\right)\;\;=\sum_{i=1}^{m}\;\;\beta_{i}^{2}2E(\theta )f_{i}^{A}(1-f_{i}^{A})+\sum_{i=1}^{m}\;\;\beta_{i}^{2}2\{1-E(\theta )\}f_{i}^{B}\left(1-f_{i}^{B}\right)\;\;+\sum_{i=1}^{m}\;\;\beta_{i}^{2}2E(\theta )\{1-E(\theta )\}{\left(f_{i}^{A}-f_{i}^{B}\right)}^{2}+\;\;+\sum_{i=1}^{m}\;\;\beta_{i}^{2}2V(\theta ){\left(f_{i}^{A}-f_{i}^{B}\right)}^{2}]\;\;+\sum_{j\neq i}\;\;\beta_{i}\beta_{j}4V(\theta )(f_{i}^{A}-f_{i}^{B})(f_{j}^{A}-f_{j}^{B})$$

With two ‘unadmixed’ source populations with equal number of individuals, E(θ)=0.5 and V(θ)=E(θ){1-E(θ)}=0.25 and $V_{g}$ reduces to:

$$V_{g}\;=V(\sum_{i=1}^{m}\;\;\beta_{i}x_{i})=\sum_{i=1}^{m}\;\;\beta_{i}^{2}V\left(x_{i}\right)+\sum_{j\neq i}\;\;\beta_{i}\beta_{j}C\left(x_{i},x_{j}\right)\;\;=\sum_{i=1}^{m}\;\;\beta_{i}^{2}[f_{i}^{A}(1-f_{i}^{A})+f_{i}^{B}\left(1-f_{i}^{B}\right)]\;\;+\sum_{i=1}^{m}\;\;\beta_{i}^{2}{\left(f_{i}^{A}-f_{i}^{B}\right)}^{2}\;\;+\sum_{i\neq j}\;\;\beta_{i}\beta_{j}(f_{i}^{A}-f_{i}^{B})(f_{j}^{A}-f_{j}^{B})$$

### The effect of genotype scaling on ${\overset{\wedge }{sigma}}_{u}^{2}$

In the main text we showed that when we scale genotypes by $\sqrt{2f_{i}\left(1-f_{i}\right)}$ in calculating the GRM, we get biased GREML estimates even if the effects are uncorrelated. We can fix this issue by scaling with the square root of sample variance, $\sqrt{V\left(x_{i}\right)}$ (Fig. S3). We provide an explanation of this behavior using the Haseman-Elston (HE) regression estimator, which is asymptotically equivalent to the GREML estimator if effects are uncorrelated [61] but which, unlike GREML, has a closed-form solution. We assume random effects corresponding to the unscaled genotypes are $\beta_{i}∼𝒩(0,\frac{\sigma_{u}^{2}}{2mf_{i}\left(1-f_{i}\right)})$.

$$V_{g}=V\left(\sum_{i=1}^{m}\;\;\beta_{i}x_{i}\right)=\sum_{i=1}^{m}\;\beta_{i}^{2}V\left(x_{i}\right)$$

In expectation,

$$V\left(x_{i}\right)=2E(\theta )f_{i}^{A}(1-f_{i}^{A})+2\{1-E(\theta )\}f_{i}^{B}\left(1-f_{i}^{B}\right)+\;\;2E(\theta )\{1-E(\theta )\}{\left(f_{i}^{A}-f_{i}^{B}\right)}^{2}+2V(\theta ){\left(f_{i}^{A}-f_{i}^{B}\right)}^{2}\;\;=2f_{i}\left(1-f_{i}\right)+2V(\theta ){\left(f_{i}^{A}-f_{i}^{B}\right)}^{2}$$

And

$$V_{g}=\sum_{i=1}^{m}\;\;\beta_{i}^{2}\{2f_{i}\left(1-f_{i}\right)+2V(\theta ){\left(f_{i}^{A}-f_{i}^{B}\right)}^{2}\}\;\;=\sum_{i=1}^{m}\;\;\frac{\sigma_{u}^{2}}{2mf_{i}(1\;-\;f_{i})}\{2f_{i}\left(1\;-\;f_{i}\right)+2V(\theta ){\left(f_{i}^{A}-f_{i}^{B}\right)}^{2}\}\;\;=\frac{\sigma_{u}^{2}}{m}\sum_{i=1}^{m}\;\;\{1+V(\theta )\frac{{\left(f_{i}^{A}\;-\;f_{i}^{B}\right)}^{2}}{f_{i}(1\;-\;f_{i})}\}\;\;=\sigma_{u}^{2}+\underset{\begin{array}{c} \;\text{contribution}\;\text{of}\;\text{population}\;\text{structure}\;\\ \;\text{to}\;\text{the}\;\text{genic}\;\text{variance}\; \end{array}}{\underbrace{V(\theta )\frac{\sigma_{u}^{2}}{m}\sum_{i=1}^{m}\;\;\frac{{\left(f_{i}^{A}\;-\;f_{i}^{B}\right)}^{2}}{f_{i}(1\;-\;f_{i})}}}$$

Thus, the genetic variance can be decomposed into two terms, one that depends on the degree of population structure and one that does not.

The HE estimator of $V_{g}$ is based on the regression of products of (centered) phenotypes $y_{k}y_{l}$ for all pairs of individuals k≠l on the corresponding entries of the GRM (ψ) where $\psi_{kl}=\frac{\sum_{i=1}^{m}\;z_{ik}z_{il}}{m}$ and $z_{ik}$ is the centered and scaled genotype of individual k for locus i:

$${\overset{ˆ}{V}}_{g}\;=\frac{C\left(y_{k}y_{l},\;\psi_{kl}\right)}{V\left(\psi_{kl}\right)}\;\;=\frac{E\left(y_{k}y_{l}\psi_{kl}\right)\;-\;E\left(y_{k}y_{l}\right)E\left(\psi_{kl}\right)}{E\left(\psi_{kl}^{2}\right)\;-\;E{\left(\psi_{kl}\right)}^{2}}\;\;=\frac{E\left(y_{k}y_{l}\psi_{kl}\right)}{E\left(\psi_{kl}^{2}\right)}$$

With the standard scaling, $z_{ik}=\frac{x_{ik}-2f_{i}}{\sqrt{2f_{i}\left(1-f_{i}\right)}}$ and the corresponding effects are $\alpha_{i}∼𝒩\left(0,\sigma_{u}^{2}\right)$. In this case, the HE estimator is:

$${\overset{ˆ}{V}}_{g}\;=\frac{E\left(y_{k}y_{l}\psi_{kl}\right)}{E\left(\psi_{kl}^{2}\right)}=\frac{E\left(\sum_{i=1}^{m}\;\;\alpha_{i}z_{ik}\sum_{i=1}^{m}\;\;\alpha_{i}z_{il}\psi_{kl}\right)}{E\left(\psi_{kl}^{2}\right)}\;\;=\frac{E(\sum_{i=1}^{m}\;\;\alpha_{i}^{2}z_{ik}z_{il}\psi_{kl})}{E(\psi_{kl}^{2})}\;\;=\frac{E\left(\alpha_{i}^{2}\right)E(\sum_{i=1}^{m}\;\;z_{ik}z_{il}\psi_{kl})}{E\left(\psi_{kl}^{2}\right)}=\frac{\frac{\sigma_{u}^{2}}{m}E\left(m\psi_{kl}^{2}\right)}{E\left(\psi_{kl}^{2}\right)}\;\;=\frac{\sigma_{u}^{2}E\left(\psi_{kl}^{2}\right)}{E\left(\psi_{kl}^{2}\right)}=\sigma_{u}^{2}$$

Which is not equal to $V_{g}$ because it does not capture the contribution of population structure. Next, we consider the case where the genotypes are standardized instead by the sample variance, i.e., $z_{kl}=\frac{x_{ik}-2f_{i}}{\sqrt{V\left(x_{i}\right)}}$ such that $V\left(z_{i}\right)=1$. We can derive $E\left(\alpha_{i}^{2}\right)$ corresponding to this scaling by noting that the genetic variance remains the same:

$$\;\sum_{i=1}^{m}\;\;\beta_{i}^{2}V\left(x_{i}\right)=\sum_{i=1}^{m}\;\;\alpha_{i}^{2}V\left(z_{i}\right)\;mE\left(\alpha^{2}\right)=\sigma_{u}^{2}+V(\theta )\frac{\sigma_{u}^{2}}{m}\sum_{i=1}^{m}\;\;\frac{{\left(f_{i}^{A}\;-\;f_{i}^{B}\right)}^{2}}{f_{i}(1\;-\;f_{i})}\;E\left(\alpha^{2}\right)=\frac{\sigma_{u}^{2}}{m}+V(\theta )\frac{\sigma_{u}^{2}}{m^{2}}\sum_{i=1}^{m}\;\;\frac{{\left(f_{i}^{A}\;-\;f_{i}^{B}\right)}^{2}}{f_{i}(1\;-\;f_{i})}$$

Note that because the genotypes are scaled properly, $E\left(\psi_{kl}\right)=0$ and $V\left(\psi_{kl}\right)=1$. Then, the HE estimator becomes:

$${\overset{ˆ}{V}}_{g}\;=\frac{E\left(y_{k}y_{l}\psi_{kl}\right)}{E\left(\psi_{kl}^{2}\right)}\;\;=\frac{E\left(\alpha_{i}^{2}\right)E\left(\sum_{i=1}^{m}\;\;z_{ik}z_{il}\right)}{E\left(\psi_{kl}^{2}\right)}\;\;=\left(\frac{\sigma_{u}^{2}}{m}+V(\theta )\frac{\sigma_{u}^{2}}{m^{2}}\sum_{i=1}^{m}\;\;\frac{{\left(f_{i}^{A}\;-\;f_{i}^{B}\right)}^{2}}{f_{i}(1\;-\;f_{i})}\right)\frac{mE\left(\psi_{kl}^{2}\right)}{E\left(\psi_{kl}^{2}\right)}\;\;=\sigma_{u}^{2}+V(\theta )\frac{\sigma_{u}^{2}}{m}\sum_{i=1}^{m}\;\;\frac{{\left(f_{i}^{A}\;-\;f_{i}^{B}\right)}^{2}}{f_{i}(1\;-\;f_{i})}$$

Which provides an unbiased estimate of the genic variance.

### Effect size of local ancestry

We define local ancestry $\gamma_{i}\in \{0,\;1,\;2\}$ as the number of alleles at locus i that trace their ancestry to population A. Thus, the local ancestry at locus i in individual k is a Binomial random variable with $E\left(\gamma_{i,k}\right)=2\theta_{k}$. We define the ancestry value of an individual as the weighted sum of their local ancestry: $\sum_{i=1}^{m}\;\phi_{i}\gamma_{i}$ where $\phi_{i}=\beta_{i}(f_{i}^{B}-f_{i}^{A})$.

To show this, note that ϕ=E(y∣γ=1)-E(y∣γ=0) where $E(y|\gamma =1)=\int_{-\infty }^{\infty }\;yh(y|\gamma =1)$ and h is a density function. Our goal is to express ϕ in terms of β, which is equal to E(y∣x=1)-E(y∣x=0). Furthermore, $E(y|x=1)=\int_{-\infty }^{\infty }\;yh(y|x=1)$. We can express h(y∣γ) in terms of h(y∣x) as follows:

$$h(y|\gamma =1)\;=h(y|x=0)P(x=0|\gamma =1)+h(y|x=1)P(x=1|\gamma =1)+h(y|x=2)P(x=2|\gamma =1)\;\;=h(y|x=0)2(1-f^{A})(1-f^{A})+h(y|x=1)\{f^{A}\left(1-f^{B}\right)+f^{B}(1-f^{A})\}+h(y|x=2)2f^{A}f^{B}$$

$$E(y|\gamma =1)=\int_{-\infty }^{\infty }\;yh(y|\gamma =1)dy\;=(1-f^{A})(1-f^{B})\int_{-\infty }^{\infty }\;\;yh(y|x=0)dy\;\;+\{f^{A}(1-f^{B})+f^{B}(1-f^{A})\}\int_{-\infty }^{\infty }\;\;yh(y|x=1)dy\;\;+f^{A}f^{B}\int_{-\infty }^{\infty }\;\;yh(y|x=2)dy\;=(1-f^{A})\left(1-f^{B}\right)E(y|x=0)+\{f^{A}\left(1-f^{B}\right)+f^{B}(1-f^{A})\}E(y|x=1)+f^{A}f^{B}E(y|x=2)\;=0+\{f^{A}\left(1-f^{B}\right)+f^{B}(1-f^{A})\}\beta +f^{A}f^{B}2\beta \;=\beta f^{A}+\beta f^{B}$$

Similary, $E(y|\gamma =0)=2\beta f^{B}$ and $\phi =E(y|\gamma =1)-E(y|\gamma =0)=\beta (f^{B}-f^{A})$

### Genetic variance due to local ancestry

(1)
$$\begin{array}{l} V_{\gamma }=V\left(\sum_{i=1}^{m}\;\;\phi_{i}\gamma_{i}\right)\\ \;=\sum_{i=1}^{m}\;\;\phi_{i}^{2}V\left(\gamma_{i}\right)+\sum_{i=1}^{m}\;\;\sum_{j\neq i}\;\;\phi_{i}\phi_{j}C\left(\gamma_{i},\gamma_{j}\right) \end{array}$$

We use the law of total variance and covariance to derive $V\left(\gamma_{i}\right)$ and $C\left(\gamma_{i},\gamma_{j}\right)$:

$$V\left(\gamma_{i}\right)\;=E\left\{V\left(\gamma_{i}|\theta \right)\right\}+V\left\{E\left(\gamma_{i}|\theta \right)\right\}\;\;=E\left\{2\theta \left(1-\theta \right)\right\}+V\left(2\theta \right)\;\;=2E\left(\theta \right)-2E\left(\theta^{2}\right)+4V\left(\theta \right)\;\;=2E\left(\theta \right)-2V\left(\theta \right)-2E{\left(\theta \right)}^{2}+4V\left(\theta \right)\;\;=2E\left(\theta \right)\left\{1-E\left(\theta \right)\right\}+2V\left(\theta \right)$$

$$\mathbb{C}\left(\gamma_{i},\gamma_{j}\right)\;=E\left\{\mathbb{C}\left(\gamma_{i},\gamma_{j}|\theta \right)\right\}+\mathbb{C}\left\{E\left(\gamma_{i},\gamma_{j}|\theta \right)\right\}\;\;=0+\mathbb{C}\left(2\theta ,2\theta \right)=4V\left(\theta \right)$$

$$V_{\gamma }=2E\left(\theta \right)\left\{1-E\left(\theta \right)\right\}\sum_{i=1}^{m}\phi_{i}^{2}+2\;\text{V}\left(\theta \right)\sum_{i=1}^{m}\phi_{i}^{2}+4\;\text{V}\left(\theta \right)\sum_{i=1}^{m}\sum_{j\neq 1}\phi_{i}\phi_{j}\;=2E\left(\theta \right)\left\{1-E\left(\theta \right)\right\}\sum_{i=1}^{m}\beta_{i}^{2}{\left(f_{1}^{\text{B}}-f_{1}^{\text{A}}\right)}^{2}\;\;+2\;\text{V}\left(\theta \right)\sum_{i=1}^{m}\beta_{i}^{2}{\left(f_{1}^{\text{B}}-f_{1}^{\text{A}}\right)}^{2}+\;4\;\text{V}\left(\theta \right)\sum_{i=1}^{m}\sum_{j\neq i}\beta_{i}\beta_{j}\left(f_{1}^{\text{B}}-f_{1}^{\text{A}}\right)\left(f_{j}^{\text{B}}-f_{j}^{\text{A}}\right)$$
