# Interpreting SNP heritability in admixed populations

**DOI:** 10.1101/2023.08.04.551959

**Published:** 2023-11-07

**Authors:** Jinguo Huang, Saonli Basu, Mark D. Shriver, Arslan A. Zaidi

**Affiliations:** 1 Bioinformatics and Genomics, Huck Institutes of the Life Sciences, Pennsylvania State University; 2 Department of Anthropology, Pennsylvania State University; 3 Department of Biostatistics, University of Minnesota; 4 Department of Genetics, Cell Biology, and Development, University of Minnesota; 5 Institute of Health Informatics, University of Minnesota

## Abstract

SNP heritability ($h_{snp}^{2}$) is defined as the proportion of phenotypic variance explained by genotyped SNPs and is believed to be a lower bound of heritability ($h^{2}$), being equal to it if all causal variants are known. Despite the simple intuition behind $h_{snp}^{2}$, its interpretation and equivalence to $h^{2}$ is unclear, particularly in the presence of population structure and assortative mating. It is well known that population structure can lead to inflation in ${\hat{h}}_{snp}^{2}$ estimates. Here we use analytical theory and simulations to demonstrate that $h_{snp}^{2}$ estimated with genome-wide restricted maximum likelihood (GREML) can be biased in admixed populations, even in the absence of confounding and even if all causal variants are known. This is because admixture generates linkage disequilibrium (LD), which contributes to the genetic variance, and therefore to heritability. GREML implicitly assumes this component is zero, which may not be true, particularly for traits under divergent or stabilizing selection in the source populations, leading under- or over-estimates of $h_{snp}^{2}$ relative to $h^{2}$. For the same reason, GREML estimates of local ancestry heritability ($h_{\gamma }^{2}$) will also be biased. We describe the bias in ${\hat{h}}_{snp}^{2}$ and ${\hat{h}}_{\gamma }^{2}$ as a function of admixture history and the genetic architecture of the trait and discuss its implications for genome-wide association and polygenic prediction.

## Introduction

The ability to estimate (narrow-sense) heritability ($h^{2}$) from unrelated individuals was a major advance in genetics. Traditionally, $h^{2}$ was estimated from family-based studies in which the phenotypic resemblance between relatives could be modeled as a function of their expected genetic relatedness [1]. But this approach was limited to analysis of closely related individuals where pedigree information is available and the realized genetic relatedness is not too different from expectation [2]. With the advent of genome-wide association studies (GWAS), we hoped that many of the variants underlying this heritability would be uncovered. But when genome-wide significant SNPs explained a much smaller fraction of the phenotypic variance, it became important to explain the missing heritability – were family-based estimates inflated or were GWAS just underpowered, limited by variant discovery?

Yang et al. (2010) [3] made the key insight that one could estimate the portion of $h^{2}$ tagged by genotyped SNPs, regardless of whether or not they were genome-wide significant, by exploiting the subtle variation in the realized genetic relatedness among apparently unrelated individuals [3-5]. This quantity came to be known colloquially as ‘SNP heritability’ ($h_{snp}^{2}$) and it is believed to be equal to $h^{2}$ if all causal variants are included among genotyped SNPs [3]. Indeed, estimates of $h_{snp}^{2}$ explain a much larger fraction of trait heritability than GWAS SNPs [3], approaching family-based estimates of $h^{2}$ when whole genome sequence data are used [6]. This has made it clear that GWAS have yet to uncover more variants with increasing sample size. Now, $h_{snp}^{2}$ has become an important aspect of the design of genetic studies and is often used to define the power of variant discovery in GWAS and the upper limit of polygenic prediction accuracy.

Despite the utility and simple intuition of $h_{snp}^{2}$, there is much confusion about its interpretation and equivalence to $h^{2}$, particularly in the presence of population structure and assortative mating [7-12]. But much of the discussion of heritability in structured populations has focused on biases in ${\hat{h}}_{snp}^{2}$ – the estimator – due to confounding effects of shared environment and linkage disequilibrium (LD) with other variants [7, 9-11, 13]. There is comparatively little discussion, at least in human genetics, on the fact that LD due to population structure also contributes to genetic variance, and therefore, is a component of heritability [1] (but see [14-16] for a rigorous discussion). We think this is at least partly due to the fact that most studies are carried out in cohorts with primarily European ancestry, where the degree of population structure is minimal and large effects of LD can be ignored. However, that is not the case for diverse, multi-ethnic cohorts, which have historically been underrepresented in genetic studies, but thanks to a concerted effort in the field, are now becoming increasingly common [17-23]. The complex structure in these cohorts also brings unique methodological challenges and it is imperative that we understand whether existing methods, which have largely been evaluated in more homogeneous groups, generalize to more diverse cohorts.

Our goal in this paper is to study the behavior of ${\hat{h}}_{snp}^{2}$ in admixed populations. What is its interpretation in the ideal situation where causal variants are known? Is it an unbiased estimate of $h^{2}?$ To answer these questions, we derived a general expression for the genetic variance in admixed populations, decomposing it in terms of the contribution of population structure, which influences both the genotypic variance at individual loci and the LD across loci. We used extensive simulations, where the ground truth is known, to show that ${\hat{h}}_{snp}^{2}$ estimated with genome-wide restricted maximum likelihood (GREML) [3, 5] can be biased in admixed and other structured populations, even in the absence of confounding and when all causal variants are known. We explain this in terms of the discrepancy between the model assumed by GREML and the generative model from which the genetic architecture of the trait in the population may have been sampled. We describe the bias in ${\hat{h}}_{snp}^{2}$ as a function of admixture history and genetic architecture and discuss its implications for GWAS and polygenic prediction accuracy.

## Model

### Genetic architecture

We begin by describing a generative model for the phenotype. Let y=g+e, where y is the phenotypic value of an individual, g is the genotypic value, and e is random error. We assume additive effects such that $g=\sum_{i=1}^{m}\beta_{i}x_{i}$ where $\beta_{i}$ is the effect size of the $i^{th}$ biallelic locus and $x_{i}\in \{0,1,2\}$ is the number of copies of the trait-increasing allele. Importantly, the effect sizes are fixed quantities and differences in genetic values among individuals are due to random variation in genotypes. Note, that this is different from the model assumed by GREML where genotypes are fixed and effect sizes are random [14].

We denote the mean, variance, and covariance with E(.), V(.), and C(.), respectively, where the expectation is measured over random draws from the population rather than random realizations of the evolutionary process. We can express the additive genetic variance of a quantitative trait as follows:

$$V_{g}=V(\sum_{i=1}^{m}\beta_{i}x_{i})=\sum_{i=1}^{m}\beta_{i}^{2}V(x_{i})+\sum_{j\neq i}\beta_{i}\beta_{j}C(x_{i},x_{j})$$

Here the first term represents the contribution of individual loci (genic variance) and the second term is the contribution of linkage disequilibrium (LD contribution). We make the assumption that loci are unlinked and therefore, the LD contribution is entirely due to population structure. We describe the behavior of $V_{g}$ in a population that is a mixture of two previously isolated populations A and B that diverged from a common ancestor. To do this, we denote θ as the fraction of the genome of an individual with ancestry from population A. Thus, θ=1 if the individual is from population A, 0 if they are from population B, and θ∈(0,1) if they are admixed. Then, $V_{g}$ can be expressed in terms of ancestry as (Appendix):

(1.1) $$V_{g}=2\;E(\theta )\sum_{i=1}^{m}\beta_{i}^{2}f_{i}^{A}(1-f_{i}^{A})+2\{1-E(\theta )\}\sum_{i=1}^{m}\beta_{i}^{2}f_{i}^{B}(1-f_{i}^{B})$$

(1.2) $$+2\;E(\theta )\{1-E(\theta )\}\sum_{i=1}^{m}\beta_{i}^{2}{\left(f_{i}^{A}-f_{i}^{B}\right)}^{2}$$

(1.3) $$+2\;V(\theta )\sum_{i=1}^{m}\beta_{i}^{2}{\left(f_{i}^{A}-f_{i}^{B}\right)}^{2}$$

(1.4) $$+4\;V(\theta )\sum_{i\neq j}\beta_{i}\beta_{j}(f_{i}^{A}-f_{i}^{B})(f_{j}^{A}-f_{j}^{B})$$

where $f_{i}^{A}$ and $f_{i}^{B}$ are the allele frequencies in populations A and B, and E(θ) and V(θ) are the mean and variance of individual ancestry. The sum of the first three terms represents the genic variance and the last term represents the LD contribution.

### Demographic history

**Figure 1:**
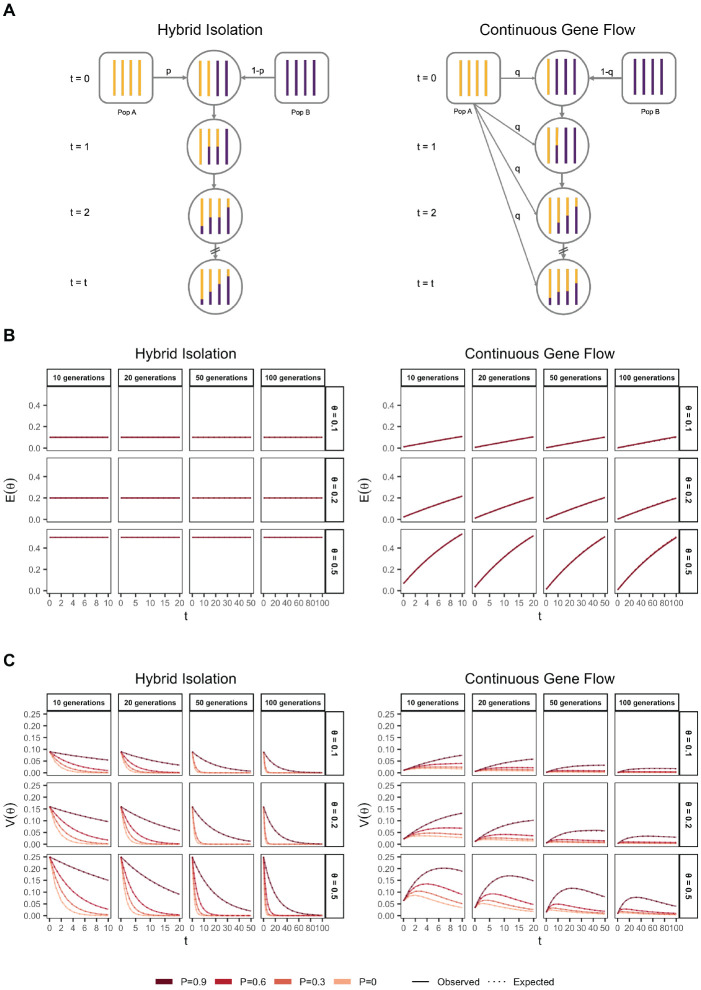
The behavior of mean and variance of individual ancestry as a function of admixture history. (A) Shows the demographic models under which simulations were carried out. Admixture might occur once (Hybrid Isolation, HI, left column) or continuously (Continuous Gene Flow, CGF, right column). (B) The mean individual ancestry, E(θ) remains constant over time in the HI model and increases in the CGF model with continued gene flow. (C) The variance in individual ancestry, V(θ) is maximum at t=0 in the HI model, decaying subsequently. V(θ) increases with gene flow in the CGF model and will subsequently decrease with time. P measures the strength of assortative mating, which slows the decay of V(θ). P=0.6 is missing for simulations run for 50 and 100 generations and θ∈{0.1,0.2} due to the difficulty in finding mate pairs (Methods).

From Eq. 1, it is clear that, conditional on the genetic architecture in the source populations (β, $f^{A}$, $f^{B}$), $V_{g}$ is a function of the mean, E(θ), and variance, V(θ), of individual ancestry in the admixed population. We consider two demographic models that affect E(θ) and V(θ) in qualitatively different ways. In the first model, the source populations meet once t generations ago (we refer to this as t=0) in proportions p and 1−p, after which there is no subsequent admixture (Fig. 1A). In the second model, there is continued gene flow in every generation from one of the source populations such that the mean overall amount of ancestry from population A is the same as in the first model (Fig. 1A). For brevity, we refer to these as the hybrid-isolation (HI) and continuous gene flow (CGF) models, respectively, following Pfaff *et al*. (2001) [24]. V(θ) is also affected by assortative mating based on ancestry (hereafter referred to as assortative mating for brevity) and we model this following Zaitlen *et al.* (2017) using a parameter P∈(0,1), which represents the correlation of the ancestry of individuals across mating pairs in the population[25].

Under these conditions, the behavior of E(θ) and V(θ) has been described previously [25, 26] (Fig. 1B and C). Briefly, in the HI model, E(θ) remains constant at p in the generations after admixture as there is no subsequent gene flow. V(θ) is at its maximum at t=0 when each individuals carries chromosomes either from population A or B, but not both. This genome-wide correlation in ancestry breaks down in subsequent generations as a function of mating, independent assortment, and recombination, leading to a decay in V(θ), the rate depending on the strength of assortative mating (Fig. 1C). In the CGF model, both E(θ) and V(θ) increase with time as new chromosomes are introduced from the source populations. But while E(θ) continues to increase monotonically, V(θ) will plateau and decrease due to the countervailing effects of independent assortment and recombination which redistribute ancestry in the population, reaching equilibrium at zero if there is no more gene flow and the population is mating randomly. V(θ) provides an intuitive and quantitative measure of the degree of population structure (along the axis of ancestry) in admixed populations.

## Results

### Genetic variance in admixed populations

**Figure 2:**
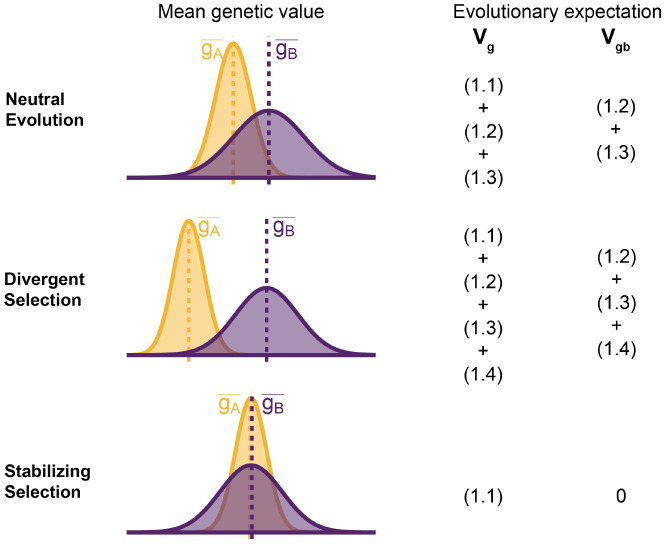
Decomposing genetic variance in a two-population system. The plot illustrates the expected distribution of genetic values in two populations under different selective pressures and the terms on the right list the total ($V_{g}$) and between-population genetic variance ($V_{gb}$) expected over the evolutionary process. For neutrally evolving traits (top row), we expect there to be an absolute difference in the mean genetic values ($|\overset{\bar{}}{g_{A}}-\overset{\bar{}}{g_{B}}|$) that is proportional to $F_{ST}$. For traits under divergent selection (middle), $|\overset{\bar{}}{g_{A}}-\overset{\bar{}}{g_{B}}|$ is expected to be greater than that expected under genetic drift. For traits under stabilizing selection, $|\overset{\bar{}}{g_{A}}-\overset{\bar{}}{g_{B}}|$ will be less than that expected under genetic drift, and zero in the extreme case.

To understand the expectation of genetic variance in admixed populations, it is first worth discussing its behavior in the source populations. In Eq. 1, the first term represents the within-population component ($V_{gw}$) and the last three terms altogether represent the component of genetic variance between populations A and B ($V_{gb}$). Note that $V_{gb}=\frac{{\left({\bar{g}}_{A}-{\bar{g}}_{B}\right)}^{2}}{2}$ is positive only if there is a difference in the mean genotypic values (Fig. 2). This variance increases with genetic divergence since the expected values of both ${\left(f_{i}^{A}-f_{i}^{B}\right)}^{2}$ and $\left(f_{i}^{A}-f_{i}^{B})(f_{j}^{A}-f_{j}^{B}\right)$ are functions of $F_{ST}$. But while ${\left(f_{i}^{A}-f_{i}^{B}\right)}^{2}$ is expected to increase monotonically with increasing divergence, $\left(f_{i}^{A}-f_{i}^{B})(f_{j}^{A}-f_{j}^{B}\right)$ is expected to be zero under neutrality because the direction of frequency change will be uncorrelated across loci. In this case, the LD contribution, i.e., (1.4), is expected to be zero and $V_{gb}=(1.1)+(1.2)+(1.3)$. However, this is true only in expectation over the evolutionary process and the realized LD contribution may be non-zero even for neutral traits.

For traits under selection, the LD contribution is expected to be greater or less than zero, depending on the type of selection. Under divergent selection, trait-increasing alleles will be systematically more frequent in one population over the other, inducing positive LD across loci [27, 28], increasing the LD contribution, i.e., term (1.4). Stabilizing selection, on the other hand, induces negative LD [29, 30]. In the extreme case, the mean genetic values of the two populations are exactly equal and $V_{gb}=(1.2)+(1.3+(1.4)=0$. For this to be true, (1.4) has to be negative and equal to (1.2) + (1.3), which are both positive, and the total genetic variance is reduced to the within-population variance, i.e., term (1.1) (Fig. 2). This is relevant because, as we show in the following sections, the behavior of the genetic variance in admixed populations depends on the magnitude of $V_{gb}$ between the source populations.

We illustrate this by tracking the genetic variance in admixed populations for two traits, both with the same mean $F_{ST}$ at causal loci but with different LD contributions (term 1.4): one where the LD contribution is positive (Trait 1) and the other where it is negative (Trait 2). Thus, traits 1 and 2 can be thought of as examples of phenotypes under divergent and stabilizing selection, respectively, and we refer to them as such from hereon. To simulate the genetic variance of such traits, we drew the allele frequencies ($f^{A}$ and $f^{B}$) in populations A and B for 1,000 causal loci with $F_{ST}∼0.2$ using the Balding-Nichols model [31]. We drew their effects (β) from $𝒩(0,\frac{1}{2mf(1-f)})$ where $\bar{f}$ is the mean allele frequency between the two populations, m is the number of loci. To simulate positive and negative LD, we permuted the effect signs across variants 100 times and selected the combinations that gave the most positive and negative LD contribution to represent the genetic architecture of traits that might be under directional (Trait 1) and stabilizing (Trait 2) selection, respectively (Methods). We simulated the genotypes of 10,000 individuals under the HI and CGF models for t∈{10,20,50,100} generations post-admixture and calculated genetic values for both traits using $g=\sum_{i=1}^{m}\beta_{i}x_{i}$, where m=1,000, (Method). The observed genetic variance at any time can then be calculated simply as the variance in genetic values, i.e. $V_{g}=V(g)$.

**Figure 3:**
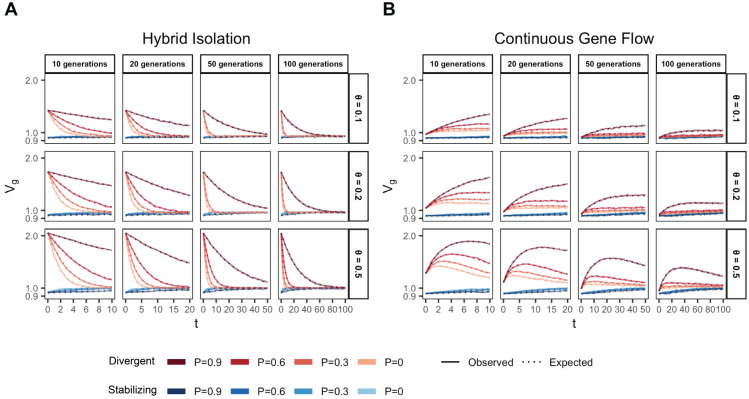
Genetic variance in admixed populations under the (A) HI and (B) CGF models. Dotted lines represent the expected genetic variance based on Eq. (1) and solid lines represent results of simulations averaged over ten replicates. Red and blue lines represent traits under divergent and stabilizing selection, respectively. P=0.6 is missing for simulations run for 50 and 100 generations and θ∈{0.1,0.2} due to the difficulty in finding mate pairs (Methods)

In the HI model, E(θ) does not change (Fig. 1B) so terms (1.1) and (1.2) are constant through time. Terms (1.3) and (1.4) decay towards zero as the variance in ancestry goes to zero and $V_{g}$ ultimately converges to (1.1) + (1.2) (Fig. 3). This equilibrium value is equal to the $E(V_{g}|\theta )$ (Appendix) and the rate of convergence depends on the strength of assortative mating, which slows the rate at which V(θ) decays. $V_{g}$ approaches equilibrium from a higher value for traits under divergent selection and lower value for traits under stabilizing selection because of positive and negative LD contributions, respectively, at t=0 (Fig. 3). In the CGF model, $V_{g}$ increases initially for both traits with increasing gene flow (Fig. 3). This might seem counter-intuitive at first because gene flow increases admixture LD, which leads to more negative values of the LD contribution for traits under stabilizing selection (Fig. S1). But this is outweighed by positive contributions from the genic variance – terms (1.1) + (1.2) + (1.3) – all of which initially increase with gene flow (Fig. S1). After a certain point, the increase in $V_{g}$ slows down as any increase in V(θ) due to gene flow is counterbalanced by recombination and independent assortment. Ultimately, $V_{g}$ will decrease if there is no more gene flow, reaching the same equilibrium value as in the HI model, i.e.,$E(V_{g}|\theta )=(1.1)+(1.2)$. Because the loci are unlinked, we refer to the sum (1.3) + (1.4) as the contribution of population structure.

### GREML estimation of $h_{snp}^{2}$

In their original paper, Yang *et al.* (2010) defined $h_{snp}^{2}$ as the variance explained by genotyped SNPs and not as heritability [3]. This is because $h^{2}$ is the genetic variance explained by causal variants, which are unknown. Genotyped SNPs may not overlap with or tag all causal variants and thus, $h_{snp}^{2}$ is understood to be a lower bound of $h^{2}$, both being equal if causal variants are known [3]. Our goal is to demonstrate that this may not be true in structured populations and quantify the bias in ${\hat{h}}_{snp}^{2}$, even in the ideal situation when causal variants are known.

We used GREML, implemented in GCTA [3, 5], to estimate the genetic variance for our simulated traits. GCTA assumes the following model: y=Zu+ϵ where Z is an n×m standardized genotype matrix such that the genotype of the $k^{th}$ individual at the $i^{th}$ locus is $z_{ik}=\frac{x_{ik}-2f_{i}}{\sqrt{{2f}_{i}(1-f_{i})}}$, $f_{i}$ being the allele frequency. The SNP effects corresponding to the scaled genotypes are assumed to be random and σ2 independent such that $u∼𝒩(0,I\frac{\sigma_{u}^{2}}{m})$ and $\epsilon ∼𝒩(0,I\sigma_{\epsilon }^{2})$ is random environmental error. Then, the m E phenotypic variance can be decomposed as:

$$V(y)=V(Zu)+V(e)\;\;=\frac{ZZ^{\prime }}{m}\;\sigma_{u}^{2}+\sigma_{\epsilon }^{2}$$

where $\frac{{\mathrm{ZZ}}^{\prime }}{m}$ is the genetic relationship matrix (GRM), the variance components $\sigma_{u}^{2}$ and $\sigma_{\epsilon }^{2}$ are estimated using restricted maximum likelihood, and ${\hat{h}}_{snp}^{2}$ is calculated as $\frac{{\hat{\sigma }}_{u}^{2}}{{\hat{\sigma }}_{u}^{2}+{\hat{\sigma }}_{\epsilon }^{2}}$. We are interested in asking whether ${\hat{\sigma }}_{u}^{2}$ is an unbiased estimate of $V_{g}$. To answer this, we constructed the GRM with causal variants and estimated ${\hat{\sigma }}_{u}^{2}$ using GCTA [3, 4].

**Figure 4:**
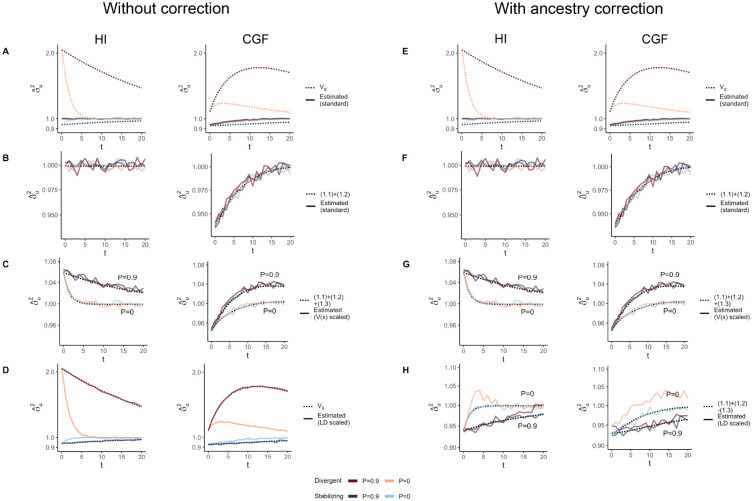
The behavior of GREML estimates of the genetic variance (${\hat{\sigma }}_{u}^{2}$) in admixed populations under the HI (left column) and CGF (right column) models either without (A-D) or with (E-H) individual ancestry as a fixed effect. The solid lines represent estimates from simulated data averaged across ten replicates with red and blue colors representing estimates for traits under divergent and stabilizing selection, respectively. P indicates the strength of assortative mating. The dotted lines either represent the expected variance in the population based on Eq. 1 (A & B) or the expected estimate for three different ways of scaling genotypes (B-D & F-H). (A-B & E-F) show the behavior of ${\hat{\sigma }}_{u}^{2}$ for the default scaling, (C, G) shows ${\hat{\sigma }}_{u}^{2}$ when the genotype at a locus is scaled by its sample variance (V(x) scaled), and (D, H) when it is scaled by the sample covariance (LD scaled).

GCTA under- and over-estimates the genetic variance in admixed populations for traits under divergent (Trait 1) and stabilizing selection (Trait 2), respectively, when there is population structure, i.e., when V(θ)>1 (Fig. 4A). One reason for this bias is that the GREML model assumes that the effects are independent, and therefore the LD contribution is zero. This, as discussed in the previous section, is not true for traits under divergent or stabilizing selection between the source populations, and only true for neutral traits in expectation. Because of this, ${\hat{\sigma }}_{u}^{2}$ does not capture the LD contribution, i.e. term (1.4) (Fig. 4A). But ${\hat{\sigma }}_{u}^{2}$ can be biased even if the LD contribution is zero if the genotypes are scaled with $\sqrt{{2f}_{i}(1-f_{i})}$ – the standard practice – where $f_{i}$ is the frequency of the allele in the population. This scaling assumes that $V(x_{i})=2f_{i}(1-f_{i})$, which is true only if the population were mating randomly. In an admixed population $V(x_{i})=2f_{i}(1-f_{i})+2\;V(\theta ){\left(f_{i}^{A}-f_{i}^{B}\right)}^{2}$, where $f_{i}$, $f_{i}^{A}$, and $f_{i}^{B}$ correspond to frequency in the admixed population, and source populations, A and B, respectively (Appendix). Alternatively, if the genotypes are scaled, $V(z_{i})=1+V(\theta )F_{st}^{\left(i\right)}$ where $F_{st}^{\left(i\right)}$ is the $F_{st}$ at the $i^{th}$ locus. We show that this assumption biases ${\hat{\sigma }}_{u}^{2}$ downwards by a factor of $2\;V(\theta ){\left(f_{i}^{A}-f_{i}^{B}\right)}^{2}$ (or $2\;V(\theta )F_{st}^{\left(i\right)}$ if genotypes are scaled) – term (1.3) (Fig. 4B, Appendix). Thus, with the standard scaling, ${\hat{\sigma }}_{u}^{2}$ gives a biased estimate in the presence of population structure, even of the genic variance.

The overall bias in ${\hat{\sigma }}_{u}^{2}$ is determined by the relative magnitude and direction of terms (1.3) and (1.4), both of which are functions of V(θ), and therefore, of the degree of structure in the population. The contribution of term (1.3) will be modest, even in highly structured populations (Fig. S1) and therefore, the overall bias is largely driven by the LD contribution. If there is no more gene flow, V(θ) will ultimately go to zero and $V_{g}$ will converge towards ${\hat{\sigma }}_{u}^{2}$. Thus, ${\hat{\sigma }}_{u}^{2}$ is more accurately interpreted u u as the genetic variance expected if the LD contribution were zero and if the population were mating randomly. In other words, $E({\hat{\sigma }}_{u}^{2})=(1.1)+(1.2)\neq V_{g}$ (Fig. 4B).

In principle, we can recover the missing components of $V_{g}$ by scaling the genotypes appropriately. For example, we can recover term (1.3) by scaling the genotype at each variant i by its sample variance, i.e., $z_{ik}=\frac{x_{ik}-2f_{i}}{\sqrt{V(x_{i})}}$ (Fig. 4C) (Appendix). We can also recover term (1.4) by scaling the genotypes with the covariance between SNPs, i.e., the LD matrix, as previously proposed [32, 33] (Methods). In matrix form, the ‘LD-scaled’ genotypes can be written as $Z=L^{-1}{\left(X-2P\right)}^{\prime }$ where P is an n×m matrix such that all elements of the $i^{th}$ column contain the frequency of the $i^{th}$ SNP and L is the (lower diagonal) square root matrix of the LD matrix Σ, i.e., $\Sigma ={\mathrm{LL}}^{\prime }$ [32]. GREML recovers the LD contribution under this scaling, resulting in unbiased estimates of $V_{g}$ for both traits (Fig. 4D).

In practice, however, the LD contribution may not be fully recoverable for two reasons. One, the LD-scaled GRM requires computing the inverse of Σ or L which may not exist, especially if the number of markers is greater than the sample size – the case for most human genetic studies. Second, it is common to include individual ancestry or principal components of the GRM as fixed effects in the model to correct for population stratification. This should also have the effect of removing the components of genetic variance along the ancestry axes, the residual variance being equal to E{V(g∣θ)}=(1.1)+(1.2)−(1.3) (Appendix). Indeed, this is what we observe in Fig. 4H. Thus, if ancestry is included as a fixed effect, we expect $V_{g}$ to be underestimated in the presence of population structure, regardless of genetic architecture.

### Local ancestry heritability

A related quantity of interest in admixed populations is local ancestry heritability ($h_{\gamma }^{2}$), which is defined as the proportion of phenotypic variance that can be explained by local ancestry. Zaitlen *et al*. (2014) [34] showed that this quantity is related to, and can be used to estimate, $h^{2}$ in admixed populations. The advantage of this approach is that local ancestry segments shared between individuals are identical by descent and are therefore, more likely to tag causal variants compared to array markers, allowing one to potentially capture the contributions of rare variants [34]. Here, we show that (i) the relationship between $h_{\gamma }^{2}$ and $h^{2}$ is not straightforward and (ii) ${\hat{h}}_{\gamma }^{2}$ may not be an unbiased estimate of local ancestry heritability under the random effects model for the same reasons that ${\hat{h}}_{snp}^{2}$ is not unbiased.

We define local ancestry $\gamma_{i}\in \{0,1,2\}$ as the number of alleles at locus i that trace their ancestry to population A. Thus, ancestry at the $i^{th}$ locus in individual k is a binomial random variable with $E(\gamma_{ik})={2\theta }_{k}$, $\theta_{k}$ being the ancestry of the $k^{th}$ individual. Similar to genetic value, the ‘ancestry value’ of an individual can be defined as $\sum_{i=1}^{m}\phi_{i}\gamma_{i}$, where $\phi_{i}=\beta_{i}(f_{i}^{A}-f_{i}^{B})$ is the effect size of local ancestry (Appendix). Then, the genetic variance due to local ancestry can be expressed as (Appendix):

$$V_{\gamma }=V\left(\sum_{i=1}^{m}\phi_{i}\gamma_{i}\right)=\sum_{i=1}^{m}\phi_{i}^{2}V(\gamma_{i})+\sum_{i=1}^{m}\sum_{j\neq i}\phi_{i}\phi_{j}C(\gamma_{i},\gamma_{j})\;\;=2\;E(\theta )\{1-E(\theta )\}\sum_{i=1}^{m}\phi_{i}^{2}+2\;V(\theta )=\sum_{i=1}^{m}\phi_{i}^{2}+4\;V(\theta )\sum_{i=1}^{m}\sum_{j\neq i}\phi_{i}\phi_{j}\;\;=2\;E(\theta )\{1-E(\theta )\}\sum_{i=1}^{m}\beta_{i}^{2}{\left(f_{i}^{A}-f_{i}^{B}\right)}^{2}\;\;+2\;V(\theta )\sum_{i=1}^{m}\beta_{i}^{2}{\left(f_{i}^{A}-f_{i}^{B}\right)}^{2}\;\;+4\;V(\theta )\sum_{i=1}^{m}\sum_{j\neq i}\beta_{i}\beta_{j}(f_{i}^{A}-f_{i}^{B})(f_{j}^{A}-f_{j}^{B})$$

and heritability explained by local ancestry is simply the ratio of $V_{\gamma }$ and the phenotypic variance. Note that $V_{\gamma }=(1.2)+(1.3)+(1.4)$ and therefore its behavior is similar to $V_{g}$ in that the terms (1.3) and (1.4) decay towards zero as V(θ)→0, and $V_{\gamma }$ converges to (1.2) (Fig. S2). Additionally, the dependence of $V_{\gamma }$ on both E(θ) and V(θ) precludes a straightforward derivation between local ancestry heritability and $h^{2}$.

GREML estimation of ${\hat{h}}_{\gamma }^{2}$ is similar to that of ${\hat{h}}_{snp}^{2}$, the key difference being that the former involves constructing the GRM using local ancestry instead of genotypes [34]. The following model is assumed: y=Wv+ξ where W is an n×m standardized local ancestry matrix, $v\;∼\;𝒩(0,I\frac{\sigma_{v}^{2}}{m})$ are local ancestry effects, and $\xi ∼𝒩(0,I\sigma_{\xi }^{2})$. Note that $\sigma_{\xi }^{2}$ captures both environmental noise as well as any genetic variance independent of local ancestry. The phenotypic variance is decomposed as $V(y)=V(Wv)+V(\xi )=\frac{{WW}^{\prime }}{m}\sigma_{v}^{2}+\sigma_{\xi }^{2}$ where $\frac{{WW}^{\prime }}{m}$ is the local ancestry GRM and $\sigma_{v}^{2}$ is the parameter of interest, which is believed to be equal to $V_{\gamma }$ – the genetic variance due to local ancestry.

**Figure 5:**
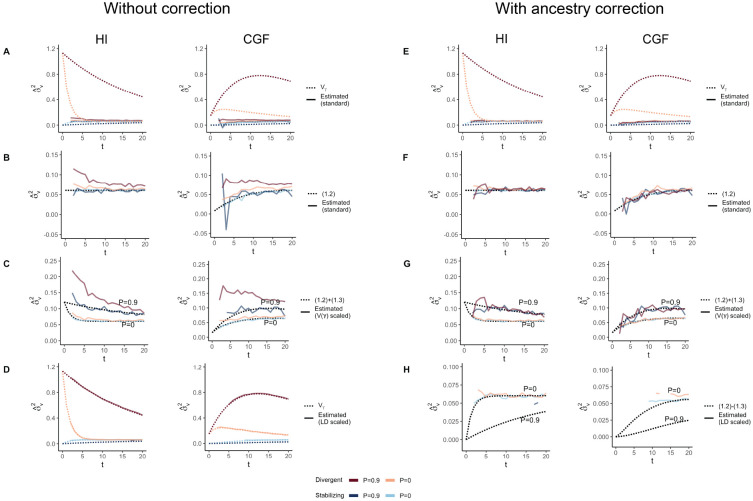
The behavior of GREML estimates of the variance due to local ancestry (${\hat{\sigma }}_{v}^{2}$) in admixed populations under the HI (left column) and CGF (right column) models either without (A-D) or with (E-H) individual ancestry included as a fixed effect. The solid lines represent estimates from simulated data averaged across ten replicates with red and blue colors representing estimates for traits under divergent and stabilizing selection, respectively. P indicates the strength of assortative mating. The dotted lines either represent the expected variance in the population (A & B) or the expected estimate for three different ways of scaling local ancestry (B-D & F-H). (A-B & E-F) show the behavior of ${\hat{\sigma }}_{v}^{2}$ for the default scaling, (C, G) shows ${\hat{\sigma }}_{v}^{2}$ when local ancestry is scaled by the sample variance, and (D, H) when it is scaled by the sample covariance. Some runs in (D & H) failed to converge as seen by the missing segments of the solid lines because the expected variance in such cases was too small.

We show that, in the presence of population structure, i.e., when V(θ)>0, ${\hat{\sigma }}_{v}^{2}$ is biased downwards relative to $V_{\gamma }$ for traits under divergent selection and upwards for traits under stabilizing selection because it does not capture the contribution of LD (Fig. 5A). But there is another source of bias in ${\hat{\sigma }}_{v}^{2}$, which tends to be inflated in the presence of population structure if individual ancestry is not included as a covariate, even with respect to the expectation of $V_{\gamma }$ under equilibrium (seen more clearly in Fig. 5B-C). We suspect this inflation is because of strong correlations between local ancestry – local ancestry disequilibrium – across loci that inflates ${\hat{\sigma }}_{v}^{2}$ in a way that is not adequately corrected even when all causal variants are included in the model [4, 10]. Scaling local ancestry by its covariance removes this bias and recovers the contribution of LD (Fig. 5D) presumably because this accounts for the correlation in genotypes across loci. Including individual ancestry as a fixed effect also corrects for the inflation in ${\hat{\sigma }}_{v}^{2}$ (Fig. 5E-H). But as with ${\hat{\sigma }}_{u}^{2}$, this practice will underestimate the genetic variance due to local ancestry in the presence of population structure because it removes the variance along the ancestry axis (Fig. 5E-H).

Based on the above, ${\hat{h}}_{\gamma }^{2}$ and corresponding estimates of $h^{2}$ are more accurately interpreted as the heritability due to local ancestry and heritability, respectively, expected in the population in the absence of gene flow and under random mating. We believe ${\hat{h}}_{\gamma }^{2}$ is still useful in that, because it should capture the effects of rare variants, it can be used to estimate the upper bound of ${\hat{h}}_{snp}^{2}$.

In a previous paper, we suggested that local ancestry heritability could potentially be used to estimate the genetic variance between populations [35]. Our results suggest this is not possible for two reasons. First, the GREML estimator of local ancestry heritability, as we show in this section is biased and does not capture the LD contribution. But even if we were able to recover the LD component, our decomposition shows that local ancestry is equal to the genetic variance between populations ($V_{gb}$) only when E(θ)=0.5 and V(θ)=E(θ){1−E(θ)}=0.25, which is only possible at t=0 in the HI model. After admixture, V(θ) decays and the equivalence between $V_{\gamma }$ and $V_{gb}$ is lost, making it impossible to estimate the latter from admixed populations, especially for traits under divergent or stabilizing selection, even if the environment is randomly distributed with respect to ancestry. We note that this conclusion was recently reached independently by Schraiber and Edge (2023) [36].

### How much does population structure contribute in practice?

In the previous sections, we showed theoretically that ${\hat{h}}_{snp}^{2}$ may be biased in admixed populations even if the causal variants are known and in the absence of confounding. Ultimately, whether or not this is true in practice is an empirical question, which is difficult to answer because the causal variants, their $F_{ST}$, and the correlation between their effect sizes are unknown. Here, we sought to answer a related question: to what extent does population structure contribute to the variance explained by GWAS SNPs among Americans with mixed African and European ancestry? To answer this, we calculated the total genetic variance explained in the 1000 Genomes [37] ASW (African Americans from the Southwest) using independent genome-wide significant SNPs for 26 quantitative traits from the GWAS catalog [38]. We decomposed the variance explained into the four components in Equation 1 using allele frequencies ($f^{A}$ and $f^{B}$) from the 1000 Genomes YRI (Yorubans from Ibadan Nigeria) and CEU (Northern Europeans from Utah), and the mean (E(θ)≈0.77) and variance (V(θ)≈0.02) of individual ancestry from ASW (Methods).

**Figure 6:**
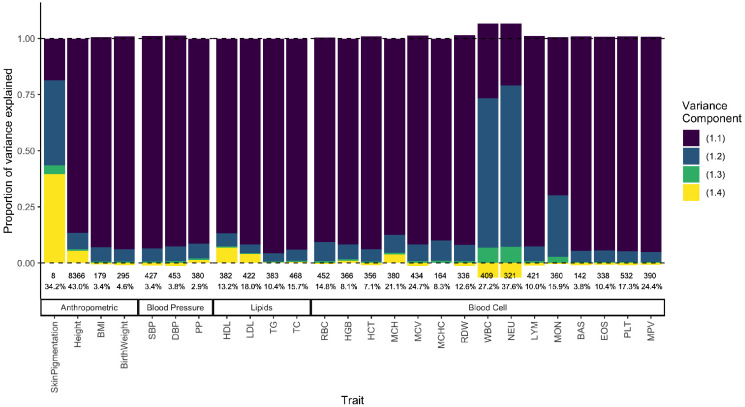
Decomposing the genetic variance explained by GWAS SNPs in the 1000 Genomes ASW (African Americans from Southwest). We calculated the four variance components listed in Eq. 1, their values shown on the y-axis as a fraction of the total variance explained (shown as percentage at the bottom). The number of variants used to calculate variance components for each trait is also shown at the bottom.

We show that for skin pigmentation – a trait under strong divergent selection – the LD contribution, i.e. term (1.4), is positive and accounts for ≈ 40 — 50% of the total variance explained. This is because of large allele frequency differences between Africans and Europeans that are correlated across skin pigmentation loci, consistent with strong polygenic selection favoring alleles for darker pigmentation in regions with high UV exposure and vice versa [35, 39-42]. But for most other traits, LD contributes relatively little, explaining a modest, but non-negligible proportion of the genetic variance in height, LDL and HDL cholestrol, mean corpuscular hemoglobin (MCH), neutrophil count (NEU), and white blood cell count (WBC) (Fig. 6). Because we selected independent associations for this exercise (Methods), the LD contribution is driven entirely due to population structure among ASW. The contribution of population structure to the genic variance, i.e., term (1.3) is also small even for traits like skin pigmentation and neutrophil count with large effect alleles that are highly diverged in frequency between Africans and Europeans [40, 41, 43-45]. Overall, this suggests that population structure contributes relatively little, as least to the variance explained by GWAS SNPs.

## Discussion

Despite the growing size of GWAS and discovery of thousands of variants for hundreds of traits [38], the heritability explained by GWAS SNPs remains a fraction of twin-based heritability estimates. Yang *et al*. (2010) introduced the concept of SNP heritability ($h_{snp}^{2}$) that does not depend on the discovery of causal variants but assumes that they are numerous and are more or less uniformly distributed across the genome (the infinitesimal model), their contributions to the genetic variance ‘tagged’ by genotyped SNPs [3]. $h_{snp}^{2}$ is now routinely estimated in most genomic studies and at least for some traits (e.g. height and BMI), these estimates now approach twin-based heritability [6]. But despite the widespread use of ${\hat{h}}_{snp}^{2}$, its interpretation remains unclear, particularly in the presence of admixture and population structure. It is generally accepted that ${\hat{h}}_{snp}^{2}$ can be biased in structured populations because of confounding effects of unobserved environmental factors and LD between causal variants [4, 7, 9-11, 46]. But ${\hat{h}}_{snp}^{2}$ may be biased even in the absence of confounding because of misspecification of the GREML model, i.e., if the model does not represent the genetic architecture from which the trait is sampled [14-16, 47, 48].

Under the standard GREML model, SNP effects are assumed to be uncorrelated and the total genetic variance can be represented as the sum of the variance explained by individual loci, i.e. the genic variance [14-16]. In admixed populations, there is substantial LD, which can contribute to the genetic variance, and can persist for a number of generations, despite recombination, due to continued gene flow and/or ancestry-based assortative mating. GREML does not capture this LD contribution [12, 15], and therefore, may lead to biased estimates of $h_{snp}^{2}$. The LD contribution can be negative for traits under stabilizing selection, and positive for traits under divergent selection between the source populations, leading to over- or under-estimates, respectively. Thus, GREML estimates of $h_{snp}^{2}$, assuming genotypes are scaled properly (see below), is better interpreted as the proportion of phenotypic variance explained by the *genic* variance. Estimates of local ancestry heritability (${\hat{h}}_{\gamma }^{2}$) [34, 49] should be interpreted similarly.

We show that ${\hat{h}}_{snp}^{2}$ can be biased even when the LD contribution is zero if the genotypes are scaled by $\sqrt{2f(1-f)}$ – the standard approach, which implicitly assumes a randomly mating population. In the presence of population structure, the variance in genotypes can be higher and ${\hat{h}}_{snp}^{2}$ does not capture this additional variance, which we show can be recovered by scaling genotypes by the SNP variance ($\sqrt{Var(x)}$). In principle, the LD contribution can also be recovered by scaling genotypes by the SNP covariance, i.e., the LD matrix, as previously suggested [32, 33]. But this approach is limited to situations where the sample size is much larger than the number of markers.

One limitation of this research is that it focuses on the GREML estimator of $h_{snp}^{2}$ because of its widespread use. Estimators of $h_{snp}^{2}$ can be broadly grouped into random- and fixed effect estimators based on how they treat SNP effects [50]. Fixed effect estimators make fewer distributional assumptions but they are not as widely used because they require conditional estimates of all variants – a high-dimensional problem where the number of markers is often far larger than the sample size [51]. This is one reason why random effect estimators, such as GREML, are popular – because they reduce the number of parameters that need to be estimated by assuming that the effects are drawn from some distribution where the variance is the only parameter of interest. Fixed effects estimators, in principle, should be able to capture the LD contribution but this is not obvious in practice since the simulations used to evaluate the accuracy of such estimators still assume uncorrelated effects [50-52]. Further research is needed to clarify the interpretation of the different estimators of $h_{snp}^{2}$ in structured populations under a range of genetic architectures.

Does the LD contribution to the genetic variance have practical implications? The answer to this depends on the context in which SNP heritability is used. ${\hat{h}}_{snp}^{2}$ can be useful in quantifying the power to detect variants in GWAS where the quantity of interest is the genic variance. But ${\hat{h}}_{snp}^{2}$ can lead to misleading conclusions if used to measure the extent to which genetic variation contributes to phenotypic variation, in predicting the response to selection, or in defining the upper limit of polygenic prediction accuracy [2] – applications where the LD contribution is important.

Ultimately, the discrepancy between ${\hat{h}}_{snp}^{2}$ and $h^{2}$ in practice is an empirical question, the answer to which depends on the degree of population structure (which we can measure) and the genetic architecture of the trait (which we do not know *a priori*). We show that for most traits, the contribution of population structure to the variance explained by GWAS SNPs is modest among African Americans. Thus, if we assume that the genetic architecture of GWAS SNPs represents that of all causal variants, then despite incorrect assumptions, the discrepancy between ${\hat{h}}_{snp}^{2}$ and $h^{2}$ should be fairly modest. But this assumption is unrealistic given that GWAS SNPs are common variants that in most cases cumulatively explain a fraction of trait heritability. What is the LD contribution of the rest of the genome, particularly rare variants? This is not obvious and will become clearer in the near future through large sequence-based studies [53]. While these are underway, theoretical studies are needed to understand how different selection regimes influence the LD patterns between causal variants – clearly an important aspect of the genetic architecture of complex traits.

## Methods

### Simulating genetic architecture

We first drew the allele frequency ($f^{0}$) of 1,000 biallelic causal loci in the ancestor of populations A and B from a uniform distribution, U(0.001,0.999). Then, we simulated their frequency in populations A and B ($f^{A}$ and $f^{B}$) under the Balding-Nichols model [31], such that $f^{A}$, $f^{B}∼Beta(\frac{f^{0}(1-F)}{F},\frac{\left(1-f^{0})(1-F\right)}{F})$ where F=0.2 is the inbreeding coefficient. We implemented this using code adapted from [54]. To avoid drawing extremely rare alleles, we continued to draw $f^{A}$ and $f^{B}$ until we had 1,000 loci with $f^{A}$, $f^{B}\in (0.01,0.99)$.

We generated the effect size (β) of each locus by sampling from $𝒩(0,\frac{1}{2m\bar{f}(1-\bar{f})})$, where m is the number of loci and $\bar{f}$ is the mean allele frequency across populations A and B. Thus, rare variants have larger effects than common variants and the total genetic variance sums to 1. Given these effects, we simulated two different traits, one with a large difference in means between populations A and B (Trait 1) and the other with roughly no difference (Trait 2). This was achieved by permuting the signs of the effects 100 times to get a distribution of $V_{gb}$ – the genetic variance between populations. This has the effect of varying the LD contribution without changing the $F_{ST}$ at causal loci. We selected the maximum and minimum of $V_{gb}$ to represent Traits 1 and 2.

### Simulating admixture

We simulated the genotypes, local ancestry, and phenotype for 10,000 admixed individuals per generation under the hybrid isolation (HI) and continuous gene flow (CGF) models by adapting the code from Zaitlen *et al*. (2017) [25]. We denote the ancestry of a randomly selected individual k with θ, the fraction of their genome from population A. At t=0 under the HI model, we set θ to 1 for individuals from population A and 0 if they were from population B such that E(θ)=p∈{0.1,0.2,0.5} with no further gene flow from either source population. In the CGF model, population B receives a constant amount q from population A in every generation starting at t=0. The mean overall proportion of ancestry in the population is kept the same as the HI model by setting $q=1-{\left(1-p\right)}^{\frac{1}{t}}$ where t is the number of generations of gene flow from A. In every generation, we simulated ancestry-based assortative mating by selecting mates such that the correlation between their ancestries is P∈{0,0.3,0.6,0.9} in every generation. We do this by repeatedly permuting individuals with respect to each other until P falls within ±0.01 of the desired value. It becomes difficult to meet this criterion when V(θ) is small (Fig.1C). To overcome this, we relaxed the threshold up to 0.04 for some conditions, i.e., when θ∈{0.1,0.2} and t≥50. We generated expected variance in individual ancestry using the expression in [25]. At time t since admixture, $V(\theta_{t})=V(\theta_{t-1})\frac{1+P}{2}$ under the HI model where P measures the strength of assortative mating, i.e, the correlation between the ancestry between individuals in a mating pair. Under the CGF model, $V(\theta_{t})=q(1-q)\;E{\left(\theta_{t-1}\right)}^{2}+q(1-q)\{1-2\;E(\theta_{t-1})\}+(1-q)\;V(\theta_{t-1})\frac{1+P}{2}$ (Appendix).

We sampled the local ancestry at each $i^{th}$ locus as $\gamma_{i}=\gamma_{if}+\gamma_{im}$ where $\gamma_{im}∼Bin(1,\theta_{m})$, $\gamma_{if}∼Bin(1,\theta_{f})$ and $\theta_{m}$ and $\theta_{f}$ represent the ancestry of the maternal and paternal chromosome, respectively. The global ancestry of the individual is then calculated as $\theta_{k}=\sum_{i=1}^{m}\frac{\gamma_{im}+\gamma_{if}}{2m}$, where m is the number of loci. We sample the genotypes $x_{im}$ and $x_{if}$ from a binomial distribution conditioning on local ancestry. For example, the genotype on the maternal chromosome is $x_{im}∼Bin(1,f_{i}^{A})$ if $\gamma_{im}=1$ and $x_{im}∼Bin(1,f_{i}^{B})$ if $\gamma_{im}=0$ where $f_{i}^{A}$ and $f_{i}^{B}$ represent the allele frequency in populations A and B, respectively. Then, the genotype can be obtained as the sum of the maternal and paternal genotypes: $x_{i}=x_{im}+x_{ip}$. We calculate the genetic value of each individual as $g=\sum_{i=1}^{m}\beta_{i}x_{i}$ and the genetic variance as V(g).

### Heritability estimation with GCTA

We used the --*reml* and --*reml-no-constrain* flags in GCTA [5] to estimate $\sigma_{u}^{2}$ and $\sigma_{v}^{2}$, the genetic variance due to genotypes and local ancestry, respectively. We could not run GCTA without noise in the genetic values so we simulated individual phenotypes with a heritability of $h^{2}=0.8$ by adding random noise $e∼𝒩(0,V_{g}\frac{1-h^{2}}{h^{2}})$. We computed three different GRMs, which correspond to different transformations of the genotypes: (i) standard, (i) Variance or V(x) scaled, and (ii) LD-scaled.

For the standard GRM, the genotypes at the $i^{th}$ SNP are standardized such that $z_{i}=\frac{x_{i}-2f_{i}}{\sqrt{2f_{i}(1-f_{i})}}$. For the variance scaled GRM, we computed $z_{i}=\frac{x_{i}-2f_{i}}{\sqrt{V(x_{i})}}$ where $V(x_{i})$ is the sample variance of the genotypes at the $i^{th}$ SNP. The LD-scaled GRM conceptually corresponds to standardizing the genotypes by the SNP covariance, rather than its variance. Let X represent the n×m *unstandardized* matrix of genotypes and P represent an n×m matrix where the $i^{th}$ column contains the allele frequency of that SNP. Let L be the lower diagonal ‘square root’ matrix of the m×m SNP covariance matrix Σ such that $\Sigma ={\mathrm{LL}}^{\prime }$. Then, the standardized genotypes are computed as $Z=L^{\prime }(X-2P)$ and the GRM becomes $(X-2P)\Sigma^{-1}{\left(X-2P\right)}^{\prime }$ [32]. Similarly, the three GRMs for local ancestry were computed by scaling local ancestry with (i) $\sqrt{2{\bar{\gamma }}_{i}(1-{\bar{\gamma }}_{i})}$ where we denote ${\bar{\gamma }}_{i}$ as the mean local ancestry at the $i^{th}$ SNP, or with the (ii) variance, or (iii) covariance of local ancestry, respectively.

We estimated $\sigma_{u}^{2}$ and $\sigma_{v}^{2}$ with and without individual ancestry as a fixed effect to correct for any confounding due to genetic stratification. This was done by using the --*qcovar* flag.

### Estimating variance explained by GWAS SNPs

To decompose the variance explained by GWAS SNPs in African Americans, we needed four quantities: (i) effect sizes of GWAS SNPs, (ii) their allele frequencies in Africans and Europeans, and (iii) the mean and variance of global ancestry in African Americans (Equation 1).

We retrieved the summary statistics of 26 traits from GWAS catalog [38]. Full list of traits and the source papers [42, 55-62] are listed in Table S1. To maximize the number of variants discovered, we chose summary statistics from studies that were conducted in both European and multi-ancestry samples and that reported the following information: effect allele, effect size, p-value, and genomic position. For birth weight, we downloaded the data from the Early Growth Genetics (EGG) consortium website [59] since the version reported on the GWAS catalog is incomplete. For skin pigmentation, we chose summary statistics from the UKB [63] released by the Neale Lab (http://www.nealelab.is/uk-biobank) and processed by Ju and Mathieson [42] to represent effect sizes estimated among individuals of European ancestry. We also selected summary statistics from Lona-Durazo *et al.* (2019) where effect sizes were meta-analyzed across four admixed cohorts [55]. Lona-Durazo *et al.* provide summary statistics separately with and without conditioning on rs1426654 and rs35397 – two large effect variants in *SCL24A5* and *SLC45A2*. We used the ‘conditioned’ effect sizes and added in the effects of rs1426654 and rs35397 to estimate genetic variance.

We selected independent hits for each trait by pruning and thresholding with PLINK v1.90b6.21 [64] in two steps as in Ju *et al.* (2020) [42]. We used the genotype data of GBR from the 1000 genome project [37] as the LD reference panel. We kept only SNPs (indels were removed) that passed the genome-wide significant threshold (--*clump-p1 5e-8*) with a pairwise LD cutoff of 0.05 (--*clump-r2 0.05*) and a physical distance threshold of 250Kb (--*clump-kb 250*) for clumping. Second, we applied a second round of clumping (--*clump-kb 100*) to remove SNPs within 100kb.

When GWAS was carried out separately in different ancestry cohorts in the same study, we used inverse-variance weighting to meta-analyze effect sizes for variants that were genome-wide significant (p-value < 5 × 10^−8^) in at least one cohort. This allowed us to maximize the discovery of variants such as the Duffy null allele that are absent among individuals of European ancestry but polymorphic in other populations [45].

We used allele frequencies from the 1000 Genomes CEU and YRI to represent the allele frequencies of GWAS SNPs in Europeans and Africans, respectively, making sure that the alleles reported in the summary statistics matched the alleles reported in the 1000 Genomes. We estimated the global ancestry of ASW individuals (N = 74) with CEU and YRI individuals from 1000 genome (phase 3) using ADMIXTURE 1.3.0 [65] with k=2 and used it to calculate the mean (proportion of African ancestry = 0.767) and variance (0.018) of global ancestry in ASW. With the effect sizes, allele frequencies, and the mean and variance in ancestry, we calculated the four components of genetic variance using Equation 1 and expressed them as a fraction of the total genetic variance.

Initially, the multi-ancestry summary statistics for a few traits (NEU, WBC, MON, MCH, BAS) yielded values > 1 for the proportion of variance explained. This is likely because, despite LD pruning, some of the variants in the model are not independent and tag large effect variants under divergent selection such as the Duffy null allele, leading to an inflated contribution of LD. We checked this by calculating the pairwise contribution , i.e., $\beta_{i}\beta_{j}(f_{i}^{A}-f_{i}^{B})(f_{j}^{A}-f_{j}^{B})$, of all SNPs in the model and show long-range positive LD between variants on chromosome 1 for NEU, WBC, and MON, especially with the Duffy null allele (Fig. S3A-C). A similar pattern was observed on chromosome 16 for MCH, confirming our suspicion. This also suggests that for certain traits, pruning and thresholding approaches are not guaranteed to yield independent hits. To get around this problem, we retained only one association with the lowest p-value, each from chromosome 1 (rs2814778 for NEU, WBC, and MON) and chromosome 16 (rs13331259 for MCH) (Fig. S3D). For BAS, we observed that the variance explained was driven by a rare variant (rs188411703, MAF = 0.0024) of large effect (β=−2.27). We believe this effect estimate to be inflated and therefore, we removed it from our calculation.

As a sanity check, we independently estimated the genetic variance as the variance in polygenic scores, calculated using --*score* flag in PLINK, [64] in ASW individuals. We compared the first estimate of the genetic variance to the second (Fig. S4) to confirm two things: (i) the allele frequencies, and mean and variance in ancestry are estimated correctly, and (ii) the variants are more or less independent in that they do not absorb the effects of other variants in the model. We show that the two estimates of the genetic variance are strongly correlated (r∼0.85, Fig. S4).

## Supplementary Material

Supplement 1

## Appendix

### 1 Variance in ancestry

We denote variance and covariance with V(.) and C(.) and used the expressions in [25] to generate the expected value for the variance in ancestry, i.e., V(θ). This is straightforward for the HI model, where at time $t\;V(\theta_{t})=V(\theta_{t-1})\frac{\left(1+P_{t-1}\right)}{2}$. $P_{t}=Cor(\theta_{m},\theta_{f})$ measures the strength of assortative mating, i.e, the correlation between the ancestry across mating pairs ($\theta_{m}$, $\theta_{f}$) at time t. For simplicity, we assumed this to be constant in every generation, i.e. $P_{t}=P_{t-1}=P$ following [25]. Since our notation slightly differs from [25], we re-derived the expression for $V(\theta_{t})$ for the CGF model where population B receives a constant amount q of gene flow from population A in every generation. Note, that $E(\theta_{t})=q+(1-q)\;E(\theta_{t-1})$. Then,

$$V(\theta_{t})={E(\theta_{t}^{2})-E(\theta_{t})}^{2}\;\;=q+(1-q)\;E\left[\left(\frac{\theta_{t-1}^{m}+\theta_{t-1}^{f}}{2}\right)\left(\frac{\theta_{t-1}^{m}+\theta_{t-1}^{f}}{2}\right)\right]-{\left\{q+\left(1-q\right)\;E(\theta_{t-1})\right\}}^{2}\;\;=q+\frac{\left(1-q\right)}{4}\{2\;E(\theta_{t-1}^{2})+2\;E(\theta_{t-1}^{m}\theta_{t-1}^{f})\}-\{q^{2}+2q(1-q)\;E(\theta_{t-1})+{\left(1-q\right)}^{2}\;{E(\theta_{t-1})}^{2}\}\;\;=q+\frac{1-q}{2}\;E(\theta_{t-1}^{2})+\frac{1-q}{2}\;E(\theta_{t-1}^{m}\theta_{t-1}^{f})-q^{2}-2q(1-q)\;E(\theta_{t-1})-{\left(1-q\right)}^{2}\;{E(\theta_{t-1})}^{2}\;\;=q\left(1-q\right)+\frac{1-q}{2}\{V(\theta_{t-1})+{E(\theta_{t-1})}^{2}\}+\frac{1-q}{2}\{C(\theta_{t-1}^{m},\theta_{t-1}^{f})+{E(\theta_{t-1})}^{2}\}-2q(1-q)\;E(\theta_{t-1})-{E(\theta_{t-1})}^{2}\;\;=q\left(1-q\right)+\frac{1-q}{2}\;V(\theta_{t-1})+\frac{1-q}{2}\;{E(\theta_{t-1})}^{2}+\frac{1-q}{2}P_{t-1}\;V(\theta_{t-1})+\frac{1-q}{2}\;{E(\theta_{t-1})}^{2}-2q(1-q)\;E(\theta_{t-1})-{E(\theta_{t-1})}^{2}\;\;=q\left(1-q\right)+\frac{1-q}{2}\;V(\theta_{t-1})\{1+P_{t-1}\}+(1-q)\;{E(\theta_{t-1})}^{2}-2q(1-q)\;E(\theta_{t-1})-{\left(1-q\right)}^{2}\;{E(\theta_{t-1})}^{2}\;\;=q(1-q)\;{E(\theta_{t-1})}^{2}+q(1-q)\{1-2\;E(\theta_{t-1})\}+\frac{1-q}{2}\;V(\theta_{t-1})\{1+P_{t-1}\}$$

### 2 Genetic variance - $V_{g}$

Let y=g+e, where y is the phenotypic value of an individual, g is the genotypic value, and e is random error. We assume additive effects such that $g=\sum_{i=1}^{m}\beta_{i}x_{i}$ where $\beta_{i}$ is the effect size of the $i^{th}$ biallelic locus and $x_{i}\in \{0,1,2\}$ is the number of copies of the trait-increasing allele. Then, the genetic variance $V_{g}$ is:

$$V_{g}=V(\sum_{i=1}^{m}\beta_{i}x_{i})=\sum_{i=1}^{m}\beta_{i}^{2}V(x_{i})+\sum_{j\neq i}\beta_{i}\beta_{j}C(x_{i},x_{j})$$

In the following sections, we decompose $V(x_{i})$ and $C(x_{i},x_{j})$ further as functions of ancestry.

#### 2.1 $V(x_{i})$

We first derive $V(x_{i})$ as a function of ancestry (θ) using the law of total variance:

$$V(x_{i})=\underset{\theta }{E}\{V(x_{i}|\theta )\}+V\{\underset{\theta }{E}(x_{i}|\theta )\}$$

where $E_{\theta }$ represents the expectation taken over θ.

##### 2.1.1 $E_{\theta }\{V(x_{i}|\theta )\}$

We derive $V(x_{i}|\theta )$ by further conditioning on the local ancestry at each locus.

$$V(x_{i}|\theta )=\underset{\gamma }{E}\{V(x_{i}|\gamma ,\theta )\}+V\{\underset{\gamma }{E}(x_{i}|\gamma ,\theta )\}$$

where $E_{\gamma }$ represents expectation taken over local ancestry. Since we are interested in the variance at a single locus, we will ignore the subscript i and denote the frequency of the trait-increasing allele in populations A and B with $f^{A}$ and $f^{B}$, respectively.

$$\underset{\gamma }{E}\{V(x_{i}|\gamma ,\theta )\}=V(x_{i}|\gamma =0,\theta )\;P(\gamma =0|\theta )+V(x_{i}|\gamma =1,\theta )\;P(\gamma =1|\theta )+V(x_{i}|\gamma =2,\theta )\;P(\gamma =2|\theta )\;\;=2\;f^{B}(1-f^{B}){\left(1-\theta \right)}^{2}+\{f^{A}(1-f^{A})+f^{B}(1-f^{B})\}2\theta (1-\theta )+2f^{A}(1-f^{A})\theta^{2}\;\;=(2\;f^{B}-{2f}^{A^{2}})(1-2\theta +\theta^{2})+(f^{A}-f^{A^{2}}+f^{B}-f^{B^{2}})(2\theta -{2\theta }^{2})+(2f^{A}-{2f}^{A^{2}})\theta^{2}\;\;=2\;f^{B}-2\theta \;f^{B}-{2f}^{B^{2}}+2\theta f^{B^{2}}+2\theta f^{A}-{2\theta f}^{A^{2}}\;\;=2\;f^{B}(1-\theta )-{2f}^{B^{2}}(1-\theta )+2\theta f^{A}(1-f^{A})\;\;=2\;f^{B}(1-f^{B})(1-\theta )+2\theta \;f^{A}(1-f^{A})$$

To derive $V\{E_{\gamma }(x|\gamma ,\theta )\}$, note that

$$\underset{\gamma }{E}(x|\gamma ,\theta )=\underset{\gamma }{E}\{E(x|\theta )\}\;\;=E(x|\gamma =0,\theta )\;P(\gamma =0|\theta )+E(x|\gamma =1,\theta )\;P(\gamma =1|\theta )+E(x|\gamma =2,\theta )\;P(\gamma =2|\theta )\;\;=2\theta \;f^{A}+2(1-\theta )\;f^{B}$$

And,

$$V\{\underset{\gamma }{E}(x|\gamma ,\theta )\}={\left[E(x|\gamma =0,\theta )-E(x|\theta )\right]}^{2}\;P(\gamma =0|\theta )\;\;+{\left[E(x|\gamma =1,\theta )-E(x|\theta )\right]}^{2}\;P(\gamma =1|\theta )\;\;+{\left[E(x|\gamma =2,\theta )-E(x|\theta )\right]}^{2}\;P(\gamma =2|\theta )\;\;=\theta^{2}\;{\left[2\;f^{A}-\{2\theta \;f^{A}+2(1-\theta )\;f^{B}\}\right]}^{2}\;\;+2\theta (1-\theta )\;{\left[f^{A}+f^{B}-\{2\theta \;f^{A}+2(1-\theta )\;f^{B}\}\right]}^{2}\;\;=+{\left(1-\theta \right)}^{2}{\left[2\;f^{B}-\{2\theta \;f^{A}+2(1-\theta )\;f^{B}\}\right]}^{2}\;\;=2\theta (1-\theta ){\left(f^{A}-f^{B}\right)}^{2}$$

Putting this together,

$$\underset{\theta }{E}\{V(x_{i}|\theta )\}=\underset{\theta }{E}\{2\;f^{B}(1-f^{B})(1-\theta )+2\theta f^{A}(1-f^{A})-2\theta (1-\theta ){\left(f^{A}-f^{B}\right)}^{2}\}\;\;=2f^{B}(1-f^{B})\{1-\underset{\theta }{E}(\theta )\}+2\underset{\theta }{E}(\theta )\;f^{A}(1-f^{A})+2\underset{\theta }{E}(\theta -\theta^{2}){\left(f^{A}-f^{B}\right)}^{2}\;\;=2f^{B}(1-f^{B})\{1-\underset{\theta }{E}(\theta )\}+2\underset{\theta }{E}(\theta )\;f^{A}(1-f^{A})+2\{\underset{\theta }{E}(\theta )-\underset{\theta }{E}(\theta^{2})\}{\left(f^{A}-f^{B}\right)}^{2}\;\;=2f^{B}(1-f^{B})\{1-\underset{\theta }{E}(\theta )\}+2\underset{\theta }{E}(\theta )\;f^{A}(1-f^{A})+2\{\underset{\theta }{E}(\theta )-V(\theta )-\underset{\theta }{E}{\left(\theta \right)}^{2}\}{\left(f^{A}-f^{B}\right)}^{2}\;\;=2f^{B}(1-f^{B})\{1-\underset{\theta }{E}(\theta )\}+2\underset{\theta }{E}(\theta )\;f^{A}(1-f^{A})+2\underset{\theta }{E}(\theta )(1-\underset{\theta }{E}(\theta )){\left(f^{A}-f^{B}\right)}^{2}-2\;V(\theta ){\left(f^{A}-f^{B}\right)}^{2}$$

##### 2.1.2 $V\{E_{\theta }(x_{i}|\theta )\}$

Recall from the previous section that $E_{\theta }(x_{i}|\theta )=2\theta \;f^{A}+2(1-\theta )\;f^{B}$. Then,

$$V\{\underset{\theta }{E}(x_{i}|\theta )\}=V\{2\theta f^{A}+2(1-\theta )f^{B}\}\;\;=4\;V(\theta )f^{A^{2}}+4\;V(1-\theta )f^{B^{2}}+2\;C(2\theta \;f^{A},2(1-\theta \;f^{B})\;\;=4\;V(\theta )f^{A^{2}}+4\;V(1-\theta )f^{B^{2}}-8f^{A}f^{B}V(\theta )\;\;=4\;V(\theta ){\left(f^{A}-f^{B}\right)}^{2}$$

We are now ready to express $V(x_{i})$:

$$V(x_{i})=2f^{B}(1-f^{B})\{1-\underset{\theta }{E}(\theta )\}+2\underset{\theta }{E}(\theta )\;f^{A}(1-f^{A})+2\underset{\theta }{E}(\theta )(1-\underset{\theta }{E}(\theta )){\left(f^{A}-f^{B}\right)}^{2}\;\;-2\;V(\theta ){\left(f^{A}-f^{B}\right)}^{2}+4\;V(\theta ){\left(f^{A}-f^{B}\right)}^{2}\;\;=2\underset{\theta }{E}(\theta )\;f_{i}^{A}(1-f_{i}^{A})+2\{1-\underset{\theta }{E}(\theta )\}\;f_{i}^{B}(1-f_{i}^{B})\;\;+2\;\underset{\theta }{E}(\theta )\{1-\underset{\theta }{E}(\theta )\}{\left(f_{i}^{A}-f_{i}^{B}\right)}^{2}-2\;V(\theta ){\left(f_{i}^{A}-f_{i}^{B}\right)}^{2}$$

#### 2.2 $C(x_{i},x_{j})$

We can derive $C(x_{i},x_{j})$ using the law of total covariance:

$$C(x_{i},x_{j})=\underset{\theta }{E}\{C(x_{i},x_{j}|\theta )\}+C\{\underset{\theta }{E}(x_{i}|\theta ),\underset{\theta }{E}(x_{j}|\theta )\}\;\;=0+C\{2f_{i}^{A}\;\theta +2\;f_{i}^{B}(1-\theta ),2f_{j}^{A}\;\theta +2\;f_{j}^{B}(1-\theta )\}\;\;=C(2f_{i}^{A}\;\theta ,2\;f_{j}^{A}\;\theta )+C(2f_{i}^{A}\;\theta ,2\;f_{j}^{B}(1-\theta )+\;\;C(2f_{i}^{B}(1-\theta ),2\;f_{j}^{A}\;\theta )+C(2f_{i}^{B}(1-\theta ),2\;f_{j}^{B}(1-\theta ))\;\;=4\;V(\theta )(f_{i}^{A}-f_{i}^{B})(f_{j}^{A}-f_{j}^{B})$$

$E_{\theta }\{C(x_{i},x_{j}|\theta )\}=0$ because we assume that the loci are unlinked and therefore, $x_{i}$ and $x_{j}$ are conditionally independent. Putting this all together, we get the genetic variance in admixed populations as presented in the main text:

$$V_{g}=\sum_{i=1}^{m}\beta_{i}^{2}V(x_{i})+\sum_{j\neq i}\beta_{i}\beta_{j}C(x_{i},x_{j})\;\;=\sum_{i=1}^{m}\beta_{i}^{2}2\underset{\theta }{E}(\theta )\;f_{i}^{A}(1-f_{i}^{A})+\sum_{i=1}^{m}\beta_{i}^{2}2\{1-\underset{\theta }{E}(\theta )\}\;f_{i}^{B}(1-f_{i}^{B})\;\;+\sum_{i=1}^{m}\beta_{i}^{2}2\underset{\theta }{E}(\theta )\{1-\underset{\theta }{E}(\theta )\}{\left(f_{i}^{A}-f_{i}^{B}\right)}^{2}+\;\;+\sum_{i=1}^{m}\beta_{i}^{2}2\;V(\theta ){\left(f_{i}^{A}-f_{i}^{B}\right)}^{2}]\;\;+\sum_{j\neq i}\beta_{i}\beta_{j}4\;V(\theta )(f_{i}^{A}-f_{i}^{B})(f_{j}^{A}-f_{j}^{B})$$

The only difference being that in the main text we use E instead of $E_{\theta }$ for simplicity. With two ‘unadmixed’ source populations with equal number of individuals, E(θ)=0.5 and V(θ)=E(θ){1−E(θ)}=0.25 and $V_{g}$ reduces to:

$$V_{g}=V\left(\sum_{i=1}^{m}\beta_{i}x_{i}\right)=\sum_{i=1}^{m}\beta_{i}^{2}V(x_{i})+\sum_{j\neq i}\beta_{i}\beta_{j}C(x_{i},x_{j})\;\;=\sum_{i=1}^{m}\beta_{i}^{2}\;[f_{i}^{A}(1-f_{i}^{A})+f_{i}^{B}(1-f_{i}^{B})]\;\;+\sum_{i=1}^{m}\beta_{i}^{2}{\left(f_{i}^{A}-f_{i}^{B}\right)}^{2}\;\;+\sum_{i\neq j}\beta_{i}\beta_{j}(f_{i}^{A}-f_{i}^{B})(f_{j}^{A}-f_{j}^{B})$$

### 3 The effect of genotype scaling on ${\hat{\sigma }}_{u}^{2}$

In the main text we showed that when we scale genotypes by $\sqrt{2f_{i}(1-f_{i})}$ in calculating the GRM, we get biased GREML estimates even if the effects are uncorrelated. We can fix this issue by scaling with the square root of sample variance, $\sqrt{V(x_{i})}$. We provide an explanation of this behavior using the Haseman-Elston (HE) regression estimator, which is asymptotically equivalent to the GREML estimator if effects are uncorrelated [68] but which, unlike GREML, has a closed-form solution. We assume random effects corresponding to the unscaled genotypes are $\beta_{i}∼𝒩(0,\frac{\sigma_{u}^{2}}{2mf_{i}(1-f_{i})})$.

$$V_{g}=V\left(\sum_{i=1}^{m}\beta_{i}x_{i}\right)=\sum_{i=1}^{m}\beta_{i}^{2}V(x_{i})$$

In expectation,

$$V(x_{i})=2\;E(\theta )\;f_{i}^{A}(1-f_{i}^{A})+2\{1-E(\theta )\}\;f_{i}^{B}(1-f_{i}^{B})+\;\;2\;E(\theta )\{1-E(\theta )\}{\left(f_{i}^{A}-f_{i}^{B}\right)}^{2}+2\;V(\theta ){\left(f_{i}^{A}-f_{i}^{B}\right)}^{2}\;\;={2f}_{i}(1-f_{i})+2\;V(\theta ){\left(f_{i}^{A}-f_{i}^{B}\right)}^{2}$$

And

$$V_{g}=\sum_{i=1}^{m}\beta_{i}^{2}\{{2f}_{i}(1-f_{i})+2\;V(\theta ){\left(f_{i}^{A}-f_{i}^{B}\right)}^{2}\}\;\;=\sum_{i=1}^{m}\frac{\sigma_{u}^{2}}{2mf_{i}(1-f_{i})}\{2f_{i}(1-f_{i})+2\;V(\theta ){\left(f_{i}^{A}-f_{i}^{B}\right)}^{2}\}\;\;=\frac{\sigma_{u}^{2}}{m}\sum_{i=1}^{m}\{1+V(\theta )\frac{{\left(f_{i}^{A}-f_{i}^{B}\right)}^{2}}{f_{i}(1-f_{i})}\}\;\;=\sigma_{u}^{2}+\underset{\begin{array}{c} \text{contribution of population structure}\\ \text{to the genic variance} \end{array}}{\underset{︸}{V(\theta )\frac{\sigma_{u}^{2}}{m}\sum_{i=1}^{m}\frac{{\left(f_{i}^{A}-f_{i}^{B}\right)}^{2}}{f_{i}(1-f_{i})}}}$$

Thus, the genetic variance can be decomposed into two terms, one that depends on the degree of population structure and one that does not.

The HE estimator of $V_{g}$ is based on the regression of products of (centered) phenotypes $y_{k}y_{l}$ for all pairs of individuals k≠l on the corresponding entries of the GRM (ψ) where $\psi_{kl}=\frac{\sum_{i=1}^{m}z_{ik}z_{il}}{m}$ and $z_{ik}$ is the centered and scaled genotype of individual k for locus i:

$${\hat{V}}_{g}=\frac{C(y_{k}y_{l},\psi_{kl})}{V(\psi_{kl})}\;\;=\frac{E(y_{k}y_{l}\psi_{kl})-E(y_{k}y_{l})\;E(\psi_{kl})}{E(\psi_{kl}^{2})-{E(\psi_{kl})}^{2}}\;\;=\frac{E(y_{k}y_{l}\psi_{kl})}{E(\psi_{kl}^{2})}$$

With the standard scaling, $z_{ik}=\frac{x_{ik}-2f_{i}}{\sqrt{2f_{i}(1-f_{i})}}$ and the corresponding effects are $u_{i}∼𝒩(0,\sigma_{u}^{2})$. In this case, the HE estimator is:

$${\hat{V}}_{g}=\frac{E(y_{k}y_{l}\psi_{kl})}{E(\psi_{kl}^{2})}=\frac{E(\sum_{i=1}^{m}u_{i}z_{ik}\sum_{i=1}^{m}u_{i}z_{ik}\psi_{kl})}{E(\psi_{kl}^{2})}\;\;=\frac{E(\sum_{i=1}^{m}u_{i}^{2}z_{ik}z_{il}\psi_{kl})}{E(\psi_{kl}^{2})}\;\;=\frac{E(u_{i}^{2})\;E\;(\sum_{i=1}^{m}z_{ik}z_{il}\psi_{kl})}{E(\psi_{kl}^{2})}=\frac{\frac{\sigma_{u}^{2}}{m}\;E(m\psi_{kl}^{2})}{E(\psi_{kl}^{2})}\;\;=\frac{\sigma_{u}^{2}\;E(\psi_{kl}^{2})}{E(\psi_{kl}^{2})}=\sigma_{u}^{2}$$

Which is not equal to $V_{g}$ because it does not capture the contribution of population structure. Next, we consider the case where the genotypes are standardized instead by the sample variance, i.e., $z_{kl}=\frac{x_{ik}-2f_{i}}{\sqrt{V(x_{i})}}$ such that $V(z_{i})=1$. We can derive $E(u_{i}^{2})$ corresponding to this scaling by noting that the genetic variance remains the same, i.e., the genetic variance is invariant under linear transformations of the genotype [14]:

$$\sum_{i=1}^{m}\beta_{i}^{2}V(x_{i})=\sum_{i=1}^{m}u_{i}^{2}V(z_{i})\;m\;E(u^{2})=\sigma_{u}^{2}+V(\theta )\frac{\sigma_{u}^{2}}{m}\sum_{i=1}^{m}\frac{{\left(f_{i}^{A}-f_{i}^{B}\right)}^{2}}{f_{i}(1-f_{i})}\;E(u^{2})=\frac{\sigma_{u}^{2}}{m}+V(\theta )\frac{\sigma_{u}^{2}}{m^{2}}\sum_{i=1}^{m}\frac{{\left(f_{i}^{A}-f_{i}^{B}\right)}^{2}}{f_{i}(1-f_{i})}$$

Note that because the genotypes are scaled properly, $E(\psi_{kl})=0$ and $V(\psi_{kl})=1$. Then, the HE estimator becomes:

$${\hat{V}}_{g}=\frac{E(y_{k}y_{l}\psi_{kl})}{E(\psi_{kl}^{2})}\;\;=\frac{E(u_{i}^{2})\;E(\sum_{i=1}^{m}z_{ik}z_{il})}{E(\psi_{kl}^{2})}=\left(\frac{\sigma_{u}^{2}}{m}+V(\theta )\frac{\sigma_{u}^{2}}{m^{2}}\sum_{i=1}^{m}\frac{{\left(f_{i}^{A}-f_{i}^{B}\right)}^{2}}{f_{i}(1-f_{i})}\right)\frac{m\;E(\psi_{kl}^{2})}{E(\psi_{kl}^{2})}\;\;=\sigma_{u}^{2}+V(\theta )\frac{\sigma_{u}^{2}}{m}\sum_{i=1}^{m}\frac{{\left(f_{i}^{A}-f_{i}^{B}\right)}^{2}}{f_{i}(1-f_{i})}$$

Which provides an unbiased estimate of the genic variance.

### 4 Genetic variance after correction for individual ancestry

In the main text, we stated that including individual ancestry as a fixed effect in GREML can lead to an underestimate of $V_{g}$ in the presence of population structure. Mixed effect models deal with fixed effects (ancestry in our case) by projecting them out of the phenotypes, and estimating the residual variance. This is conceptually equivalent to measuring the residual variance of the regression between phenotype and ancestry. As a result, any variance in the phenotype that is explained by ancestry is removed. To understand this quantitatively, it is helpful to decompose $V_{g}$ into components of variance explained by and variance orthogonal to ancestry:

$$V(g)=\underset{\begin{array}{c} \text{variance along}\\ \text{ancestry axis} \end{array}}{\underset{︸}{V\{\underset{\theta }{E}(g|\theta )\}}}+\underset{\begin{array}{c} \text{variance orthogonal}\\ \text{to ancestry axis} \end{array}}{\underset{︸}{\underset{\theta }{E}\{V(g|\theta )\}}}$$

We can express the residual variance as:

$$\underset{\theta }{E}\{V(g|\theta )\}=\underset{\theta }{E}\{V(\sum_{i=1}^{M}\beta_{i}^{2}x_{i}|\theta )\}\;\;=\underset{\theta }{E}\{\sum_{i=1}^{M}\beta_{i}^{2}V(x_{i}|\theta )\}+\underset{\theta }{E}\{\sum_{i\neq j}\beta_{i}\beta_{j}\;C(x_{i},x_{j}|\theta )\;\;=\sum_{i=1}^{M}\beta_{i}^{2}\underset{\theta }{E}\{V(x_{i}|\theta )\}+0\;\;=2\underset{\theta }{E}(\theta )\sum_{i=1}^{M}\beta^{2}\;f_{i}^{A}(1-f_{i}^{A})+2\{1-\underset{\theta }{E}(\theta )\}\sum_{i=1}^{M}\beta^{2}\;f_{i}^{B}(1-f_{i}^{B})\;\;+2\underset{\theta }{E}(\theta )\sum_{i=1}^{M}\beta^{2}\{1-\underset{\theta }{E}(\theta )\}{\left(f_{i}^{A}-f_{i}^{B}\right)}^{2}-2\;V(\theta )\sum_{i=1}^{M}\beta^{2}{\left(f_{i}^{A}-f_{i}^{B}\right)}^{2}$$

Note, that this represents the following components of $V_{g}$ from the main text: (1.1) + (1.2) − (1.3). Note, that (1.3), which is subtracted out, is always positive and depends on V(θ). Thus, the residual genetic variance will be underestimated, regardless of trait architecture, in the presence of population structure, i.e. when V(θ)>0.

### 5 Effect size of local ancestry

We define local ancestry $\gamma_{i}\in \{0,1,2\}$ as the number of alleles at locus i that trace their ancestry to population A. Thus, the local ancestry at locus i in individual k is a Binomial random variable with $E(\gamma_{i,k})=2\theta_{k}$. We define the ancestry value of an individual as the weighted sum of their local ancestry: $\sum_{i=1}^{m}\phi_{i}\gamma_{i}$ where $\phi_{i}=\beta_{i}(f_{i}^{B}-f_{i}^{A})$.

To show this, note that ϕ=E(y∣γ=1)−E(y∣γ=0) where $E(y|\gamma =1)=\int_{-\infty }^{\infty }yh(y|\gamma =1)$ and h is a density function. Our goal is to express ϕ in terms of β, which is equal to E(y∣x=1)−E(y∣x=0). Furthermore, $E(y|x=1)=\int_{-\infty }^{\infty }yh(y|x=1)$. We can express h(y∣γ) in terms of h(y∣x) as follows:

$$h(y|\gamma =1)=h(y|x=0)\;P(x=0|\gamma =1)+h(y|x=1)\;P(x=1|\gamma =1)+h(y|x=2)\;P(x=2|\gamma =1)\;\;=h(y|x=0)2(1-f^{A})(1-f^{A})+h(y|x=1)\{f^{A}(1-f^{B})+f^{B}(1-f^{A})\}+h(y|x=2)2f^{A}f^{B}$$

$$E(y|\gamma =1)=\int_{-\infty }^{\infty }yh(y|\gamma =1)dy\;\;=(1-f^{A})(1-f^{B})\int_{-\infty }^{\infty }yh(y|x=0)dy\;\;+\{f^{A}(1-f^{B})+f^{B}(1-f^{A})\}\int_{-\infty }^{\infty }yh(y|x=1)dy\;\;+f^{A}f^{B}\int_{-\infty }^{\infty }yh(y|x=2)dy\;\;=(1-f^{A})(1-f^{B})\;E(y|x=0)+\{f^{A}(1-f^{B})+f^{B}(1-f^{A})\}\;E(y|x=1)+f^{A}f^{B}\;E(y|x=2)\;\;=0+\{f^{A}(1-f^{B})+f^{B}(1-f^{A})\}\beta +f^{A}f^{B}2\beta \;\;=\beta f^{A}+\beta f^{B}$$

Similary, $E(y|\gamma =0)=2\beta \;f^{B}$ and $\phi =E(y|\gamma =1)-E(y|\gamma =0)=\beta (f^{B}-f^{A})$

### 6 Genetic variance due to local ancestry

(1)
$$V_{\gamma }=\;V(\sum_{i=1}^{m}\phi_{i}\gamma_{i})\;\;=\sum_{i=1}^{m}\phi_{i}^{2}V(\gamma_{i})+\sum_{i=1}^{m}\sum_{j\neq i}\phi_{i}\phi_{j}C(\gamma_{i},\gamma_{j})$$

We use the law of total variance and covariance to derive $V(\gamma_{i})$ and $C(\gamma_{i},\gamma_{j})$:

$$V(\gamma_{i})=E\{V(\gamma_{i}|\theta )\}+V\{E\{(\gamma_{i}|\theta )\}\;\;=E\{2\theta (1-\theta )\}+V(2\theta )\;\;=2\;E(\theta )-2\;E(\theta^{2})+4\;V(\theta )\;\;=2\;E(\theta )-2\;V(\theta )-2\;E{\left(\theta \right)}^{2}+4\;V(\theta )\;\;=2\;E(\theta )\{1-E(\theta )\}+2\;V(\theta )$$

$$C(\gamma_{i},\gamma_{j})=E\{C(\gamma_{i},\gamma_{j}|\theta )\}+C\{E(\gamma_{i},\gamma_{j}|\theta )\}\;\;=0+C(2\theta ,2\theta )=4\;V(\theta )$$

$$V_{\gamma }=2\;E(\theta )\{1-E(\theta )\}\sum_{i=1}^{m}\phi_{i}^{2}+2\;V(\theta )\sum_{i=1}^{m}\phi_{i}^{2}+4\;V(\theta )\sum_{i=1}^{m}\sum_{j\neq i}\phi_{i}\phi_{j}\;\;=2\;E(\theta )\{1-E(\theta )\}\sum_{i=1}^{m}\beta_{i}^{2}{\left(f_{i}^{B}-f_{i}^{A}\right)}^{2}\;\;+2\;V(\theta )\sum_{i=1}^{m}\beta_{i}^{2}{\left(f_{i}^{B}-f_{i}^{A}\right)}^{2}\;\;+4\;V(\theta )\sum_{i=1}^{m}\sum_{j\neq i}\beta_{i}\beta_{j}(f_{i}^{B}-f_{i}^{A})(f_{j}^{B}-f_{j}^{A})$$

We note, that
