# Supplementary material for: Interpreting SNP heritability in admixed populations
Source: bioRxiv. 2025 Apr 8:2023.08.04.551959. Originally published 2023 Aug 4. Preprint. [Version 4] doi: 10.1101/2023.08.04.551959 (PMC10418213; doi:10.1101/2023.08.04.551959)
Supplement: Supplement 2 [file NIHPP2023.08.04.551959v4-supplement-2.pdf]

bioRxiv preprint doi: https://doi.org/10.1101/2023.08.04.551959; this version posted April 8, 2025. The copyright holder for this preprint (which was not certified by peer review) is the author/funder, who has granted bioRxiv a license to display the preprint in perpetuity. It is made available under aCC-BY 4.0 International license.

Appendix

A1 Variance in ancestry

We denote variance and covariance with $\mathbb{V}(.)$ and $\mathbb{C}(.)$ and used the expressions in [27] to generate the expected value for the variance in ancestry, i.e., $\mathbb{V}(\theta)$. This is straightforward for the HI model, where at time t $\mathbb{V}(\theta_t) = \mathbb{V}(\theta_{t-1})\frac{(1+P_{t-1})}{2}$. $P_t = Cor(\theta_m, \theta_f)$ measures the strength of assortative mating, i.e, the correlation between the ancestry across mating pairs (θ_m, θ_f) at time t. For simplicity, we assumed this to be constant in every generation, i.e. $P_t = P_{t-1} = P$ following [27]. Since our notation slightly differs from [27], we re-derived the expression for $V(\theta_t)$ for the CGF model where population B receives a constant amount q of gene flow from population A in every generation. Note, that $\mathbb{E}(\theta_t) = q + (1-q)\,\mathbb{E}(\theta_{t-1})$. Then,

$$
\begin{aligned}
\mathbb{V}(\theta_t) =& \mathbb{E}(\theta_t^2) - \mathbb{E}(\theta_t)^2 \\
=& q + (1-q)\,\mathbb{E}\left[\left(\frac{\theta^m_{t-1}+\theta^f_{t-1}}{2}\right)\left(\frac{\theta^m_{t-1}+\theta^f_{t-1}}{2}\right)\right] - \{q + (1-q)\,\mathbb{E}(\theta_{t-1})\}^2 \\
=& q + \frac{(1-q)}{4}\{2\,\mathbb{E}(\theta^2_{t-1}) + 2\,\mathbb{E}(\theta^m_{t-1}\theta^f_{t-1})\} - \{q^2 + 2q(1-q)\,\mathbb{E}(\theta_{t-1}) + (1-q)^2\,\mathbb{E}(\theta_{t-1})^2\} \\
=& q + \frac{1-q}{2}\,\mathbb{E}(\theta^2_{t-1}) + \frac{1-q}{2}\,\mathbb{E}(\theta^m_{t-1}\theta^f_{t-1}) - q^2 - 2q(1-q)\,\mathbb{E}(\theta_{t-1}) - (1-q)^2\,\mathbb{E}(\theta_{t-1})^2 \\
=& q(1-q) + \frac{1-q}{2}\{\mathbb{V}(\theta_{t-1}) + \mathbb{E}(\theta_{t-1})^2\} + \frac{1-q}{2}\{\mathbb{C}(\theta^m_{t-1}, \theta^f_{t-1}) + \mathbb{E}(\theta_{t-1})^2\} - 2q(1-q)\,\mathbb{E}(\theta_{t-1}) - \mathbb{E}(\theta_{t-1})^2 \\
=& q(1-q) + \frac{1-q}{2}\,\mathbb{V}(\theta_{t-1}) + \frac{1-q}{2}\,\mathbb{E}(\theta_{t-1})^2 + \frac{1-q}{2}P_{t-1}\,\mathbb{V}(\theta_{t-1}) + \frac{1-q}{2}\,\mathbb{E}(\theta_{t-1})^2 - 2q(1-q)\,\mathbb{E}(\theta_{t-1}) - \mathbb{E}(\theta_{t-1})^2 \\
=& q(1-q) + \frac{1-q}{2}\,\mathbb{V}(\theta_{t-1})\{1+P_{t-1}\} + (1-q)\,\mathbb{E}(\theta_{t-1})^2 - 2q(1-q)\,\mathbb{E}(\theta_{t-1}) - (1-q)^2\,\mathbb{E}(\theta_{t-1})^2 \\
=& q(1-q)\,\mathbb{E}(\theta_{t-1})^2 + q(1-q)\{1 - 2\,\mathbb{E}(\theta_{t-1})\} + \frac{1-q}{2}\,\mathbb{V}(\theta_{t-1})\{1+P_{t-1}\}
\end{aligned}
$$

A2 Genetic variance

Let $y = g + e$, where y is the phenotypic value of an individual, g is the genotypic value, and e is random error. We assume additive effects such that $g = \sum_{i=1}^{m} \beta_i x_i$ where β_i is the effect size of the ith biallelic locus and $x_i \in \{0, 1, 2\}$ is the number of copies of the trait-increasing allele. Then, the genetic variance V_g is:

$$
V_g = \mathbb{V}(\sum_{i=1}^{m} \beta_i x_i) = \sum_{i=1}^{m} \beta_i^2\,\mathbb{V}(x_i) + \sum_{j \neq i} \beta_i \beta_j\,\mathbb{C}(x_i, x_j)
$$

In the following sections, we decompose $\mathbb{V}(x_i)$ and $\mathbb{C}(x_i, x_j)$ further as functions of ancestry.

A2.1 $\mathbb{V}(x_i)$

We first derive $\mathbb{V}(x_i)$ as a function of ancestry (θ) using the law of total variance:

bioRxiv preprint doi: https://doi.org/10.1101/2023.08.04.551959; this version posted April 8, 2025. The copyright holder for this preprint (which was not certified by peer review) is the author/funder, who has granted bioRxiv a license to display the preprint in perpetuity. It is made available under aCC-BY 4.0 International license.

$$\mathbb{V}(x_i) = \mathop{\mathbb{E}}_{\theta}\{\mathbb{V}(x_i|\theta)\} + \mathbb{V}\{\mathop{\mathbb{E}}_{\theta}(x_i|\theta)\}$$

where $\mathbb{E}_\theta$ represents the expectation taken over θ.

A2.1.1 $\mathbb{E}_\theta\{\mathbb{V}(x_i|\theta)\}$

We derive $\mathbb{V}(x_i|\theta)$ by further conditioning on the local ancestry at each locus.

$$\mathbb{V}(x_i|\theta) = \mathop{\mathbb{E}}_{\gamma}\{\mathbb{V}(x_i|\gamma,\theta)\} + \mathbb{V}\{\mathop{\mathbb{E}}_{\gamma}(x_i|\gamma,\theta)\}$$

where $\mathbb{E}_\gamma$ represents expectation taken over local ancestry. Since we are interested in the variance at a single locus, we will ignore the subscript i and denote the frequency of the trait-increasing allele in populations A and B with f^{A} and f^{B}, respectively.

$$\begin{aligned}
\mathop{\mathbb{E}}_{\gamma}\{\mathbb{V}(x_i|\gamma,\theta)\} =& \mathbb{V}(x_i|\gamma=0,\theta)\,\mathbb{P}(\gamma=0|\theta) + \mathbb{V}(x_i|\gamma=1,\theta)\,\mathbb{P}(\gamma=1|\theta) + \mathbb{V}(x_i|\gamma=2,\theta)\,\mathbb{P}(\gamma=2|\theta)\\
=& 2\,f^{\mathrm{B}}(1-f^{\mathrm{B}})(1-\theta)^2 + \{f^{\mathrm{A}}(1-f^{\mathrm{A}}) + f^{\mathrm{B}}(1-f^{\mathrm{B}})\}2\theta(1-\theta) + 2\,f^{\mathrm{A}}(1-f^{\mathrm{A}})\theta^2\\
=& (2\,f^{\mathrm{B}} - 2f^{A^2})(1-2\theta+\theta^2) + (f^{\mathrm{A}} - f^{A^2} + f^{\mathrm{B}} - f^{B^2})(2\theta-2\theta^2) + (2\,f^{\mathrm{A}} - 2f^{A^2})\theta^2\\
=& 2\,f^{\mathrm{B}} - 2\theta\,f^{\mathrm{B}} - 2f^{B^2} + 2\theta f^{B^2} + 2\theta\,f^{\mathrm{A}} - 2\theta f^{A^2}\\
=& 2\,f^{\mathrm{B}}(1-\theta) - 2f^{B^2}(1-\theta) + 2\theta\,f^{\mathrm{A}}(1-f^{\mathrm{A}})\\
=& 2\,f^{\mathrm{B}}(1-f^{\mathrm{B}})(1-\theta) + 2\theta\,f^{\mathrm{A}}(1-f^{\mathrm{A}})
\end{aligned}$$

To derive $\mathbb{V}\{\mathbb{E}_\gamma(x|\gamma,\theta)\}$, note that

$$\begin{aligned}
\mathop{\mathbb{E}}_{\gamma}(x|\gamma,\theta) =& \mathop{\mathbb{E}}_{\gamma}\{\mathbb{E}(x|\theta)\}\\
=& \mathbb{E}(x|\gamma=0,\theta)\,\mathbb{P}(\gamma=0|\theta) + \mathbb{E}(x|\gamma=1,\theta)\,\mathbb{P}(\gamma=1|\theta) + \mathbb{E}(x|\gamma=2,\theta)\,\mathbb{P}(\gamma=2|\theta)\\
=& 2\theta\,f^{\mathrm{A}} + 2(1-\theta)\,f^{\mathrm{B}}
\end{aligned}$$

And,

$$\begin{aligned}
\mathbb{V}\{\mathop{\mathbb{E}}_{\gamma}(x|\gamma,\theta)\} =& [\mathbb{E}(x|\gamma=0,\theta) - \mathbb{E}(x|\theta)]^2\,\mathbb{P}(\gamma=0|\theta)\\
&+ [\mathbb{E}(x|\gamma=1,\theta) - \mathbb{E}(x|\theta)]^2\,\mathbb{P}(\gamma=1|\theta)\\
&+ [\mathbb{E}(x|\gamma=2,\theta) - \mathbb{E}(x|\theta)]^2\,\mathbb{P}(\gamma=2|\theta)\\
=& \theta^2\left[2\,f^{\mathrm{A}} - \{2\theta\,f^{\mathrm{A}} + 2(1-\theta)\,f^{\mathrm{B}}\}\right]^2\\
&+ 2\theta(1-\theta)\left[f^{\mathrm{A}} + f^{\mathrm{B}} - \{2\theta\,f^{\mathrm{A}} + 2(1-\theta)\,f^{\mathrm{B}}\}\right]^2\\
&+ (1-\theta)^2\left[2\,f^{\mathrm{B}} - \{2\theta\,f^{\mathrm{A}} + 2(1-\theta)\,f^{\mathrm{B}}\}\right]^2\\
=& 2\theta(1-\theta)(f^{\mathrm{A}} - f^{\mathrm{B}})^2
\end{aligned}$$

bioRxiv preprint doi: https://doi.org/10.1101/2023.08.04.551959; this version posted April 8, 2025. The copyright holder for this preprint (which was not certified by peer review) is the author/funder, who has granted bioRxiv a license to display the preprint in perpetuity. It is made available under aCC-BY 4.0 International license.

Putting this together,

$$\begin{aligned}
\mathop{\mathbb{E}}_{\theta}\{\mathbb{V}(x_i|\theta)\} =& \mathop{\mathbb{E}}_{\theta}\{2\, f^{\mathrm{B}}(1-f^{\mathrm{B}})(1-\theta)+2\theta\, f^{\mathrm{A}}(1-f^{\mathrm{A}})+2\theta(1-\theta)(f^{\mathrm{A}}-f^{\mathrm{B}})^2\}\\
=&2\, f^{\mathrm{B}}(1-f^{\mathrm{B}})\{1-\mathop{\mathbb{E}}_{\theta}(\theta)\}+2\mathop{\mathbb{E}}_{\theta}(\theta)\, f^{\mathrm{A}}(1-f^{\mathrm{A}})+2\mathop{\mathbb{E}}_{\theta}(\theta-\theta^2)(f^{\mathrm{A}}-f^{\mathrm{B}})^2\\
=&2\, f^{\mathrm{B}}(1-f^{\mathrm{B}})\{1-\mathop{\mathbb{E}}_{\theta}(\theta)\}+2\mathop{\mathbb{E}}_{\theta}(\theta)\, f^{\mathrm{A}}(1-f^{\mathrm{A}})+2\{\mathop{\mathbb{E}}_{\theta}(\theta)-\mathop{\mathbb{E}}_{\theta}(\theta^2)\}(f^{\mathrm{A}}-f^{\mathrm{B}})^2\\
=&2\, f^{\mathrm{B}}(1-f^{\mathrm{B}})\{1-\mathop{\mathbb{E}}_{\theta}(\theta)\}+2\mathop{\mathbb{E}}_{\theta}(\theta)\, f^{\mathrm{A}}(1-f^{\mathrm{A}})+2\{\mathop{\mathbb{E}}_{\theta}(\theta)-\mathbb{V}(\theta)-\mathop{\mathbb{E}}_{\theta}(\theta)^2\}(f^{\mathrm{A}}-f^{\mathrm{B}})^2\\
=&2\, f^{\mathrm{B}}(1-f^{\mathrm{B}})\{1-\mathop{\mathbb{E}}_{\theta}(\theta)\}+2\mathop{\mathbb{E}}_{\theta}(\theta)\, f^{\mathrm{A}}(1-f^{\mathrm{A}})+2\mathop{\mathbb{E}}_{\theta}(\theta)(1-\mathop{\mathbb{E}}_{\theta}(\theta))(f^{\mathrm{A}}-f^{\mathrm{B}})^2-2\,\mathbb{V}(\theta)(f^{\mathrm{A}}-f^{\mathrm{B}})^2
\end{aligned}$$

A2.1.2 $\mathbb{V}\{\mathbb{E}_\theta(x_i|\theta)\}$

Recall from the previous section that $\mathbb{E}_\theta(x_i|\theta)=2\theta\, f^{\mathrm{A}}+2(1-\theta)\, f^{\mathrm{B}}$. Then,

$$\begin{aligned}
\mathbb{V}\{\mathop{\mathbb{E}}_{\theta}(x_i|\theta)\} =& \mathbb{V}\{2\theta\, f^{\mathrm{A}}+2(1-\theta)\, f^{\mathrm{B}}\}\\
=&4\,\mathbb{V}(\theta)f^{A^2}+4\,\mathbb{V}(1-\theta)f^{B^2}+2\,\mathbb{C}(2\theta\, f^{\mathrm{A}}, 2(1-\theta\, f^{\mathrm{B}})\\
=&4\,\mathbb{V}(\theta)f^{A^2}+4\,\mathbb{V}(1-\theta)f^{B^2}-8\, f^{\mathrm{A}}\, f^{\mathrm{B}}\,\mathbb{V}(\theta)\\
=&4\,\mathbb{V}(\theta)(f^{\mathrm{A}}-f^{\mathrm{B}})^2
\end{aligned}$$

We are now ready to express $\mathbb{V}(x_i)$:

$$\begin{aligned}
\mathbb{V}(x_i) =&2\, f^{\mathrm{B}}(1-f^{\mathrm{B}})\{1-\mathop{\mathbb{E}}_{\theta}(\theta)\}+2\mathop{\mathbb{E}}_{\theta}(\theta)\, f^{\mathrm{A}}(1-f^{\mathrm{A}})+2\mathop{\mathbb{E}}_{\theta}(\theta)(1-\mathop{\mathbb{E}}_{\theta}(\theta))(f^{\mathrm{A}}-f^{\mathrm{B}})^2\\
&-2\,\mathbb{V}(\theta)(f^{\mathrm{A}}-f^{\mathrm{B}})^2+4\,\mathbb{V}(\theta)(f^{\mathrm{A}}-f^{\mathrm{B}})^2\\
=&2\mathop{\mathbb{E}}_{\theta}(\theta)\, f_{\mathrm{i}}^{\mathrm{A}}(1-f_{\mathrm{i}}^{\mathrm{A}})+2\{1-\mathop{\mathbb{E}}_{\theta}(\theta)\}\, f_{\mathrm{i}}^{\mathrm{B}}(1-f_{\mathrm{i}}^{\mathrm{B}})\\
&+2\mathop{\mathbb{E}}_{\theta}(\theta)\{1-\mathop{\mathbb{E}}_{\theta}(\theta)\}(f_{\mathrm{i}}^{\mathrm{A}}-f_{\mathrm{i}}^{\mathrm{B}})^2-2\,\mathbb{V}(\theta)(f_{\mathrm{i}}^{\mathrm{A}}-f_{\mathrm{i}}^{\mathrm{B}})^2
\end{aligned}$$

Note, that we can also express $V(x_i)$ as:

$$\mathbb{V}(x_i) = 2f_i(1-f_i)+2\,\mathbb{V}(\theta)(f_i^A-f_i^B)^2$$

where the second term is the contribution of population structure to the genetic variance at locus i.

A2.2 $\mathbb{C}(x_i, x_j)$

We can derive $\mathbb{C}(x_i, x_j)$ using the law of total covariance:

bioRxiv preprint doi: https://doi.org/10.1101/2023.08.04.551959; this version posted April 8, 2025. The copyright holder for this preprint (which was not certified by peer review) is the author/funder, who has granted bioRxiv a license to display the preprint in perpetuity. It is made available under aCC-BY 4.0 International license.

$$\begin{aligned}
\mathbb{C}(x_i, x_j) =& \mathop{\mathbb{E}}_{\theta}\{\mathbb{C}(x_i, x_j|\theta)\} + \mathbb{C}\{\mathop{\mathbb{E}}_{\theta}(x_i|\theta), \mathop{\mathbb{E}}_{\theta}(x_j|\theta)\} \\
=& 0 + \mathbb{C}\{2\, f_{\mathrm{i}}^{\mathrm{A}}\, \theta + 2\, f_{\mathrm{i}}^{\mathrm{B}}(1-\theta), 2\, f_{\mathrm{j}}^{\mathrm{A}}\, \theta + 2\, f_{\mathrm{j}}^{\mathrm{B}}(1-\theta)\} \\
=& \mathbb{C}(2\, f_{\mathrm{i}}^{\mathrm{A}}\, \theta, 2\, f_{\mathrm{j}}^{\mathrm{A}}\, \theta) + \mathbb{C}(2\, f_{\mathrm{i}}^{\mathrm{A}}\, \theta, 2\, f_{\mathrm{j}}^{\mathrm{B}}(1-\theta) + \\
& \mathbb{C}(2\, f_{\mathrm{i}}^{\mathrm{B}}(1-\theta), 2\, f_{\mathrm{j}}^{\mathrm{A}}\, \theta) + \mathbb{C}(2\, f_{\mathrm{i}}^{\mathrm{B}}(1-\theta), 2\, f_{\mathrm{j}}^{\mathrm{B}}(1-\theta)) \\
=& 4\, \mathbb{V}(\theta)(f_{\mathrm{i}}^{\mathrm{A}} - f_{\mathrm{i}}^{\mathrm{B}})(f_{\mathrm{j}}^{\mathrm{A}} - f_{\mathrm{j}}^{\mathrm{B}})
\end{aligned}$$

$\mathbb{E}_\theta\{\mathbb{C}(x_i, x_j|\theta)\} = 0$ because we assume that the loci are unlinked and therefore, x_i and x_j are conditionally independent. Putting this all together, we get the genetic variance in admixed populations as presented in the main text:

$$\begin{aligned}
V_g =& \sum_{i=1}^{m} \beta_i^2\, \mathbb{V}(x_i) + \sum_{j\neq i} \beta_i\beta_j\, \mathbb{C}(x_i, x_j) \\
=& \sum_{i=1}^{m} \beta_i^2 2\, \mathop{\mathbb{E}}_{\theta}(\theta)\, f_{\mathrm{i}}^{\mathrm{A}}(1 - f_{\mathrm{i}}^{\mathrm{A}}) + \sum_{i=1}^{m} \beta_i^2 2\{1 - \mathop{\mathbb{E}}_{\theta}(\theta)\}\, f_{\mathrm{i}}^{\mathrm{B}}(1 - f_{\mathrm{i}}^{\mathrm{B}}) \\
&+ \sum_{i=1}^{m} \beta_i^2 2\, \mathop{\mathbb{E}}_{\theta}(\theta)\{1 - \mathop{\mathbb{E}}_{\theta}(\theta)\}(f_{\mathrm{i}}^{\mathrm{A}} - f_{\mathrm{i}}^{\mathrm{B}})^2 + \\
&+ \sum_{i=1}^{m} \beta_i^2 2\, \mathbb{V}(\theta)(f_{\mathrm{i}}^{\mathrm{A}} - f_{\mathrm{i}}^{\mathrm{B}})^2] \\
&+ \sum_{j\neq i} \beta_i\beta_j 4\, \mathbb{V}(\theta)(f_{\mathrm{i}}^{\mathrm{A}} - f_{\mathrm{i}}^{\mathrm{B}})(f_{\mathrm{j}}^{\mathrm{A}} - f_{\mathrm{j}}^{\mathrm{B}})
\end{aligned}$$

The only difference being that in the main text we use $\mathbb{E}$ instead of $\mathbb{E}_\theta$ for simplicity. With two 'unadmixed' source populations with equal number of individuals, $\mathbb{E}(\theta) = 0.5$ and $\mathbb{V}(\theta) = \mathbb{E}(\theta)\{1-\mathbb{E}(\theta)\} = 0.25$ and V_g reduces to:

$$\begin{aligned}
V_g =& \mathbb{V}\left(\sum_{i=1}^{m} \beta_i x_i\right) = \sum_{i=1}^{m} \beta_i^2\, \mathbb{V}(x_i) + \sum_{j\neq i} \beta_i\beta_j\, \mathbb{C}(x_i, x_j) \\
=& \sum_{i=1}^{m} \beta_i^2\, \left[f_{\mathrm{i}}^{\mathrm{A}}(1 - f_{\mathrm{i}}^{\mathrm{A}}) + f_{\mathrm{i}}^{\mathrm{B}}(1 - f_{\mathrm{i}}^{\mathrm{B}})\right] \\
&+ \sum_{i=1}^{m} \beta_i^2 (f_{\mathrm{i}}^{\mathrm{A}} - f_{\mathrm{i}}^{\mathrm{B}})^2 \\
&+ \sum_{i\neq j} \beta_i\beta_j (f_{\mathrm{i}}^{\mathrm{A}} - f_{\mathrm{i}}^{\mathrm{B}})(f_{\mathrm{j}}^{\mathrm{A}} - f_{\mathrm{j}}^{\mathrm{B}})
\end{aligned}$$

A3 Genetic variance after correction for individual ancestry

It can also be helpful to decompose V_g into components of variance explained by and variance orthogonal to ancestry:

bioRxiv preprint doi: https://doi.org/10.1101/2023.08.04.551959; this version posted April 8, 2025. The copyright holder for this preprint (which was not certified by peer review) is the author/funder, who has granted bioRxiv a license to display the preprint in perpetuity. It is made available under aCC-BY 4.0 International license.

$$\mathbb{V}(g) = \underbrace{\mathbb{V}_{\theta}\{\mathbb{E}(g|\theta)\}}_{\substack{\text{variance along} \\ \text{ancestry axis}}} + \underbrace{\mathbb{E}_{\theta}\{\mathbb{V}(g|\theta)\}}_{\substack{\text{variance orthogonal} \\ \text{to ancestry axis}}}$$

We can express the residual variance as:

$$\begin{aligned}
\mathbb{E}_{\theta}\{\mathbb{V}(g|\theta)\} =& \mathbb{E}_{\theta}\{\mathbb{V}(\sum_{i=1}^{M} \beta_i^2 x_i|\theta)\} \\
=& \mathbb{E}_{\theta}\{\sum_{i=1}^{M} \beta_i^2\, \mathbb{V}(x_i|\theta)\} + \mathbb{E}_{\theta}\{\sum_{i\neq j} \beta_i\beta_j\, \mathbb{C}(x_i, x_j|\theta) \\
=& \sum_{i=1}^{M} \beta_i^2\, \mathbb{E}_{\theta}\{\mathbb{V}(x_i|\theta)\} + 0 \\
=& 2\,\mathbb{E}_{\theta}(\theta) \sum_{i=1}^{M} \beta^2\, f_{\mathrm{i}}^{\mathrm{A}}(1 - f_{\mathrm{i}}^{\mathrm{A}}) + 2\{1 - \mathbb{E}_{\theta}(\theta)\} \sum_{i=1}^{M} \beta^2\, f_{\mathrm{i}}^{\mathrm{B}}(1 - f_{\mathrm{i}}^{\mathrm{B}}) \\
&+ 2\,\mathbb{E}_{\theta}(\theta) \sum_{i=1}^{M} \beta^2\{1 - \mathbb{E}_{\theta}(\theta)\}(f_{\mathrm{i}}^{\mathrm{A}} - f_{\mathrm{i}}^{\mathrm{B}})^2 - 2\,\mathbb{V}(\theta) \sum_{i=1}^{M} \beta^2 (f_{\mathrm{i}}^{\mathrm{A}} - f_{\mathrm{i}}^{\mathrm{B}})^2
\end{aligned}$$

Note, that this represents the following components of V_g: (1.1) + (1.2) - (1.3).

A4 Haseman-Elston regression

The Haseman-Elston (HE) estimator of V_g is based on the regression of products of (centered) phenotypes $y_k y_l$ for all pairs of individuals $k \neq l$ on the corresponding entries of the GRM ($\boldsymbol{\psi}$) where $\psi_{kl} = \frac{\sum_{i=1}^{m} z_{ik} z_{il}}{m}$ and z_{ik} is the centered and scaled genotype of individual k for locus i. In this and the following sections, we denote the effect sizes corresponding to the scaled genotypes as u_i and show the impact of alternative scaling schemes of the genotype on the HE estimator.

$$\begin{aligned}
\hat{V_g} =& \frac{\mathbb{C}(y_k y_l, \psi_{kl})}{\mathbb{V}(\psi_{kl})} \\
=& \frac{\mathbb{E}_{kl}(y_k y_l \psi_{kl}) - \mathbb{E}_{kl}(y_k y_l)\,\mathbb{E}_{kl}(\psi_{kl})}{\mathbb{E}_{kl}(\psi_{kl}^2) - \mathbb{E}(\psi_{kl})^2} \\
=& \frac{\mathbb{E}_{kl}(y_k y_l \psi_{kl})}{\mathbb{E}_{kl}(\psi_{kl}^2)} = \frac{\mathbb{E}_{kl}\left(\sum_{i=1}^{m} u_i z_{ik} \sum_{i=1}^{m} u_i z_{il} \psi_{kl}\right)}{\mathbb{E}_{kl}(\psi_{kl}^2)} \\
=& \frac{\mathbb{E}_{kl}\left(\sum_{i=1}^{m} u_i^2 z_{ik} z_{il} \psi_{kl}\right)}{\mathbb{E}_{kl}(\psi_{kl}^2)} + \frac{\mathbb{E}_{kl}\left(\sum_{i=1}^{m} \sum_{j\neq i} u_i u_j z_{ik} z_{jl} \psi_{kl}\right)}{\mathbb{E}_{kl}(\psi_{kl}^2)}
\end{aligned}$$

Where $\mathbb{E}_{kl}$ represents the expectation over all $k \times l$ pairwise comparisons between individuals and the first and second terms represent the genic and LD components of the estimator.

bioRxiv preprint doi: https://doi.org/10.1101/2023.08.04.551959; this version posted April 8, 2025. The copyright holder for this preprint (which was not certified by peer review) is the author/funder, who has granted bioRxiv a license to display the preprint in perpetuity. It is made available under aCC-BY 4.0 International license.

A4.1 No directional LD

First, let's assume a genetic architecture where the effect sizes are random and there is no LD contribution, i.e., $\mathbb{E}_{ij}(u_i u_j) = 0$. We can further simplify the estimator as follows:

$$
\begin{aligned}
\hat{V_g} =& \frac{\mathbb{E}_{kl}\left(\sum_{i=1}^{m} u_i^2 z_{ik} z_{il} \psi_{kl}\right)}{\mathbb{E}_{kl}(\psi_{kl}^2)} + \frac{\mathbb{E}_{kl}\left(\sum_{i=1}^{m} \sum_{j \neq i} u_i u_j z_{ik} z_{jl} \psi_{kl}\right)}{\mathbb{E}_{kl}(\psi_{kl}^2)} \\
=& \frac{\mathbb{E}(u_i^2)\, \mathbb{E}_{kl}\left(\sum_{i=1}^{m} z_{ik} z_{il} \psi_{kl}\right)}{\mathbb{E}_{kl}(\psi_{kl}^2)} + \frac{\mathbb{E}(u_i u_j)\, \mathbb{E}_{kl}\left(\sum_{i=1}^{m} \sum_{j \neq i} z_{ik} z_{jl} \psi_{kl}\right)}{\mathbb{E}_{kl}(\psi_{kl}^2)} \\
=& \frac{\mathbb{E}_i(u_i^2)\, \mathbb{E}_{kl}(m \psi_{kl}^2)}{\mathbb{E}_{kl}(\psi_{kl}^2)} + 0 = m \mathop{\mathbb{E}}_{ij}(u_i^2)
\end{aligned}
$$

Thus, in the absence of directional LD, the estimator is a function of $\mathbb{E}(u_i^2)$.

A4.1.1 Scaling by $2f_i(1-f_i)$

With the standard scaling, the genotype at a given locus i is $z_i = \frac{x_i - 2f_i}{\sqrt{2f_i(1-f_i)}}$ where f_i is the frequency of the allele in the population. Under the random-effects model, this is equivalent to saying that the unscaled effects are $\beta_i \sim \mathcal{N}(0, \frac{\sigma_u^2}{2mf_i(1-f_i)})$ and the scaled effects are $u_i \sim \mathcal{N}(0, \frac{\sigma_u^2}{m})$. In a panmictic population, $V_g = \sum_{i=1}^{m} \beta_i^2 \mathbb{V}(x_i) = \sum_{i=1}^{m} \frac{\sigma_u^2}{2mf_i(1-f_i)} 2f_i(1-f_i) = \sigma_u^2$. Thus, in a panmictic population, $\mathbb{E}(\hat{V_g}) = \sigma_u^2 = V_g$, i.e., the standard scaling yields unbiased estimates of the genetic variance. In an admixed population,

$$
\begin{aligned}
V_g =& \sum_{i=1}^{m} \beta_i^2 \{2f_i(1-f_i) + 2\,\mathbb{V}(\theta)(f_i^A - f_i^B)^2\} \\
=& \sum_{i=1}^{m} \frac{\sigma_u^2}{2mf_i(1-f_i)} \{2f_i(1-f_i) + 2\,\mathbb{V}(\theta)(f_i^A - f_i^B)^2\} \\
=& \frac{\sigma_u^2}{m} \sum_{i=1}^{m} \{1 + \mathbb{V}(\theta) \frac{(f_\text{i}^\text{A} - f_\text{i}^\text{B})^2}{f_i(1-f_i)}\} \\
=& \sigma_u^2 + \underbrace{\mathbb{V}(\theta) \frac{\sigma_u^2}{m} \sum_{i=1}^{m} \frac{(f_\text{i}^\text{A} - f_\text{i}^\text{B})^2}{f_i(1-f_i)}}_{\substack{\text{contribution of population structure} \\ \text{to the genic variance}}}
\end{aligned}
$$

Thus, with the standard scaling, HE regression does not capture the contribution of population structure and therefore, gives a biased estimate of V_g.

A4.1.2 Scaling by $\mathbb{V}(x_i)$

Next, we consider the case where the genotypes are standardized instead by the sample variance, i.e., $z_{kl} = \frac{x_{ik} - 2f_i}{\sqrt{\mathbb{V}(x_i)}}$ such that $\mathbb{V}(z_i) = 1$. We can derive $\mathbb{E}(u_i^2)$ corresponding to this scaling by noting that the genetic variance is invariant under linear transformations of the genotype [25]:

bioRxiv preprint doi: https://doi.org/10.1101/2023.08.04.551959; this version posted April 8, 2025. The copyright holder for this preprint (which was not certified by peer review) is the author/funder, who has granted bioRxiv a license to display the preprint in perpetuity. It is made available under aCC-BY 4.0 International license.

$$\sum_{i=1}^{m} \beta_i^2 \, \mathbb{V}(x_i) = \sum_{i=1}^{m} u_i^2 \, \mathbb{V}(z_i)$$

$$m \, \mathbb{E}(u_i^2) = \sigma_u^2 + \mathbb{V}(\theta) \frac{\sigma_u^2}{m} \sum_{i=1}^{m} \frac{(f_{\mathrm{i}}^{\mathrm{A}} - f_{\mathrm{i}}^{\mathrm{B}})^2}{f_i(1-f_i)}$$

$$\mathbb{E}(u_i^2) = \frac{\sigma_u^2}{m} + \mathbb{V}(\theta) \frac{\sigma_u^2}{m^2} \sum_{i=1}^{m} \frac{(f_{\mathrm{i}}^{\mathrm{A}} - f_{\mathrm{i}}^{\mathrm{B}})^2}{f_i(1-f_i)}$$

Then, the HE estimator becomes:

$$\begin{aligned}\hat{V}_g =& m \, \mathbb{E}(u_i^2) \\ =& m \left(\frac{\sigma_u^2}{m} + \mathbb{V}(\theta) \frac{\sigma_u^2}{m^2} \sum_{i=1}^{m} \frac{(f_{\mathrm{i}}^{\mathrm{A}} - f_{\mathrm{i}}^{\mathrm{B}})^2}{f_i(1-f_i)} \right) \\ =& \sigma_u^2 + \mathbb{V}(\theta) \frac{\sigma_u^2}{m} \sum_{i=1}^{m} \frac{(f_{\mathrm{i}}^{\mathrm{A}} - f_{\mathrm{i}}^{\mathrm{B}})^2}{f_i(1-f_i)}\end{aligned}$$

Which provides an unbiased estimate of the genic variance. It's important to note that even though we assumed effect sizes under a random-effect model, the above result holds under a fixed-effect model as long as there is no directional LD. We discuss the implications of directional LD in the following section.

A4.2 Directional LD

Under the standard random-effect model, the effect sizes are assumed to be independent *in expectation*. We discussed in the main text how certain processes (e.g. selection and assortative mating) can induce directional LD across causal loci. But directional LD might arise even for neutral traits and under the random-effects model for any given realization of effects. This can lead to biases in both HE and GREML estimates of V_g, though the direction and reason for bias is different for the two methods. GREML does not have a closed-form solution so the exact estimand is difficult to derive. Here, we develop some intuition for HE regression.

A4.2.1 Scaling by $\mathbb{V}(x_i)$

To do this, let $\boldsymbol{u}' = [u_1, u_2, ..., u_m]$ represent the vector of a given realization of (fixed) effects corresponding to the standardized genotypes such that each locus contributes equally to σ_u^2, the genic variance, i.e., $u_i^2 = \frac{\sigma_u^2}{m}$. Let there be positive LD across loci such that all cross-product terms are $u_i u_j = \frac{\sigma_u^2}{m}$. Then, the genetic variance explained by all loci is:

$$\begin{aligned}V_g =& \sum_{i=1}^{m} u_i^2 \, \mathbb{V}(z_i) + \sum_{j \neq i} u_i u_j \, \mathbb{C}(z_i, z_j) \\ =& \sum_{i=1}^{m} u_i^2 + \sum_{j \neq i} u_i u_j \, \mathbb{C}(z_i, z_j) \\ =& \sigma_u^2 + \frac{\sigma_u^2}{m} \sum_{j \neq i} \mathbb{C}(z_i, z_j) \qquad (3)\end{aligned}$$

bioRxiv preprint doi: https://doi.org/10.1101/2023.08.04.551959; this version posted April 8, 2025. The copyright holder for this preprint (which was not certified by peer review) is the author/funder, who has granted bioRxiv a license to display the preprint in perpetuity. It is made available under aCC-BY 4.0 International license.

where $\mathbb{C}(z_i, z_j)$ is the LD between the ith and j^{th} loci that ranges from 0 (no LD) to 1 (perfect LD). Thus, the LD contribution to V_g ranges from 0 to $(m-1)\sigma_m^2$. In comparison, the HE estimator is:

$$\begin{aligned}
\hat{V}_g =& \frac{\mathbb{E}_{kl}\left(\sum_{i=1}^m u_i^2 z_{ik} z_{il} \psi_{kl}\right)}{\mathbb{E}_{kl}(\psi_{kl}^2)} + \frac{\mathbb{E}_{kl}\left(\sum_{i=1}^m \sum_{j\neq i} u_i u_j z_{ik} z_{jl} \psi_{kl}\right)}{\mathbb{E}_{kl}(\psi_{kl}^2)} \\
=& \frac{\mathbb{E}_{kl}\left(\sum_{i=1}^m \frac{\sigma_u^2}{m} z_{ik} z_{il} \sum_{w=1}^m z_{wk} z_{wl}/m\right)}{\mathbb{E}_{kl}(\sum_{i=1}^m z_{ik} z_{il}/m \sum_{w=1}^m z_{wk} z_{wl}/m)} + \frac{\mathbb{E}_{kl}\left(\sum_{i=1}^m \sum_{j\neq i} \frac{\sigma_u^2}{m} z_{ik} z_{jl} \sum_{w=1}^m z_{wk} z_{wl}/m\right)}{\mathbb{E}_{kl}(\sum_{i=1}^m z_{ik} z_{il}/m \sum_{w=1}^m z_{wk} z_{wl}/m)} \\
=& \sigma_u^2 + \sigma_u^2 \frac{\mathbb{E}_{kl}\left(\sum_{i=1}^m \sum_{j\neq i} z_{ik} z_{jl} z_{wk} z_{wl}\right)}{\mathbb{E}_{kl}(\sum_{i=1}^m z_{ik} z_{il} \sum_{w=1}^m z_{wk} z_{wl})}
\end{aligned} \tag{4}$$

This shows that the bias due to directional LD in the HE estimate of V_g does not come from the genic, but the LD component. When there is no LD, e.g. if the population has reached equilibrium after generations of random mating, this component goes to zero and both the estimate and V_g converge to the same value – the genic variance. The LD component is maximum when the ith and j^{th} loci are in perfect LD. In this case, i and j are exchangeable and the second term of the estimator reduces to $(m-1)\sigma_m^2$. Thus, HE regression should give an unbiased estimate of V_g, even in the presence of directional LD, but only when LD is perfect. For any other value $0 < C(z_i, z_j) < 1$, the estimate is biased (Fig. A1). An interpretable, analytical derivation of the second term in Eq. 4 is complicated but we illustrate the bias with simulations below.

For unlinked markers, $\mathbb{C}(z_i, z_j)$ is a function of $4\,\mathbb{V}(\theta)(f_{\mathrm{i}}^{\mathrm{A}} - f_{\mathrm{i}}^{\mathrm{B}})(f_{\mathrm{j}}^{\mathrm{A}} - f_{\mathrm{j}}^{\mathrm{B}})$ (see A2.1). Perfect LD arises when (i) both $f_{\mathrm{i}}^{\mathrm{A}} - f_{\mathrm{i}}^{\mathrm{B}} = 1$ and $f_{\mathrm{j}}^{\mathrm{A}} - f_{\mathrm{j}}^{\mathrm{B}} = 1$ and (ii) $\mathbb{V}(\theta)$ is maximum, which occurs at the time of admixture when source populations mix equally, i.e, $\mathbb{E}(\theta) = 0.5$. To generate a range of LD, we simulated an admixed population ($N = 1,000$) with equal number of individuals from populations A and B. Thus, $4\,\mathbb{V}(\theta) = 4\,\mathbb{E}(\theta)\{1 - \mathbb{E}(\theta)\} = 1$. We simulated genotypes for each individual at 50 'causal' loci where the difference between the frequencies in the source populations, $f_i^A - f_i^B \in [0, 1]$ with the condition that $\frac{f_i^A + f_i^B}{2} = 0.5$. We assigned each locus the same effect size (on the variance-standardized scale) of $+1/\sqrt{m}$ summing up to a genic variance of 1. The positive sign ensures positive LD across loci, i.e, all off-diagonal elements of $\boldsymbol{uu'}$ are set to $1/m$. For each simulation, we computed the expected and estimated LD component using the second terms in Eqs. 3 and 4, respectively, and averaged the results over 100 replications.

A4.2.2 Scaling by LD

In the main text, we showed that standardizing the genotypes at a locus by its covariance with other loci accounts for the bias for GREML and HE estimators. More specifically, the 'LD-scaled' genotypes can be written as $\boldsymbol{Z} = (\boldsymbol{X} - 2\boldsymbol{P})\boldsymbol{U}^{-1}$ where $\boldsymbol{P}$ is an $n \times m$ matrix such that all elements of the ith column contain the frequency of the ith SNP and $\boldsymbol{U}$ is the (upper triangular) square root of the LD matrix, i.e, $\boldsymbol{\Sigma} = \boldsymbol{U'U}$. Under this scheme, the standardized genotypes are uncorrelated and therefore, the second term in Eqs. 3 and 4 are zero. This reduces the estimator to the first term, representing the sum of squares of effect sizes, i.e. $\boldsymbol{u'u} = \sum_{i=1}^m u_i^2$. The effect sizes corresponding to the LD scaled genotypes

bioRxiv preprint doi: https://doi.org/10.1101/2023.08.04.551959; this version posted April 8, 2025. The copyright holder for this preprint (which was not certified by peer review) is the author/funder, who has granted bioRxiv a license to display the preprint in perpetuity. It is made available under aCC-BY 4.0 International license.

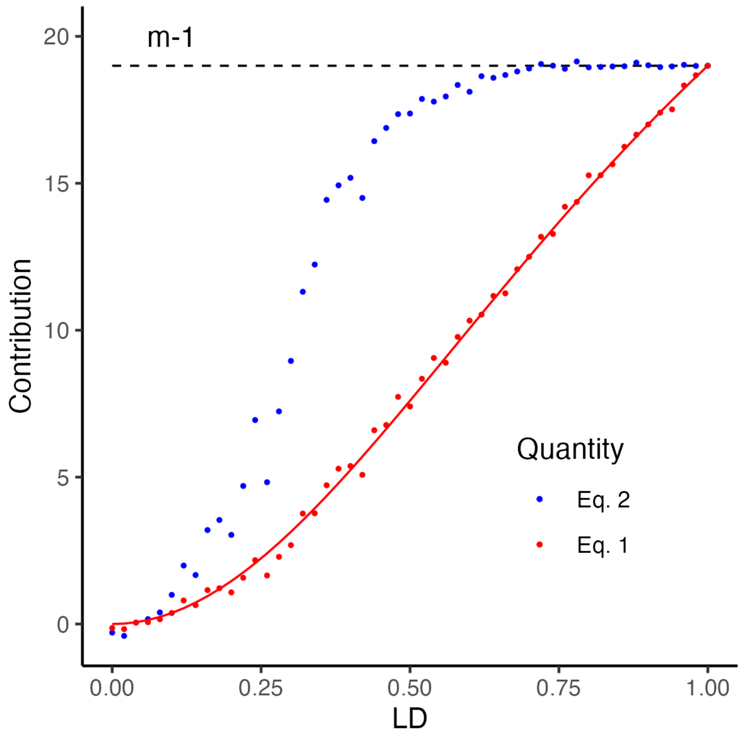

Figure A1: The behavior of the LD contribution (y-axis) to the genetic variance (red) and the Haseman-Elston regression estimate (blue) as a function of LD, i.e. $\mathbb{C}(z_i, z_j)$ (x-axis). Each point represents the contribution calculated from a random draw of genotypes, given $\mathbb{C}(z_i, z_j) \propto (f_{\mathrm{i}}^{\mathrm{A}} - f_{\mathrm{i}}^{\mathrm{B}})(f_{\mathrm{j}}^{\mathrm{A}} - f_{\mathrm{j}}^{\mathrm{B}})$. The red line represents the expected LD contribution and the black dashed line represents the contribution expected in the case of perfect LD.

bioRxiv preprint doi: https://doi.org/10.1101/2023.08.04.551959; this version posted April 8, 2025. The copyright holder for this preprint (which was not certified by peer review) is the author/funder, who has granted bioRxiv a license to display the preprint in perpetuity. It is made available under aCC-BY 4.0 International license.

are $\boldsymbol{u} = \boldsymbol{U\beta}$ and the sum of squares is:

$$\boldsymbol{u'u} = (\boldsymbol{U\beta})'\,(\boldsymbol{U\beta}) = \boldsymbol{\beta' U' U \beta} = \boldsymbol{\beta \Sigma \beta} = \sum_{i=1}^{m}\sum_{j=1}^{m} \beta_i \beta_j\, \mathbb{C}(x_i, x_j)$$

Which captures both the genic and LD contributions and therefore, provides an unbiased estimate of V_g.

A4.3 Genetic variance after correction for individual ancestry

In GREML it is common to include ancestry or principal components of the GRM to correct for population structure, which can lead to inflated estimates of heritability [4, 7]. In HE regression, a moment-matching approach (MMHE) can be used to account for covariates [37, 59]. To explain this, we use matrix notation. Recall the generative model $\boldsymbol{y} = \boldsymbol{g} + \boldsymbol{e} = \boldsymbol{Zu} + \boldsymbol{e}$ where $\boldsymbol{y}$ is an $n \times 1$ (centered) phenotype vector, $\boldsymbol{Z}$ is $n \times m$ matrix of standardized genotypes, and $\boldsymbol{u}$ is an $m \times 1$ vector of effect sizes. In the absence of covariates, HE regression estimates $\hat{\sigma}^2_u$ and $\hat{\sigma}^2_e$ by regressing the empirical covariance of the phenotypes onto the GRM: $\text{vec}(\boldsymbol{yy'}] = \text{vec}(\boldsymbol{Zuu'Z'}) + \text{vec}(\boldsymbol{ee'})$ where vec(.) is the vectorization operator. If the effects are independent, this simplifies to $\sigma^2_u\,\text{vec}(\boldsymbol{\psi}) + \sigma^2_e\,\text{vec}(\boldsymbol{I})$. Then $\hat{\sigma}^2_u$ and $\hat{\sigma}^2_e$ can be obtained from ordinary least squares (OLS).

Conceptually, the MMHE approach adjusts for covariates (e.g. sex, age, and ancestry) by projecting them out of both the phenotypes and genotypes [37]. We denote the projection matrix as $\boldsymbol{P} = \boldsymbol{C}(\boldsymbol{C'C})^{-1}\boldsymbol{C'}$ where $\boldsymbol{C}$ is the $n \times q$ design matrix of covariates. In our case, $\boldsymbol{C} = \begin{bmatrix}\boldsymbol{1} & \boldsymbol{\theta}\end{bmatrix}$ where $\boldsymbol{\theta}$ is the $n \times 1$ vector of individual ancestry. Then, let $\dot{\boldsymbol{y}} = \boldsymbol{My}$ and $\dot{\boldsymbol{Z}} = \boldsymbol{MZ}$ can be thought of as the residuals of a regression of $\boldsymbol{y}$ and $\boldsymbol{Z}$, respectively, on $\boldsymbol{\theta}$ and $\dot{\boldsymbol{\psi}} = \dot{\boldsymbol{Z}}\dot{\boldsymbol{Z}}'$ as the corresponding kinship matrix. Then, the ancestry-adjusted estimate of σ^2_u can be obtained by regressing $\text{vec}(\dot{\boldsymbol{y}}\dot{\boldsymbol{y}}')$ on $\text{vec}(\dot{\boldsymbol{\psi}}')$:

$$\begin{aligned}
\text{vec}(\dot{\boldsymbol{y}}\dot{\boldsymbol{y}}') &= \text{vec}(\dot{\boldsymbol{g}}\dot{\boldsymbol{g}}') + \text{vec}(\dot{\boldsymbol{e}}\dot{\boldsymbol{e}}') \\
&= \text{vec}((\boldsymbol{MZu})(\boldsymbol{MZu})') + \sigma^2_e \boldsymbol{I} \\
&= \text{vec}(\boldsymbol{MZuu'Z'M}) + \sigma^2_e \boldsymbol{I} \\
&= \text{vec}(\boldsymbol{MZ}\sigma^2_u\boldsymbol{Z'M}) + \sigma^2_e \boldsymbol{I} \\
&= \text{vec}(\dot{\boldsymbol{Z}}\sigma^2_u\dot{\boldsymbol{Z}}') + \sigma^2_e \boldsymbol{I} \\
&= \sigma^2_u\,\text{vec}(\dot{\boldsymbol{\psi}}) + \sigma^2_e \boldsymbol{I}_{(\boldsymbol{n}-\boldsymbol{q})}
\end{aligned}$$

The simplification in line 3 follows if we assume that (i) the off-diagonal terms of $\boldsymbol{uu'}$ are 0, i.e., the effects are independent, and (ii) all loci contribute equally to the genetic variance. The OLS estimates of σ^2_u and σ^2_e can be obtained by solving the following linear system [11, 37]:

$$\begin{bmatrix} \text{tr}(\boldsymbol{M\psi M\psi}) & \text{tr}(\boldsymbol{M\psi}) \\ \text{tr}(\boldsymbol{\psi}) & n-q \end{bmatrix} \begin{bmatrix} \sigma^2_u \\ \sigma^2_e \end{bmatrix} \begin{bmatrix} \boldsymbol{y'M\psi My} \\ \boldsymbol{y'My} \end{bmatrix}$$

where tr(.) represents the trace of the matrix. In practice, we use the more efficient algorithm in Ge *et al.* (2017) [37], which we used to estimate $\hat{\sigma}^2_u$.

bioRxiv preprint doi: https://doi.org/10.1101/2023.08.04.551959; this version posted April 8, 2025. The copyright holder for this preprint (which was not certified by peer review) is the author/funder, who has granted bioRxiv a license to display the preprint in perpetuity. It is made available under aCC-BY 4.0 International license.

A5 LD Score regression

LD score regression (LDSC) estimates h^2_{snp} under a random-effects model from GWAS summary statistics using method-of-moments. Here, we provide intuition for how LDSC can be biased in the presence of directional LD. We define χ^2_k as the marginal association statistic of the kth marker and $l_k = \sum_{w=1}^{W} r^2_{wk}$ as its LD score. Thus, LDSC estimates h^2_{snp} from the regression slope of χ^2_k on l_k over all k markers. To understand this, let's denote the estimated marginal effect of the kth marker as a function of its true marginal effect and some estimation error: $\hat{\tau}_k = \tau_k + \varepsilon_k$ assuming for simplicity that there is no residual bias due to stratification in the upstream GWAS. Then, $\mathbb{E}(\hat{\tau}_k) = \tau_k$ and $\mathbb{E}(\hat{\tau}^2_k) = \tau^2_k + \mathbb{E}(\varepsilon^2) = \tau^2_k + SE^2(\hat{\tau}_k)$. Furthermore, $\tau_k = \sum_{i=1}^{m} r_{ik}u_i$, where r_{ik} is the LD between the kth marker and the ith causal locus. If the individual causal effects are small, then $SE^2(\hat{\tau}_k) \approx 1/n$ and

$$\mathbb{E}(\chi^2_k) = \frac{\mathbb{E}(\hat{\tau}^2_k)}{SE^2(\hat{\tau}_k)} = \frac{\tau^2_k + SE^2(\hat{\tau}_k)}{SE^2(\hat{\tau}_k)} = \frac{\tau^2_k + 1/n}{1/n} = n\tau^2_k + 1$$

The expected slope of LD Score regression is:

$$\begin{aligned}
\hat{\beta}_{\chi^2,l} &= \frac{\mathbb{C}(\chi^2_k, l_k)}{\mathbb{V}(l_k)} = \frac{\mathbb{C}(n\tau^2_k + 1, l_k)}{\mathbb{V}(l_k)} = \frac{\mathbb{C}\left((\sum_{i=1}^{m} r_{ik}u_i)^2, l_k\right)}{\mathbb{V}(l_k)} \\
&= \frac{n\,\mathbb{C}\left(\sum_{i=1}^{m} r^2_{ik}u^2_i + 2\sum_{i=1}\sum_{j<i}^{m} r_{ik}r_{jk}u_iu_j, \sum_{i=1}^{m} r^2_{ik}\right)}{\mathbb{V}(l_k)} \\
&= \frac{n\,\mathbb{C}\left(\sum_{i=1}^{m} r^2_{ik}u^2_i, \sum_{i=1}^{m} r^2_{ik}\right) + n\,\mathbb{C}\left(2\sum_{i=1}\sum_{j<i}^{m} r_{ik}r_{jk}u_iu_j, \sum_{i=1}^{m} r^2_{ik}\right)}{\mathbb{V}(l_k)}
\end{aligned}$$

As with GREML and HE regression, LDSC assumes a polygenic, random-effects model where $u_i \sim \mathcal{N}(0, \frac{\sigma^2_u}{m})$. As such, the cross-product term inside the brackets is zero over variants i and j, and the slope reduces to:

$$\hat{\beta}_{\chi^2,l} = \frac{n\,\mathbb{C}\left(\sum_{i=1}^{m} r^2_{ik}u^2_i, \sum_{i=1}^{m} r^2_{ik}\right)}{\mathbb{V}(l_k)} = \frac{n\sigma^2_u}{m}$$

from which $\hat{V}_g$ and therefore, $\hat{h}^2_{snp}$ can be derived. To understand the behavior of LDSC under directional LD, consider a simple generative model with m causal variants, each with the same scaled (fixed) effect u in positive LD such that $V_g = \sum_{i=1}^{m} u^2_i\,\mathbb{V}(z_i) + 2\sum_{i=1}^{m}\sum_{j<i}\mathbb{C}(z_i, z_j) = \sum_{i=1}^{m} u^2_i + 2\sum_{i=1}^{m}\sum_{j<i} u_iu_jr_{ij} = mu^2 + 2u^2\sum_{i=1}^{m}\sum_{j<i} r_{ij}$. We further assume that causal variants are unlinked, that any LD between them is due to population structure, and that each causal variant is tagged by W markers. The marginal effect of a marker is a function of its LD with the causal loci, i.e., $\tau_k = \sum_{i=1}^{m} r_{ik}u_i = u\sum_{i=1}^{m} r_{ik}$. However, if, as per standard practice, individual ancestry was included as a covariate in the GWAS, then the marginal effect of the marker will only absorb the effect of the causal variant nearby but not of variants in LD due to population structure. Thus, if we note r_{kc} as the LD between the kth marker and the causal variant in its vicinity, $\tau_k = ur_{kc}$, $\tau^2_k = r^2_{kc}u^2$, and $\mathbb{E}(\chi^2_k) = nr^2_{kc}u^2 + 1$. In practice, LDSC regresses χ^2_k on $l_k = \sum_{w=1}^{W} r^2_{kw}$:

bioRxiv preprint doi: https://doi.org/10.1101/2023.08.04.551959; this version posted April 8, 2025. The copyright holder for this preprint (which was not certified by peer review) is the author/funder, who has granted bioRxiv a license to display the preprint in perpetuity. It is made available under aCC-BY 4.0 International license.

$$
\begin{aligned}
LDSC =& \frac{\mathbb{C}(\chi_k^2, l_k)}{\mathbb{V}(l_k)} = \frac{\mathbb{C}(\chi_k^2, \sum_w^W r_{kw}^2)}{\mathbb{V}(l_k)} = \frac{\sum_w^W \mathbb{C}(\chi_k^2, r_{kw}^2)}{\mathbb{V}(l_k)} \\
=& \frac{\mathbb{C}(\chi_k^2, r_{kc}^2)}{\mathbb{V}(l_k)} + \frac{\sum_{w \neq c} \mathbb{C}(\chi_k^2, r_{kw}^2)}{\mathbb{V}(l_k)} = \frac{nu^2 r_{kc}^2}{\mathbb{V}(l_k)}
\end{aligned}
$$

A6 Effect size of local ancestry

We define local ancestry $\gamma_i \in \{0, 1, 2\}$ as the number of alleles at locus i that trace their ancestry to population A. Thus, the local ancestry at locus i in individual k is a Binomial random variable with $\mathbb{E}(\gamma_{i,k}) = 2\theta_k$. We define the ancestry value of an individual as the weighted sum of their local ancestry: $\sum_{i=1}^m \phi_i \gamma_i$ where $\phi_i = \beta_i (f_{\mathrm{i}}^{\mathrm{B}} - f_{\mathrm{i}}^{\mathrm{A}})$.

To show this, note that $\phi = \mathbb{E}(y|\gamma = 1) - \mathbb{E}(y|\gamma = 0)$ where $\mathbb{E}(y|\gamma = 1) = \int_{-\infty}^{\infty} y h(y|\gamma = 1)$ and h is a density function. Our goal is to express ϕ in terms of β, which is equal to $\mathbb{E}(y|x = 1) - \mathbb{E}(y|x = 0)$. Furthermore, $\mathbb{E}(y|x = 1) = \int_{-\infty}^{\infty} y h(y|x = 1)$. We can express $h(y|\gamma)$ in terms of $h(y|x)$ as follows:

$$
\begin{aligned}
h(y|\gamma = 1) =& \; h(y|x = 0)\,\mathbb{P}(x = 0|\gamma = 1) + h(y|x = 1)\,\mathbb{P}(x = 1|\gamma = 1) + h(y|x = 2)\,\mathbb{P}(x = 2|\gamma = 1) \\
=& \; h(y|x = 0) 2(1 - f^{\mathrm{A}})(1 - f^{\mathrm{A}}) + h(y|x = 1)\{f^{\mathrm{A}}(1 - f^{\mathrm{B}}) + f^{\mathrm{B}}(1 - f^{\mathrm{A}})\} + h(y|x = 2) 2\, f^{\mathrm{A}}\, f^{\mathrm{B}}
\end{aligned}
$$

$$
\begin{aligned}
\mathbb{E}(y|\gamma = 1) =& \int_{-\infty}^{\infty} y h(y|\gamma = 1) dy \\
=& (1 - f^{\mathrm{A}})(1 - f^{\mathrm{B}}) \int_{-\infty}^{\infty} y h(y|x = 0) dy \\
& + \{f^{\mathrm{A}}(1 - f^{\mathrm{B}}) + f^{\mathrm{B}}(1 - f^{\mathrm{A}})\} \int_{-\infty}^{\infty} y h(y|x = 1) dy \\
& + f^{\mathrm{A}}\, f^{\mathrm{B}} \int_{-\infty}^{\infty} y h(y|x = 2) dy \\
=& (1 - f^{\mathrm{A}})(1 - f^{\mathrm{B}})\,\mathbb{E}(y|x = 0) + \{f^{\mathrm{A}}(1 - f^{\mathrm{B}}) + f^{\mathrm{B}}(1 - f^{\mathrm{A}})\}\,\mathbb{E}(y|x = 1) + f^{\mathrm{A}}\, f^{\mathrm{B}}\,\mathbb{E}(y|x = 2) \\
=& 0 + \{f^{\mathrm{A}}(1 - f^{\mathrm{B}}) + f^{\mathrm{B}}(1 - f^{\mathrm{A}})\}\beta + f^{\mathrm{A}}\, f^{\mathrm{B}}\, 2\beta \\
=& \beta\, f^{\mathrm{A}} + \beta\, f^{\mathrm{B}}
\end{aligned}
$$

Similary, $\mathbb{E}(y|\gamma = 0) = 2\beta\, f^{\mathrm{B}}$ and $\phi = \mathbb{E}(y|\gamma = 1) - \mathbb{E}(y|\gamma = 0) = \beta(f^{\mathrm{B}} - f^{\mathrm{A}})$

A7 Genetic variance due to local ancestry

$$
\begin{aligned}
V_\gamma =& \; \mathbb{V}(\sum_{i=1}^m \phi_i \gamma_i) \\
=& \sum_{i=1}^m \phi_i^2\, \mathbb{V}(\gamma_i) + \sum_{i=1}^m \sum_{j \neq i} \phi_i \phi_j\, \mathbb{C}(\gamma_i, \gamma_j)
\end{aligned} \tag{5}
$$

We use the law of total variance and covariance to derive $\mathbb{V}(\gamma_i)$ and $\mathbb{C}(\gamma_i, \gamma_j)$:

bioRxiv preprint doi: https://doi.org/10.1101/2023.08.04.551959; this version posted April 8, 2025. The copyright holder for this preprint (which was not certified by peer review) is the author/funder, who has granted bioRxiv a license to display the preprint in perpetuity. It is made available under aCC-BY 4.0 International license.

$$\begin{aligned}
\mathbb{V}(\gamma_i) &= \mathbb{E}\{\mathbb{V}(\gamma_i|\theta)\} + \mathbb{V}\{\mathbb{E}(\gamma_i|\theta)\} \\
&= \mathbb{E}\{2\theta(1-\theta)\} + \mathbb{V}(2\theta) \\
&= 2\,\mathbb{E}(\theta) - 2\,\mathbb{E}(\theta^2) + 4\,\mathbb{V}(\theta) \\
&= 2\,\mathbb{E}(\theta) - 2\,\mathbb{V}(\theta) - 2\,\mathbb{E}(\theta)^2 + 4\,\mathbb{V}(\theta) \\
&= 2\,\mathbb{E}(\theta)\{1-\mathbb{E}(\theta)\} + 2\,\mathbb{V}(\theta)
\end{aligned}$$

$$\begin{aligned}
\mathbb{C}(\gamma_i,\gamma_j) &= \mathbb{E}\{\mathbb{C}(\gamma_i,\gamma_j|\theta)\} + \mathbb{C}\{\mathbb{E}(\gamma_i,\gamma_j|\theta)\} \\
&= 0 + \mathbb{C}(2\theta,2\theta) = 4\,\mathbb{V}(\theta)
\end{aligned}$$

$$\begin{aligned}
V_\gamma =& 2\,\mathbb{E}(\theta)\{1-\mathbb{E}(\theta)\}\sum_{i=1}^{m}\phi_i^2 + 2\,\mathbb{V}(\theta)\sum_{i=1}^{m}\phi_i^2 + 4\,\mathbb{V}(\theta)\sum_{i=1}^{m}\sum_{j\neq i}\phi_i\phi_j \\
=& 2\,\mathbb{E}(\theta)\{1-\mathbb{E}(\theta)\}\sum_{i=1}^{m}\beta_i^2(f_{\mathrm{i}}^{\mathrm{B}} - f_{\mathrm{i}}^{\mathrm{A}})^2 \\
&+ 2\,\mathbb{V}(\theta)\sum_{i=1}^{m}\beta_i^2(f_{\mathrm{i}}^{\mathrm{B}} - f_{\mathrm{i}}^{\mathrm{A}})^2 \\
&+ 4\,\mathbb{V}(\theta)\sum_{i=1}^{m}\sum_{j\neq i}\beta_i\beta_j(f_{\mathrm{i}}^{\mathrm{B}} - f_{\mathrm{i}}^{\mathrm{A}})(f_{\mathrm{j}}^{\mathrm{B}} - f_{\mathrm{j}}^{\mathrm{A}})
\end{aligned}$$

bioRxiv preprint doi: https://doi.org/10.1101/2023.08.04.551959; this version posted April 8, 2025. The copyright holder for this preprint (which was not certified by peer review) is the author/funder, who has granted bioRxiv a license to display the preprint in perpetuity. It is made available under aCC-BY 4.0 International license.

1032 # Supplement

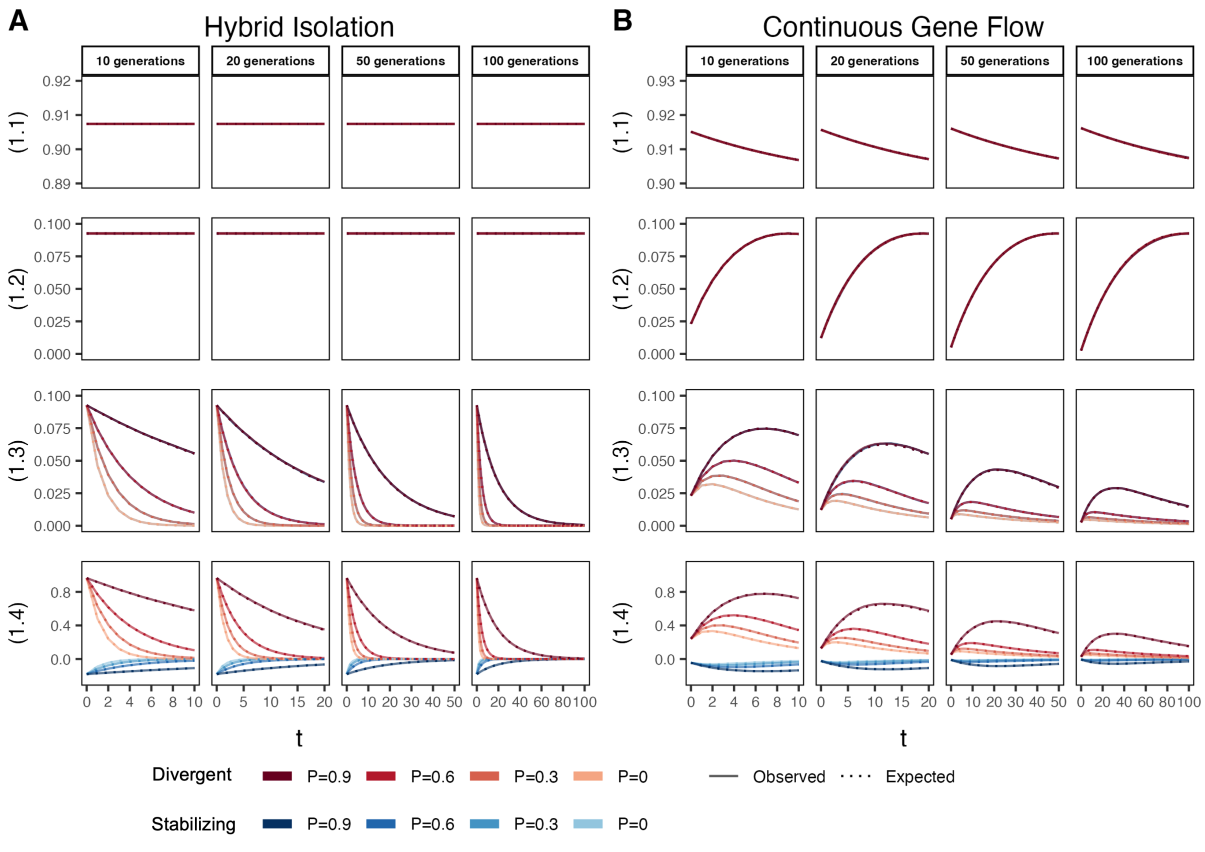

Figure S1: The behavior of the four components of genetic variance in admixed populations under the (A) HI and (B) CGF models. We assume that the mean ancestry proportion in the population is 0.5. The solid lines represent values observed in simulations averaged across ten replicates and the dotted lines represent the expected values based on Eq. 1 of the main text. The red and blue lines represent values for traits 1 and 2, respectively. P indicates the strength of assortative mating. P=0.6 is missing for simulations run for 50 and 100 generations and $\theta \in \{0.1, 0.2\}$ due to the difficulty in finding mate pairs (Methods).

bioRxiv preprint doi: https://doi.org/10.1101/2023.08.04.551959; this version posted April 8, 2025. The copyright holder for this preprint (which was not certified by peer review) is the author/funder, who has granted bioRxiv a license to display the preprint in perpetuity. It is made available under aCC-BY 4.0 International license.

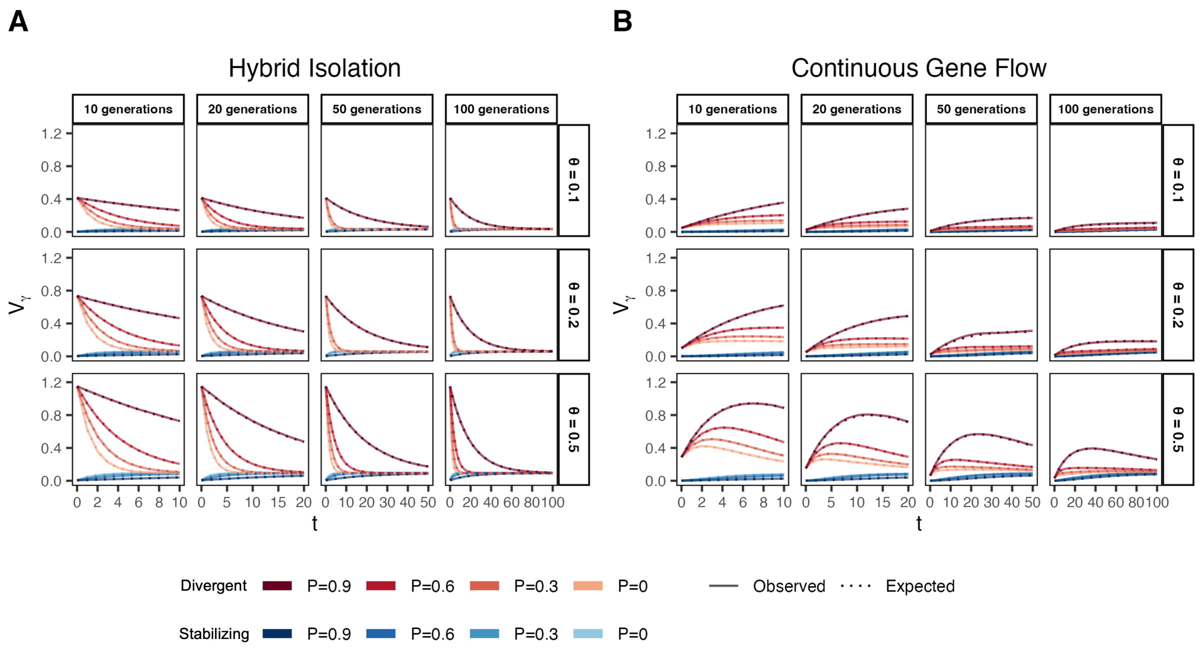

Figure S2: The behavior of the genetic variance due to local ancestry in admixed populations under the (A) HI and (B) CGF models. The solid lines represent values observed in simulations averaged across ten replicates and the dotted lines represent the expected values based on Eq. 1 of the main text. The red and blue lines represent values for traits 1 and 2, respectively. P indicates the strength of assortative mating. P=0.6 is missing for simulations run for 50 and 100 generations and $\theta \in \{0.1, 0.2\}$ due to the difficulty in finding mate pairs (Methods).

bioRxiv preprint doi: https://doi.org/10.1101/2023.08.04.551959; this version posted April 8, 2025. The copyright holder for this preprint (which was not certified by peer review) is the author/funder, who has granted bioRxiv a license to display the preprint in perpetuity. It is made available under aCC-BY 4.0 International license.

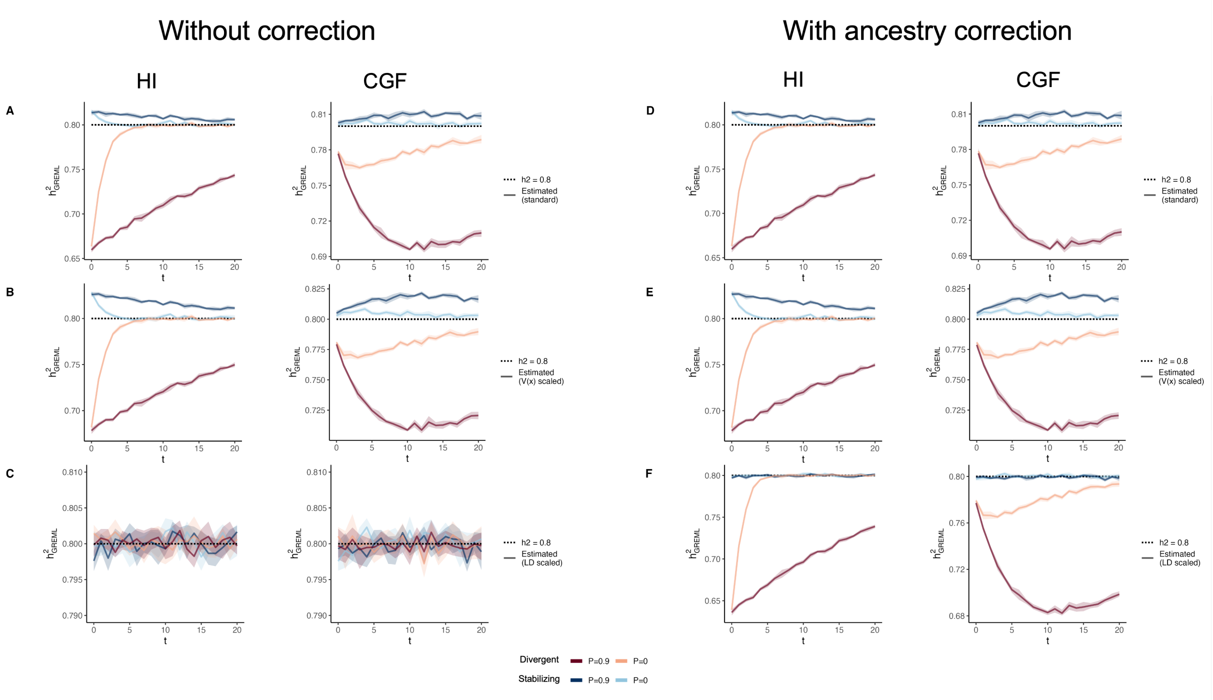

Figure S3: The behavior of GREML estimates of SNP heritability ($\hat{h}^2_{snp}$) in admixed populations under the HI (left column) and CGF (right column) models either without (A-C) or with (D-F) individual ancestry as a fixed effect. The solid lines represent $\hat{h}^2_{snp}$ averaged across ten replicates, with red and blue colors representing estimates for traits under divergent and stabilizing selection, respectively. (A, D) show the behavior of $\hat{h}^2_{snp}$ for the default scaling, (B, E) shows $\hat{h}^2_{snp}$ when the genotype at a locus is scaled by its sample variance ($\mathbb{V}(x)$ scaled), and (C, F) when it is scaled by the sample covariance (LD scaled). The shaded area represents the 95% confidence bands generated by bootstrapping (sampling with replacement 100 times) the point estimate reported by GCTA. The black dotted lines represent the expected heritability value given the simulation settings ($h^2 = 0.8$). P indicates the strength of assortative mating

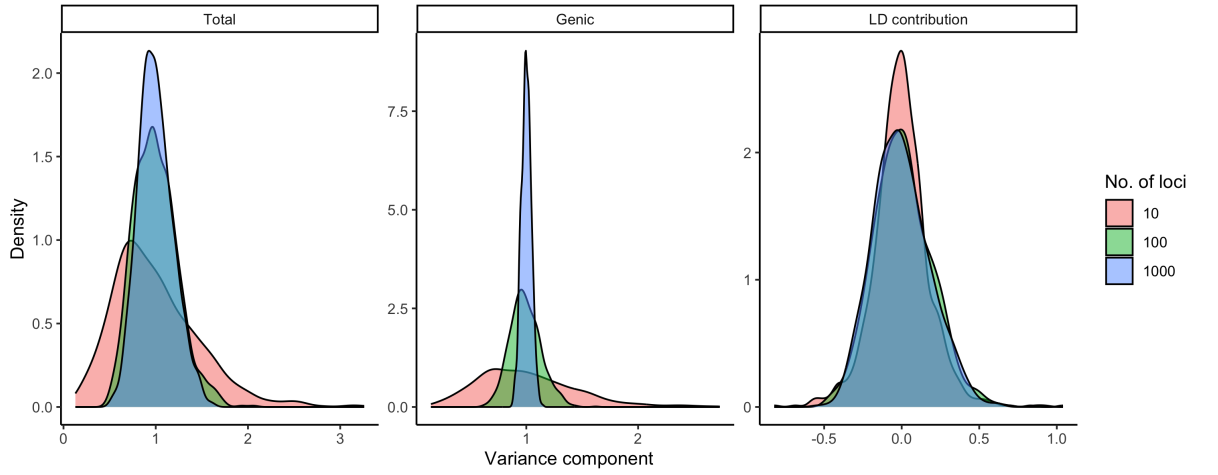

Figure S4: Distribution of the total genetic variance (left), genic variance (middle), and LD component (right) for a neutral trait simulated by drawing effects for 10, 100, or 1,000 causal variants in ASW. The total genetic variance is the sum of the genic and LD components.

bioRxiv preprint doi: https://doi.org/10.1101/2023.08.04.551959; this version posted April 8, 2025. The copyright holder for this preprint (which was not certified by peer review) is the author/funder, who has granted bioRxiv a license to display the preprint in perpetuity. It is made available under aCC-BY 4.0 International license.

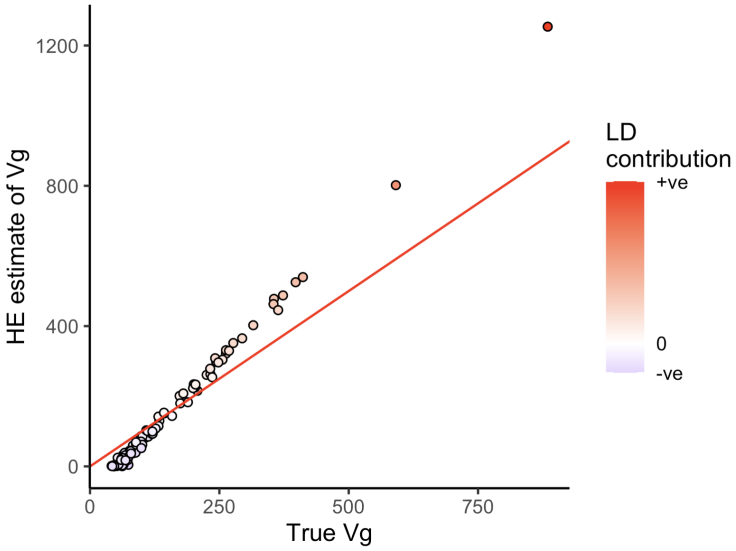

Figure S5: The effect of directional LD on Haseman-Elston estimate of genetic variance (V_g). Each individual point is an independent simulation where the effects were drawn from a normal distribution and applied to genotypes from an admixed population (Methods). The solid red line shows the $y = x$ line and the color of each point represents the contribution of LD to V_g.

bioRxiv preprint doi: https://doi.org/10.1101/2023.08.04.551959; this version posted April 8, 2025. The copyright holder for this preprint (which was not certified by peer review) is the author/funder, who has granted bioRxiv a license to display the preprint in perpetuity. It is made available under aCC-BY 4.0 International license.

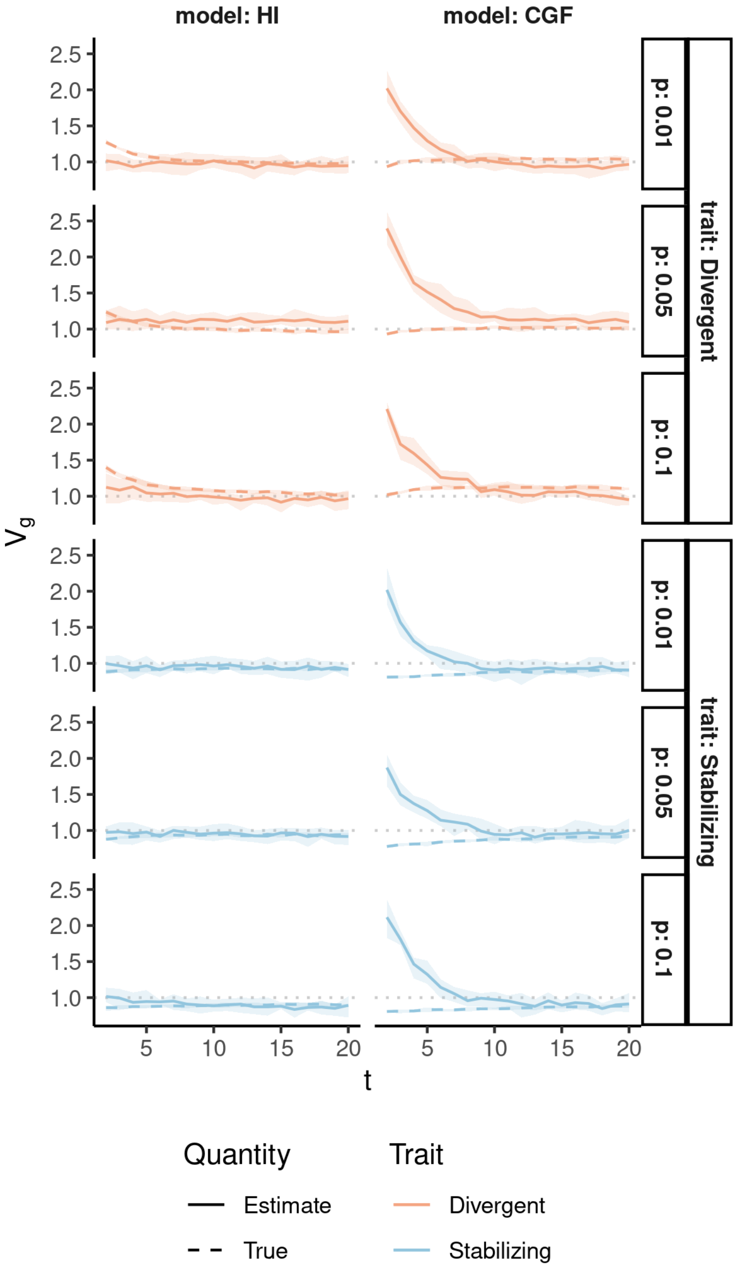

Figure S6: Behavior of LDSC estimates of V_g under the HI and CGF admixture models as a function of time since admixture (x-axis) for traits under directional (blue) and stabilizing selection (red) and with $p \in \{0.01, 0.05, 0.1\}$ proportion of causal variants. The dashed lines indicate (A) the simulated V_g whereas the solid lines show the estimated value. Dotted horizontal lines indicate the expected genic variance. Shaded ribbons indicate the 95% CI across ten replicates.

bioRxiv preprint doi: https://doi.org/10.1101/2023.08.04.551959; this version posted April 8, 2025. The copyright holder for this preprint (which was not certified by peer review) is the author/funder, who has granted bioRxiv a license to display the preprint in perpetuity. It is made available under aCC-BY 4.0 International license.

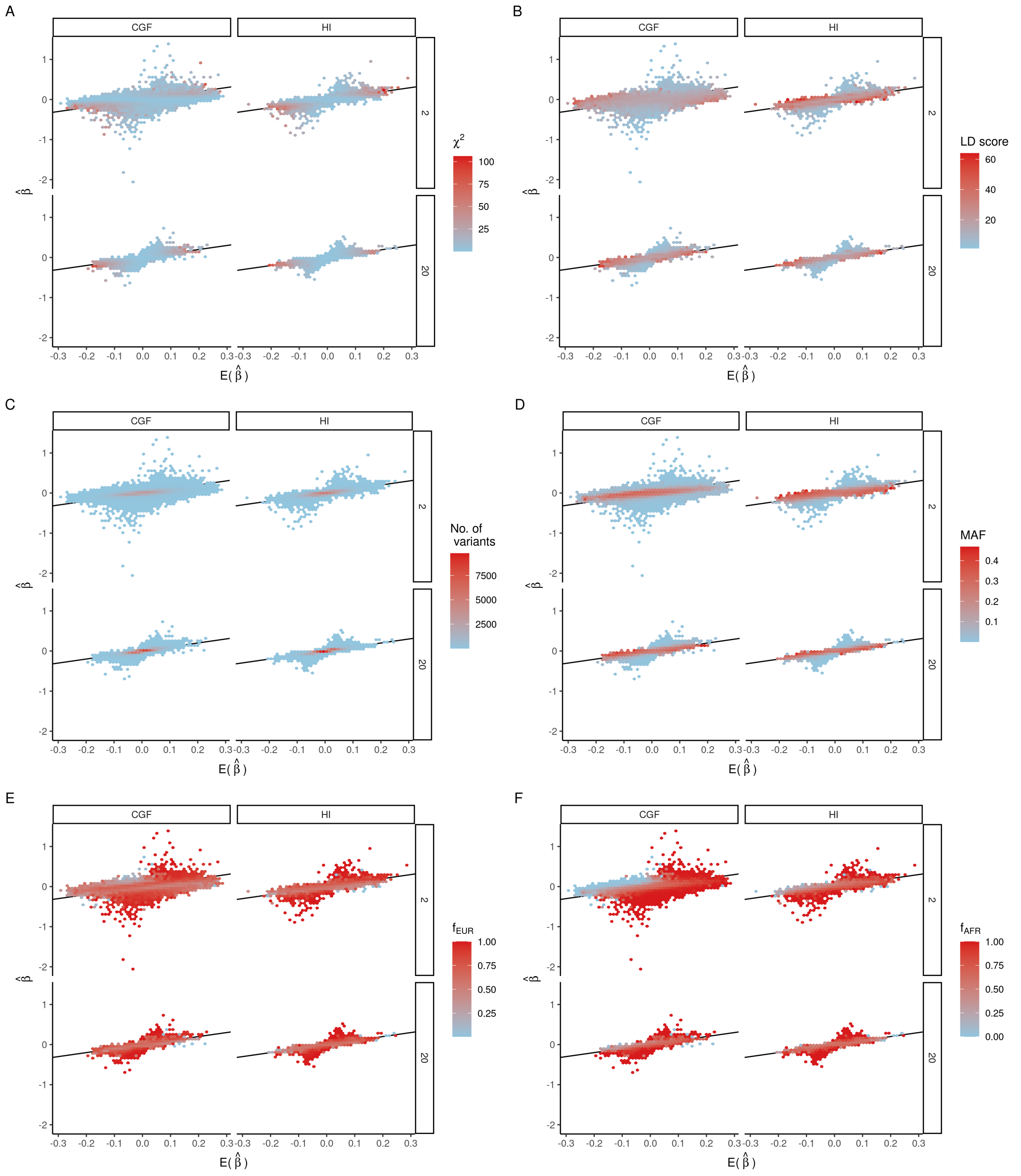

Figure S7: Expected vs Observed effect sizes of 87,564 variants on chromosome 2 for a GWAS carried out in 5,000 admixed individuals simulated under the HI and CGF models (panel columns) for generations 2 and 20 (panel rows) since admixture. Variants are grouped into bins and each bin is colored by the (A) mean χ^2, (B) mean LD score, (C) no. of variants, (D) mean MAF, (E) mean CEU frequency, and (F) mean YRI frequency across variants in that bin. The $\mathbb{E}(\hat{\beta}_j)$ of the jth SNP was computed as $\sum_{i=1}^{m} r_{ij}\beta_i$ where β_i is the simulated effect of the ith causal variant and r_{ij} is the genotypic correlation between them. The plot shows overdispersion in $\hat{\beta}$ due to population structure, especially in the 2nd generation of the CGF model. This appears to be driven by incoming variants that are common in CEU (E) but rare or absent in YRI (F).

bioRxiv preprint doi: https://doi.org/10.1101/2023.08.04.551959; this version posted April 8, 2025. The copyright holder for this preprint (which was not certified by peer review) is the author/funder, who has granted bioRxiv a license to display the preprint in perpetuity. It is made available under aCC-BY 4.0 International license.

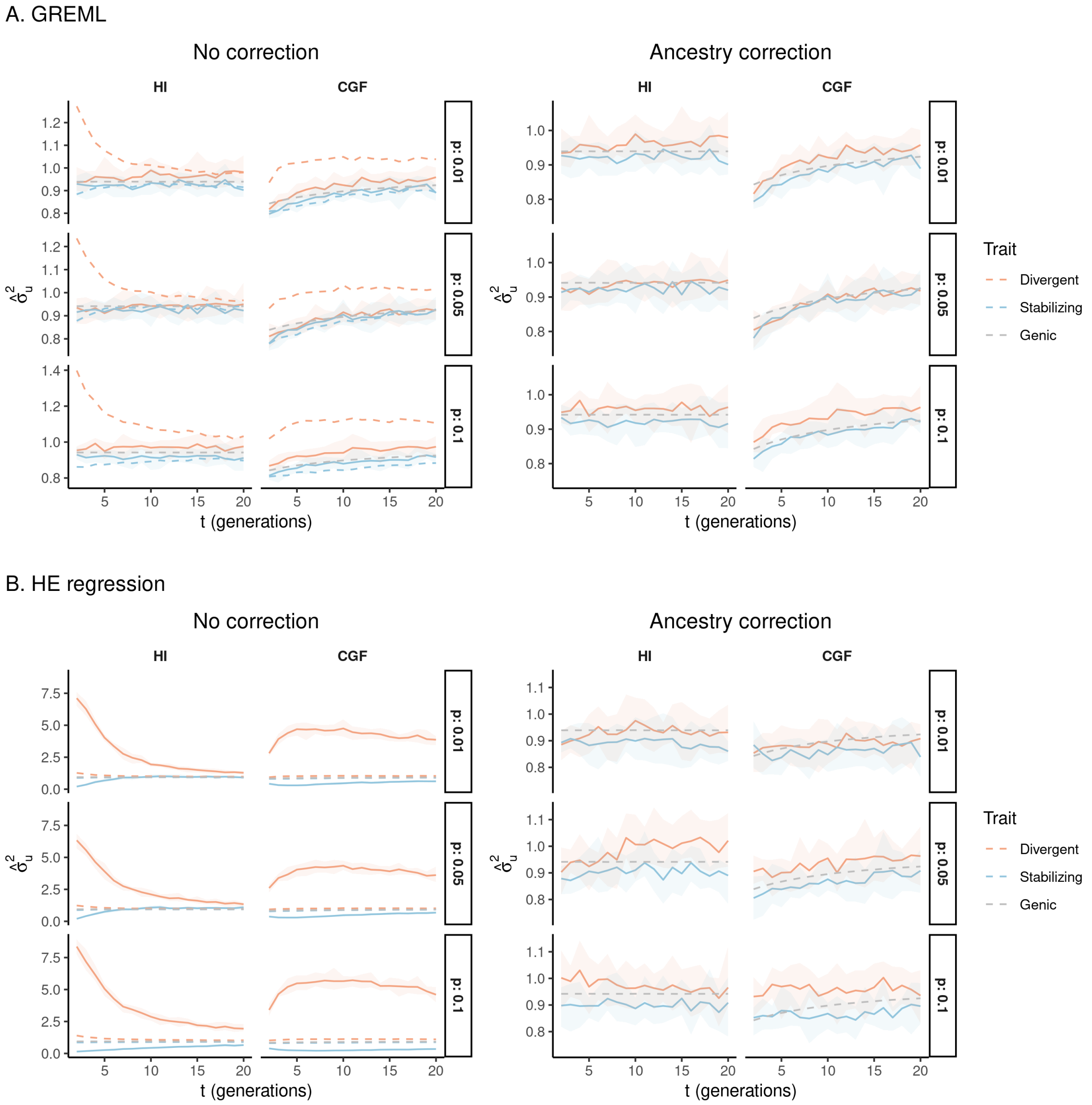

Figure S8: (A) GREML and (B) HE estimates of V_g (y-axis) on simulated genotype data of 5,000 admixed individuals for chromosome 2 under the HI and CGF models as a function of time since admixture (x-axis) and trait architecture (color). Results are also shown for varying proportion of causal variants (p) and whether or not ancestry (20 PCs) were included as covariates in the model. GREML and HE estimates are consistent with analytical expectations and simulations of unlinked markers.

bioRxiv preprint doi: https://doi.org/10.1101/2023.08.04.551959; this version posted April 8, 2025. The copyright holder for this preprint (which was not certified by peer review) is the author/funder, who has granted bioRxiv a license to display the preprint in perpetuity. It is made available under aCC-BY 4.0 International license.

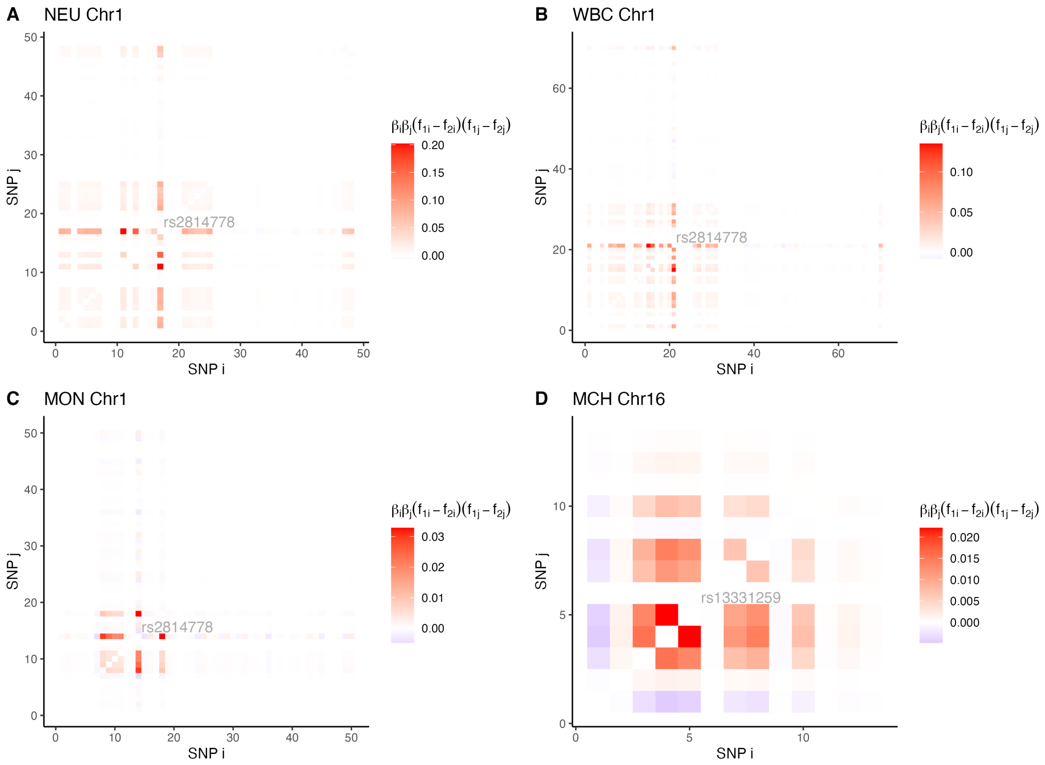

Figure S9: The LD contribution to the variance explained by variant pairs for (A) neutrophil counts (NEU), (B) white blood count (WBC), (C) monocyte count (MON), and (D) mean corpuscular hemoglobin (MCH). Only chromosomes where we suspected there was a disproportionate contribution to the variance explained are shown.

bioRxiv preprint doi: https://doi.org/10.1101/2023.08.04.551959; this version posted April 8, 2025. The copyright holder for this preprint (which was not certified by peer review) is the author/funder, who has granted bioRxiv a license to display the preprint in perpetuity. It is made available under aCC-BY 4.0 International license.

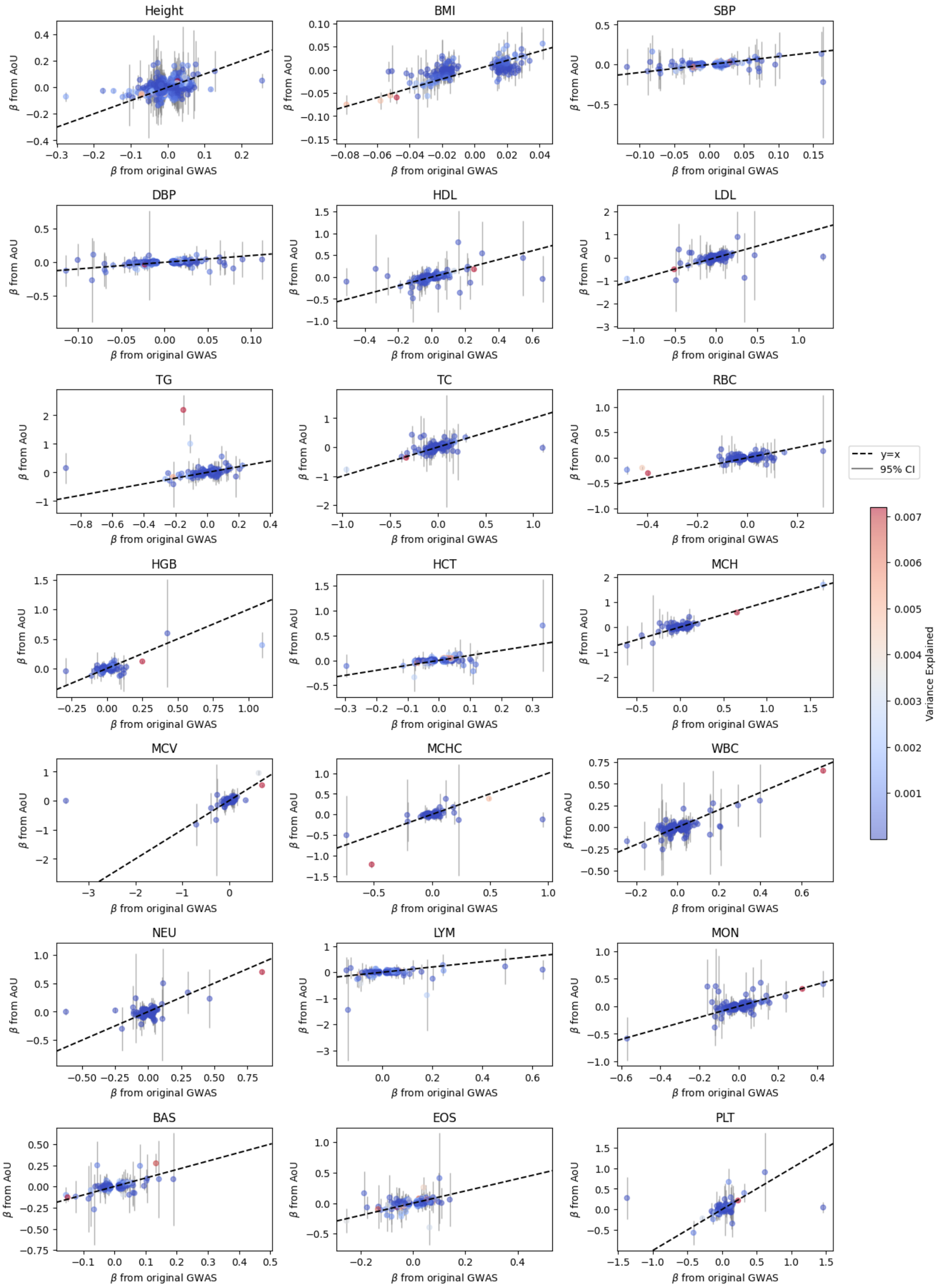

Figure S10: Effect sizes estimated in the AoU cohort compared to effect sizes from original GWAS summary statistics (Methods). Each β is colored by the amount of variance explained by that variant with low variance explained shown in blue and higher variance explained shown in red. Variance explained is computed as $\beta^2 2f(1-f)$, where f is the minor allele frequency in the African American AoU cohort. The $y = x$ line is shown as a dotted black line and the gray lines are 95% confidence intervals for each β in the re-estimated cohort.
