# Supplementary material for: Interpreting SNP heritability in admixed populations
Source: bioRxiv. 2024 Aug 6:2023.08.04.551959. Originally published 2023 Aug 4. Preprint. [Version 3] doi: 10.1101/2023.08.04.551959 (PMC10418213; doi:10.1101/2023.08.04.551959)
Supplement: Supplementary file 1 [file NIHPP2023.08.04.551959v3-supplement-1.pdf]

bioRxiv preprint doi: https://doi.org/10.1101/2023.08.04.551959; this version posted August 6, 2024. The copyright holder for this preprint (which was not certified by peer review) is the author/funder, who has granted bioRxiv a license to display the preprint in perpetuity. It is made available under aCC-BY 4.0 International license.

Supplement

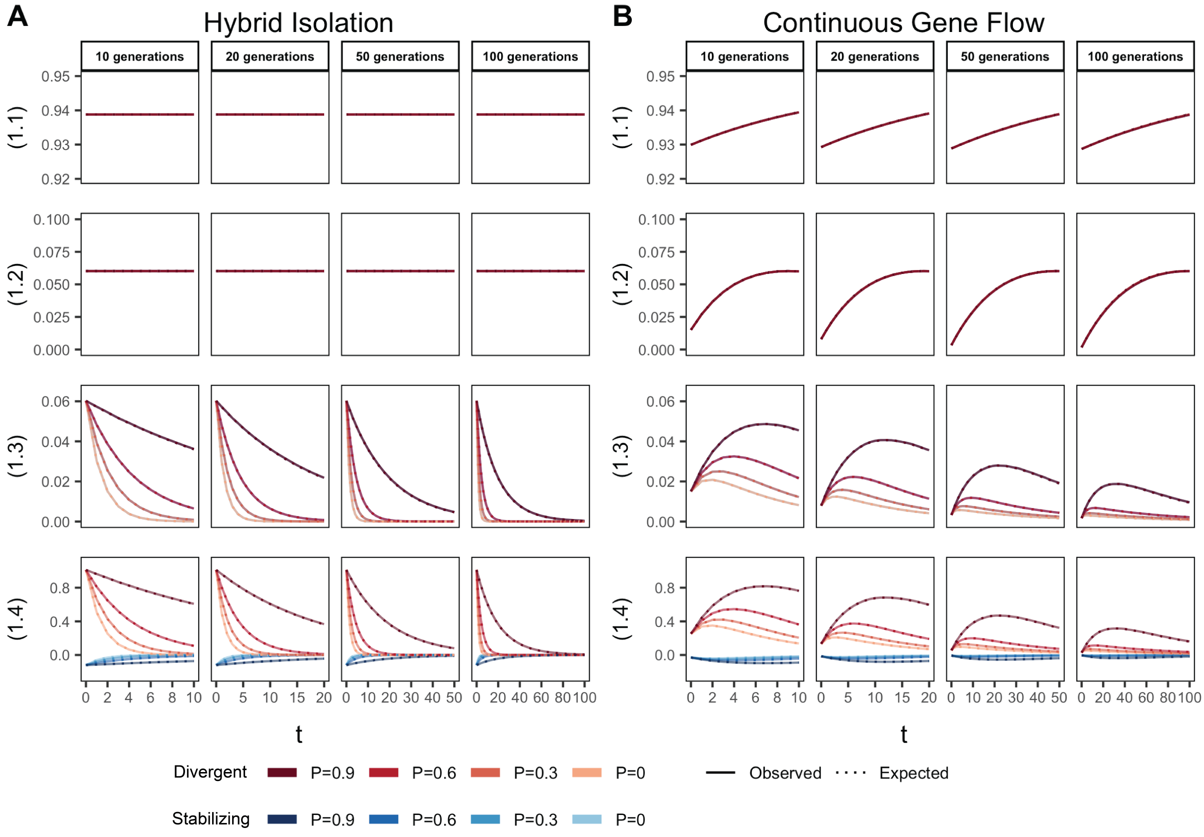

Figure S1: The behavior of the four components of genetic variance in admixed populations under the (A) HI and (B) CGF models. We assume that the mean ancestry proportion in the population is 0.5. The solid lines represent values observed in simulations averaged across ten replicates and the dotted lines represent the expected values based on Eq. 1 of the main text. The red and blue lines represent values for traits 1 and 2, respectively. P indicates the strength of assortative mating. P=0.6 is missing for simulations run for 50 and 100 generations and $\theta \in \{0.1, 0.2\}$ due to the difficulty in finding mate pairs (Methods).

bioRxiv preprint doi: https://doi.org/10.1101/2023.08.04.551959; this version posted August 6, 2024. The copyright holder for this preprint (which was not certified by peer review) is the author/funder, who has granted bioRxiv a license to display the preprint in perpetuity. It is made available under aCC-BY 4.0 International license.

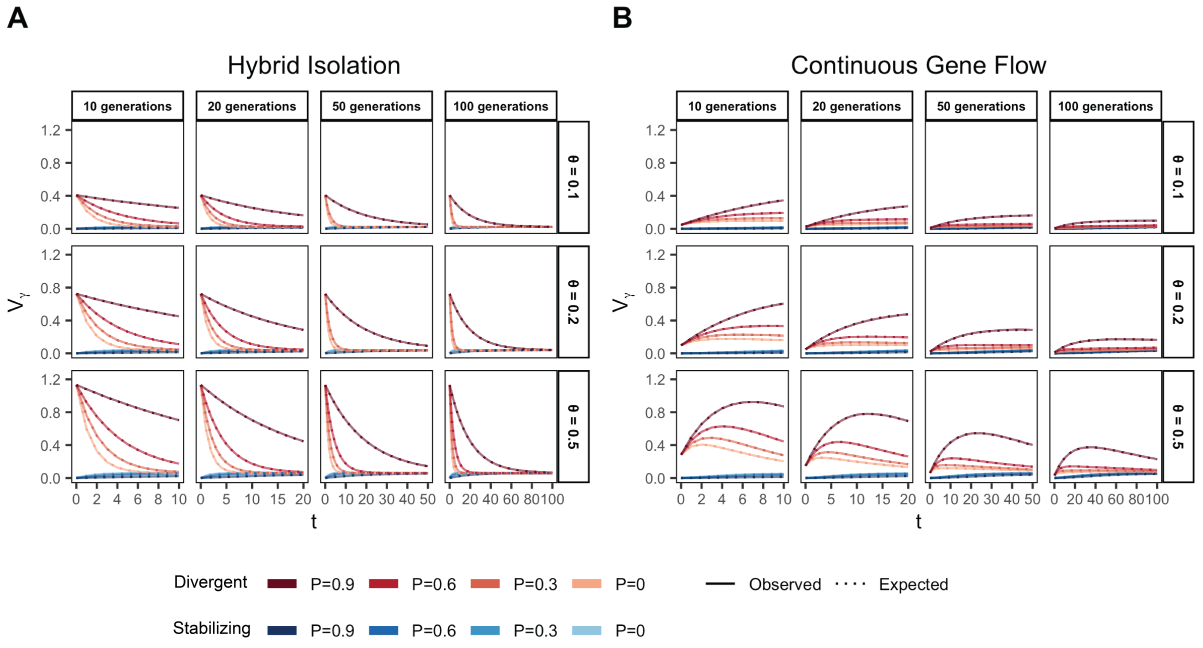

Figure S2: The behavior of the genetic variance due to local ancestry in admixed populations under the (A) HI and (B) CGF models. The solid lines represent values observed in simulations averaged across ten replicates and the dotted lines represent the expected values based on Eq. 1 of the main text. The red and blue lines represent values for traits 1 and 2, respectively. P indicates the strength of assortative mating. P=0.6 is missing for simulations run for 50 and 100 generations and $\theta \in \{0.1, 0.2\}$ due to the difficulty in finding mate pairs (Methods).

bioRxiv preprint doi: https://doi.org/10.1101/2023.08.04.551959; this version posted August 6, 2024. The copyright holder for this preprint (which was not certified by peer review) is the author/funder, who has granted bioRxiv a license to display the preprint in perpetuity. It is made available under aCC-BY 4.0 International license.

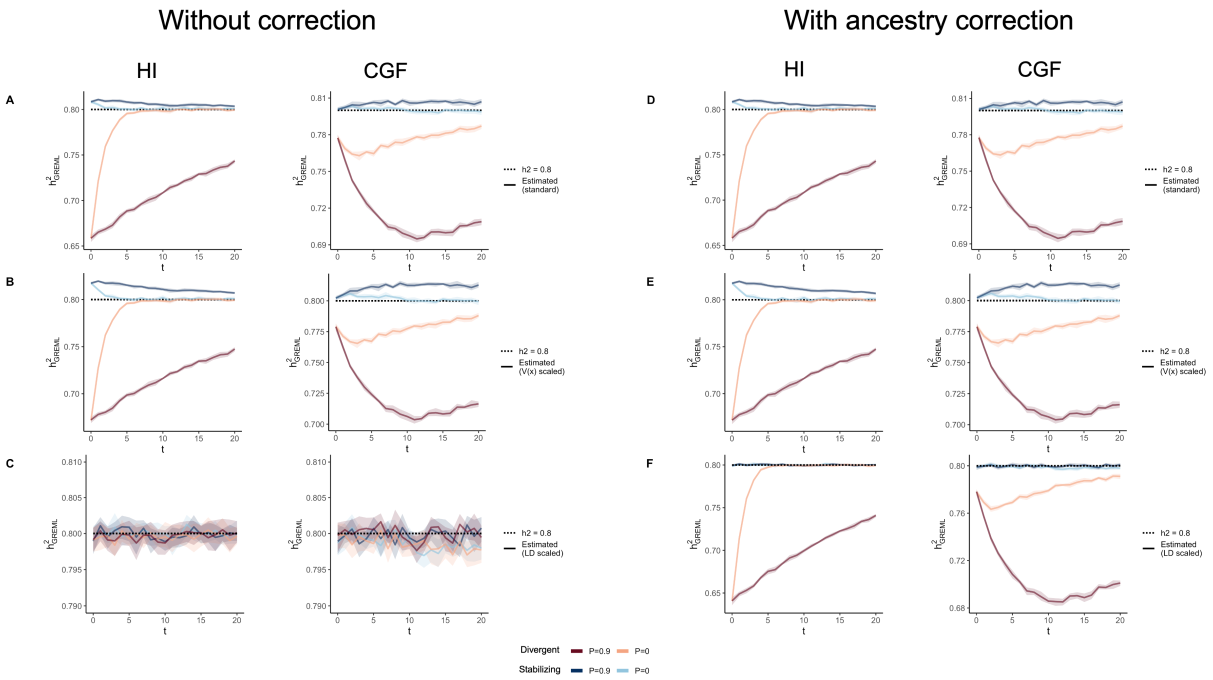

Figure S3: The behavior of GREML estimates of SNP heritability ($\hat{h}^2_{snp}$) in admixed populations under the HI (left column) and CGF (right column) models either without (A-C) or with (D-F) individual ancestry as a fixed effect. The solid lines represent $\hat{h}^2_{snp}$ averaged across ten replicates, with red and blue colors representing estimates for traits under divergent and stabilizing selection, respectively. (A, D) show the behavior of $\hat{h}^2_{snp}$ for the default scaling, (B, E) shows $\hat{h}^2_{snp}$ when the genotype at a locus is scaled by its sample variance ($\mathbb{V}(x)$ scaled), and (C, F) when it is scaled by the sample covariance (LD scaled). The shaded area represents the 95% confidence bands generated by bootstrapping (sampling with replacement 100 times) the point estimate reported by GCTA. The black dotted lines represent the expected heritability value given the simulation settings ($h^2 = 0.8$). P indicates the strength of assortative mating

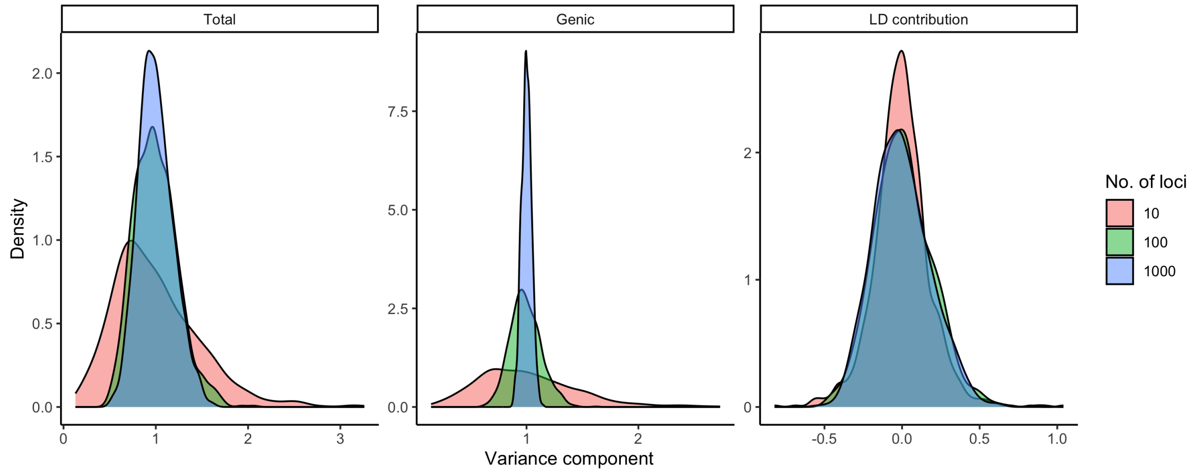

Figure S4: Distribution of the total genetic variance (left), genic variance (middle), and LD component (right) for a neutral trait simulated by drawing effects for 10, 100, or 1,000 causal variants in ASW. The total genetic variance is the sum of the genic and LD components.

bioRxiv preprint doi: https://doi.org/10.1101/2023.08.04.551959; this version posted August 6, 2024. The copyright holder for this preprint (which was not certified by peer review) is the author/funder, who has granted bioRxiv a license to display the preprint in perpetuity. It is made available under aCC-BY 4.0 International license.

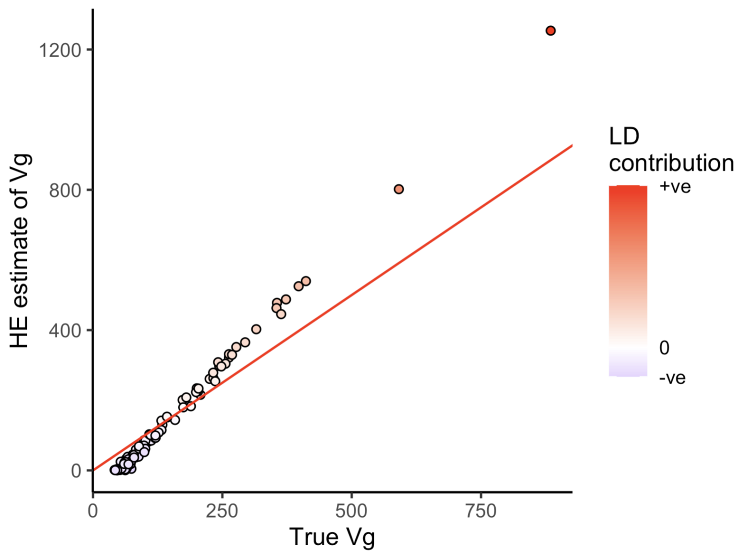

Figure S5: The effect of directional LD on Haseman-Elston estimate of genetic variance (V_g). Each individual point is an independent simulation where the effects were drawn from a normal distribution and applied to genotypes from an admixed population (Methods). The solid red line shows the $y = x$ line and the color of each point represents the contribution of LD to V_g.

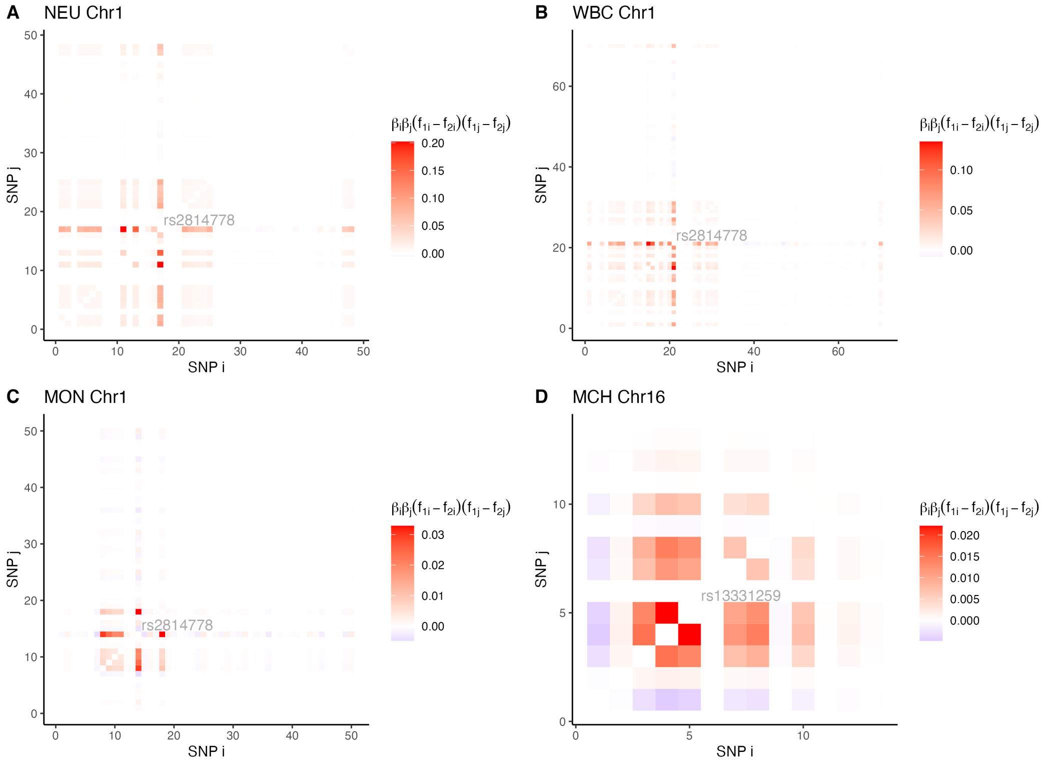

Figure S6: The LD contribution to the variance explained by variant pairs for (A) neutrophil counts (NEU), (B) white blood count (WBC), (C) monocyte count (MON), and (D) mean corpuscular hemoglobin (MCH). Only chromosomes where we suspected there was a disproportionate contribution to the variance explained are shown.

bioRxiv preprint doi: https://doi.org/10.1101/2023.08.04.551959; this version posted August 6, 2024. The copyright holder for this preprint (which was not certified by peer review) is the author/funder, who has granted bioRxiv a license to display the preprint in perpetuity. It is made available under aCC-BY 4.0 International license.

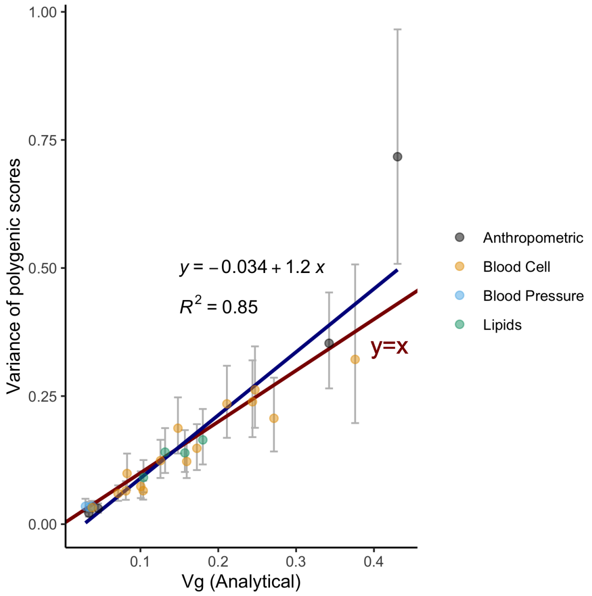

Figure S7: Expected variance explained estimated using Equation 1 (x-axis) vs the observed variance in polygenic scores (y-axis) in ASW are strongly correlated. Confidence intervals were generated by non-parametric bootstrap (Methods).
