# Supplementary material for: Interpreting SNP heritability in admixed populations
Source: bioRxiv. 2023 Nov 7:2023.08.04.551959. Originally published 2023 Aug 4. Preprint. [Version 2] doi: 10.1101/2023.08.04.551959 (PMC10418213; doi:10.1101/2023.08.04.551959)
Supplement: Supplementary file 1 [file NIHPP2023.08.04.551959v2-supplement-1.pdf]

bioRxiv preprint doi: https://doi.org/10.1101/2023.08.04.551959; this version posted November 7, 2023. The copyright holder for this preprint (which was not certified by peer review) is the author/funder, who has granted bioRxiv a license to display the preprint in perpetuity. It is made available under aCC-BY 4.0 International license.

Supplement

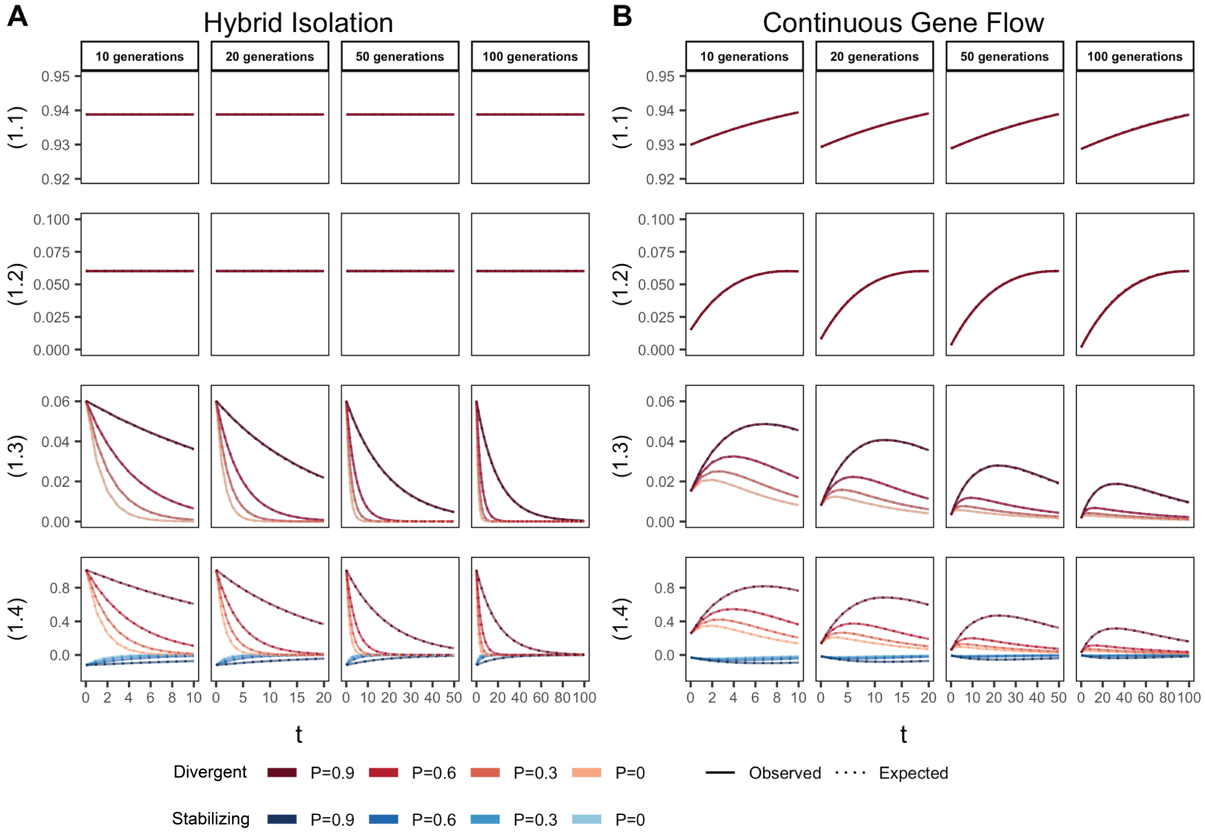

Figure S1: The behavior of the four components of genetic variance in admixed populations under the (A) HI and (B) CGF models. We assume that the mean ancestry proportion in the population is 0.5. The solid lines represent values observed in simulations averaged across ten replicates and the dotted lines represent the expected values based on Eq. 1 of the main text. The red and blue lines represent values for traits 1 and 2, respectively. P indicates the strength of assortative mating. P=0.6 is missing for simulations run for 50 and 100 generations and $\theta \in \{0.1, 0.2\}$ due to the difficulty in finding mate pairs (Methods).

bioRxiv preprint doi: https://doi.org/10.1101/2023.08.04.551959; this version posted November 7, 2023. The copyright holder for this preprint (which was not certified by peer review) is the author/funder, who has granted bioRxiv a license to display the preprint in perpetuity. It is made available under aCC-BY 4.0 International license.

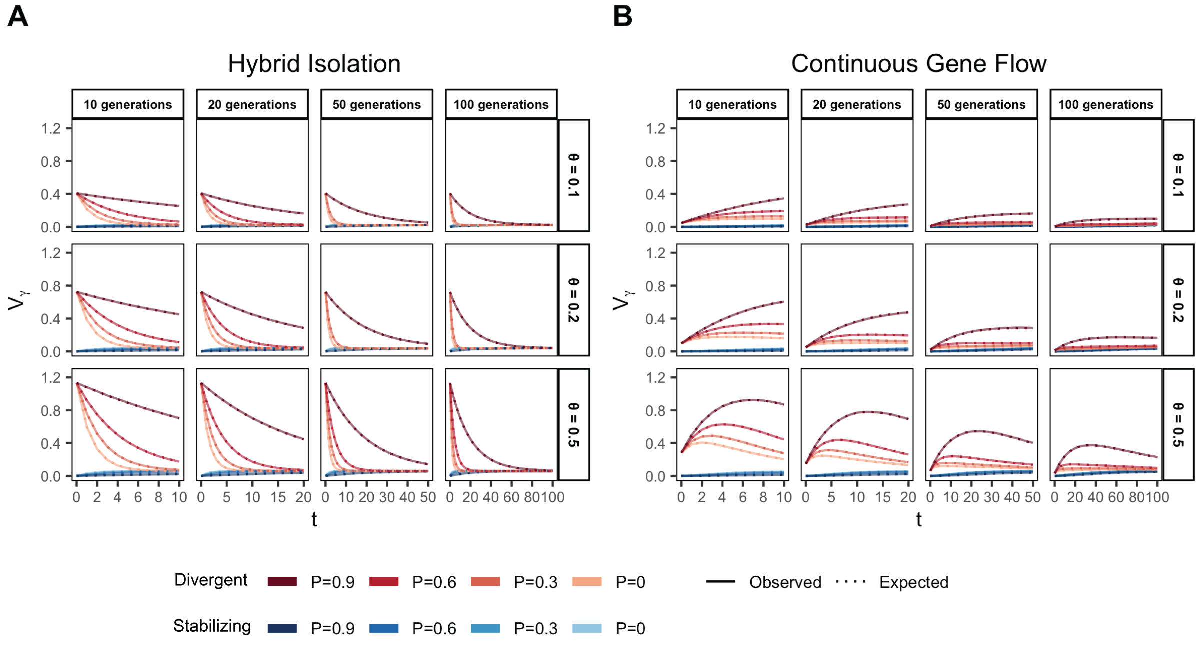

Figure S2: The behavior of the genetic variance due to local ancestry in admixed populations under the (A) HI and (B) CGF models. The solid lines represent values observed in simulations averaged across ten replicates and the dotted lines represent the expected values based on Eq. 1 of the main text. The red and blue lines represent values for traits 1 and 2, respectively. P indicates the strength of assortative mating. P=0.6 is missing for simulations run for 50 and 100 generations and $\theta \in \{0.1, 0.2\}$ due to the difficulty in finding mate pairs (Methods).

bioRxiv preprint doi: https://doi.org/10.1101/2023.08.04.551959; this version posted November 7, 2023. The copyright holder for this preprint (which was not certified by peer review) is the author/funder, who has granted bioRxiv a license to display the preprint in perpetuity. It is made available under aCC-BY 4.0 International license.

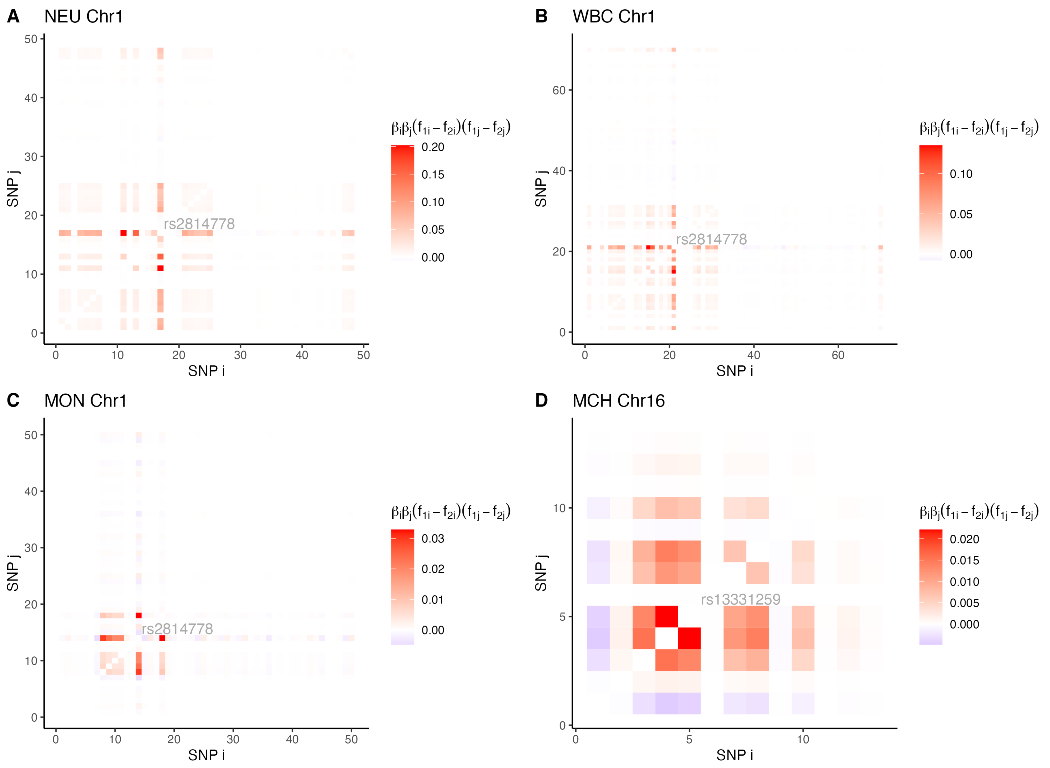

Figure S3: The LD contribution to the variance explained by variant pairs for (A) neutrophil counts (NEU), (B) white blood count (WBC), (C) monocyte count (MON), and (D) mean corpuscular hemoglobin (MCH). Only chromosomes where we suspected there was a disproportionate contribution to the variance explained are shown.

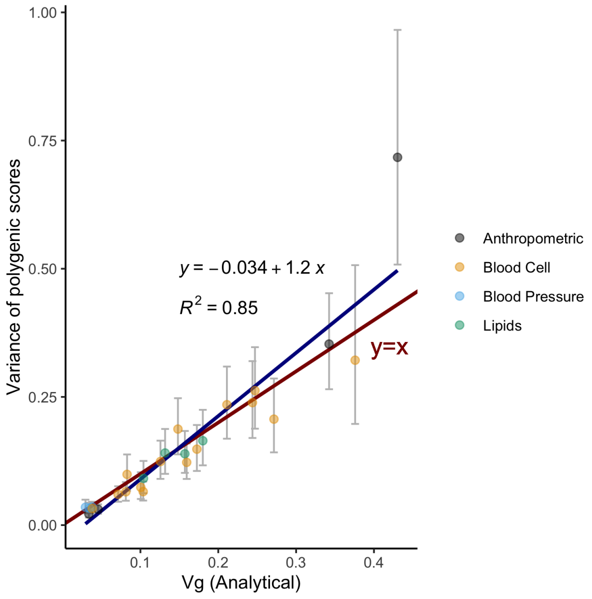

Figure S4: Expected variance explained estimated using Equation 1 (x-axis) vs the observed variance in polygenic scores (y-axis) in ASW are strongly correlated. Confidence intervals were generated by non-parametric bootstrap.
